# Design, synthesis, and biological evaluation of tetrahydroquinolinones and tetrahydroquinolines with anticancer activity

**DOI:** 10.1038/s41598-022-13867-x

**Published:** 2022-06-15

**Authors:** Małgorzata Ryczkowska, Natalia Maciejewska, Mateusz Olszewski, Milena Witkowska, Sławomir Makowiec

**Affiliations:** 1 Department of Organic Chemistry, Faculty of Chemistry, Gdansk University of Technology, Narutowicza 11/12, 80-233 Gdańsk, Poland; 2 Department of Pharmaceutical Technology and Biochemistry, Faculty of Chemistry, Gdansk University of Technology, Narutowicza 11/12, 80-233 Gdańsk, Poland

**Keywords:** Drug discovery, Cancer, Organic chemistry

## Abstract

Colorectal cancer (CRC) is the most commonly diagnosed cancer in Europe and the United States and the second leading cause of cancer related mortality. A therapeutic strategy used for the treatment of CRC involves targeting the intracellular levels of reactive oxygen species (ROS). In this study, we synthesized a series of novel tetrahydroquinolinones and assessed their ability to inhibit CRC growth and proliferation by evoking cellular stress through ROS. Our results revealed that (2-oxo-4-phenyl-5,6,7,8-tetrahydroquinolin-8-yl) N-(3-fluorophenyl)carbamate (**20d**) exhibited in vitro antiproliferative activity at micromolar concentrations. The compound also suppressed colony formation and the migration of HCT-116 cells, as well as deregulated the expression of several proteins involved in cell proliferation and metastasis. Furthermore, **20d** induced massive oxidative stress by disrupting the balance of cells survival resulting in autophagy via the PI3K/AKT/mTOR signaling pathway. These findings suggest that this tetrahydroquinolinone can be an ideal lead compound for drug discovery based on quinone derivatives.

## Introduction

**Figure 1:**
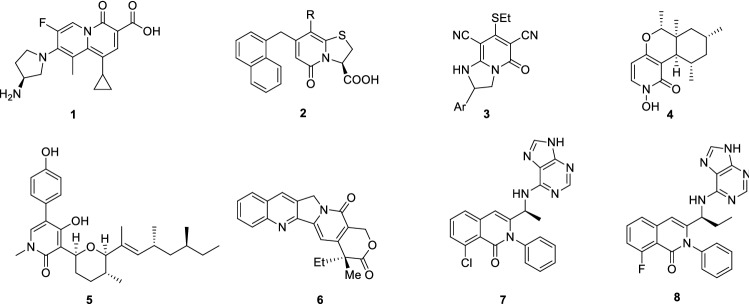
Examples of biologically active 2-pirydones.

Biologically active compounds containing the 2-pyridone system are considered valuable to their antimicrobial activity. Bicyclic 2-pyridones such as ABT-719 (**1**) (Fig. 1) exhibit excellent antibacterial properties with type II topoisomerases as a molecular target^1^. Pilicides (**2**), a specific type of 2-pyridones which display affinity to PapD and FimC chaperones, have been shown to effectively block pilus biogenesis and thus limit the virulence of uropathogenic *Escherichia coli* responsible for urinary tract infections^2–8^. Compound **3** containing a fused 2-pyridone and [1,2,4]-triazolo ring is characterized by antibacterial as well as antifungal activity^9^. PF-1140 (**4**), a naturally occurring 4-hydroxy-2-pyridone alkaloid, which is isolated from *Penicillium* sp., is another example of 2-pyridones with antifungal activity^10,11^. On the other hand, the derivatives of 2-pyridones also manifest anticancer activity^12^. A considerable number of studies focusing on the antitumor activity of 2-pyridone derivatives can be found in the chemical literature. Sambutoxin (**5**) isolated from *Hericium alpestre*^13^ and *Fusarium sambucinum* inhibits proliferation and induce apoptosis in cancer cells^14^. Camptothecin^15^ (**6**) and topotecan^16^ inhibit the activity of human DNA topoisomerase and cause the apoptosis of cancer cells^17^. However, the most interesting examples are the two PI3K (phosphatidylinositol-3-kinase) inhibitors approved for medicinal use—Duveslisib (**7**) and Idelalisib (**8**). Both these inhibitors possess rigid isoquinolinone.

**Figure 2:**
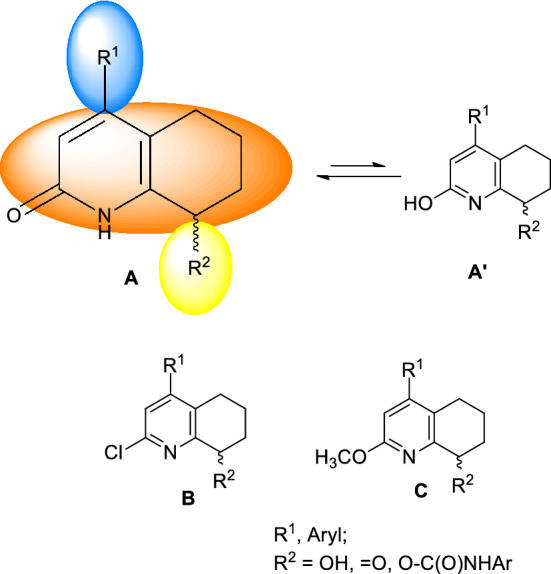
Structures of designed compounds.

The aforementioned facts inspired us to start searching for new anticancer compounds that are structurally based on modified tetrahydroquinolin-2(1H)-one. We aimed to find molecules containing shifted or inversed 2-pyridone moiety similar to pilicides or PI3K inhibitors. Thus, taking into account the Lipinski rule as well as the possibilities of synthesis, we designed a semirigid tetrahydroquinolin-2(1H)-one scaffold (**A**) substituted at position 4 with a hydrophobic moiety, whereas at position 8 moderately hydrophilic group like keto, hydroxyl, or N-substituted urethane was introduced. For 2-pyridone derivatives, it is necessary to consider their equilibrium with 2-hydroxypyridine form **A′**, which should change into a 2-pyridone form in a water solution^18,19^. Furthermore, considering the possible synthesis pathways, we decided to synthesize and verify the biological properties of two additional branches of derivatives: 2-chlorotetrahydroquinoline (**B**) and 2-methoxytetrahydroquinoline (**C**) (Fig. 2).

## Results

### Docking study

**Figure 3:**
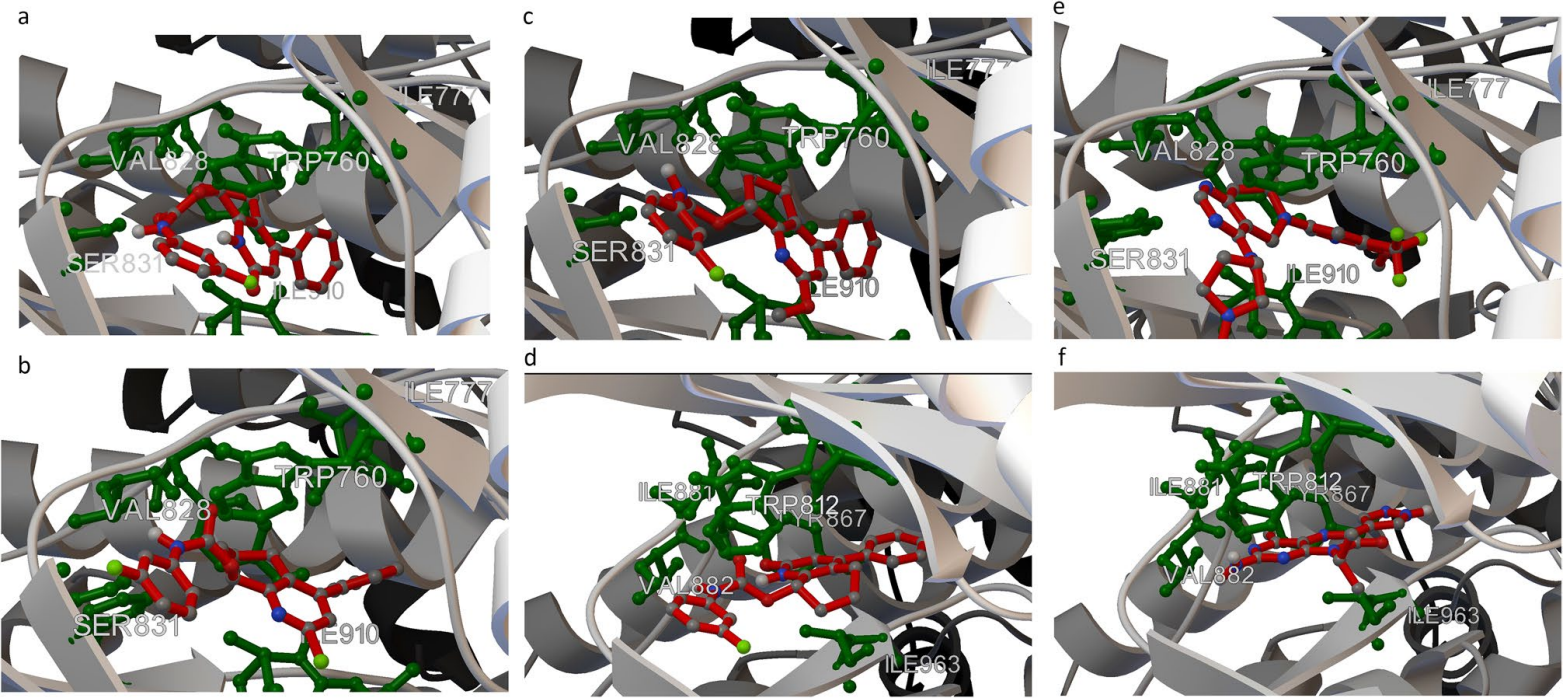
Results of molecular docking. (**a**) Binding mode of (R)-**20d** to PI3K δ. (**b**) Binding mode of (S)-**18d** to PI3K δ. (**c**) Binding mode of (R)-**19d** to PI3K δ. (**d**) Binding mode of (S)-**20d** to PI3K γ. (**e**) Binding mode of Leniolisib to PI3K δ. (**f**) Binding mode of 2-amino-4-methyl-8-(1-methylethyl)-6-(1H-pyrazol-4-yl)pteridin-7(8H)-one to PI3K γ.

The assessment of the biological activity of the proposed structures as well as the preselection of molecules for synthesis was performed using AutoDock Vina^20^ and AutoDock4.2 software packages^21,22^. As mentioned above, 2-pyridones with potential anticancer activity often act on PI3K, which are responsible for cellular growth and survival signals and thus regulate tumor growth and expansion. Therefore, for the evaluation of our molecules, we chose PI3K from the PI3K/protein kinase B (AKT)/mammalian target of rapamycin (mTOR) pathway. In silico calculation was performed using the crystallographic structure of two kinase isoforms—human γ 3OAW^23^ and mouse δ 5O83^24^ PI3K—obtained from PDB. We used the PI3K δ kinase complexed with Leniolisib, a well known and selective PI3K δ inhibitor, to verify if Leniolosib will dock at the same position as was observed in the crystallographic structure. The analysis showed a positive result, and docked Leniolisib overlapped the molecule present in the crystallographic structure, with a binding energy value of − 9.13 kcal/mol (RMSD 38.34 Å) and inhibition constant value of 203.87 nM. Thus, we carried out a docking study with our designed ligands. In the first attempt, we docked a series of compounds (**20**) to PI3K δ. An overview of the estimated binding energy values pointed out that the highest affinity to PI3K δ was shown by the molecules with N-aryl urethane moiety (**20**). The lowest binding energy (− 10.11 kcal/mol) (RMSD 39.06 Å), as well as inhibition constant value (38.9 nM) was observed for (R)-**20d**. (R)-**20d** exhibited hydrophobic interactions with TRP 760, ILE 777, TYR 813, ILE 825, PHE 908, ILE 910, and ASP 911. Hydrogen bond formation was observed between the urethane moiety of (R)-**20d** and VAL 828 and SER 831 (Fig. 3a).

Next, we evaluated a series of chloropyridine derivatives (**18**) to determine their binding mode. Even in this case, N-aryl urethanes exhibited the highest affinity to kinase. Compound (S)-**18d** had the lowest binding energy in this series (− 10.88 kcal/mol) (RMSD 39.28 Å) and a lower inhibition constant (10.6 nM) than 2-pyridone derivative. The binding mode of (S)-**18d** is presented in Fig. 3b. The compound showed hydrophobic interactions with TYR 813, ILE 825, PHE 908, and ILE 910. In addition, hydrogen bond formation was observed between the urethane moiety of (S)-**18d** and VAL 828, and π-stacking between TRP 760 and 3-fluorophenyl ring. We also tested the binding mode of a series of methoxypyridine derivatives (**19**). Interesting docking results were observed for (R)-**19d** (Fig. 3c) with a binding energy value of − 10.53 kcal/mol (RMSD 38.45 Å) and inhibition constant value of 19.1 nM. Similar hydrophobic interactions were observed between TYR 813, ILE 825, and ILE 910, and additional interaction between ILE 777 with a cyclohexyl ring and ASP 911 with a phenyl ring. Urethane formed hydrogen bond with VAL 828 and additionally with SER 831. In addition, π-stacking was observed between TRP 760 and 3-fluorophenyl ring.

In the next step, we checked the modes of binding of our designed ligands to PI3K γ. We noted that interaction with protein was not as effective as that observed for PI3K δ; however, the binding energy calculated for (S)-**20d** (binding energy: − 10.08 kcal/mol, (RMSD 52.84 Å) inhibition constant: 40.7 nM) was better than that of Leniolisib. A similar pattern of interactions was observed: hydrophobic interactions with ILE 831, TYR 867, ILE 879, ILE 881, ILE 963, and ASP 964; hydrogen bond between the urethane moiety of (S)-**20d** and VAL 882, and a π-stacking interaction between TRP 812 and 3-fluorophenyl ring, as well as between TYR 867 and 2-pyridone ring (Fig. 3d). Binding modes of re-docked Leniolisib to PI3K δ and 2-amino-4-methyl-8-(1-methylethyl)-6-(1H-pyrazol-4-yl)pteridin-7(8H)-one to PI3K γ as reference compounds are presented on Fig. 3e,f respectively.

Summing up, our in silico calculation confirmed that our tetrahydroquinolin-2(1H)-ones showed a consistent mode of interaction with kinase. The following parts of the ligand were of key importance in the interactions: 3-fluorphenyl ring for π-stacking, urethane for hydrogen bond with VAL 828, and bicyclic tetrahydroquinoline moiety for hydrophobic interaction. Moreover, docking showed that all considered structures exhibited a higher affinity to the active site of PI3K δ than for the reference compound Leniolisib. These results prompted us to synthesize a series of new tetrahydroquinolin-2(1H)-ones and evaluate their biological activity.

### Chemistry

#### Synthesis of tetrahydroquinoline derivatives

**Scheme 1:**
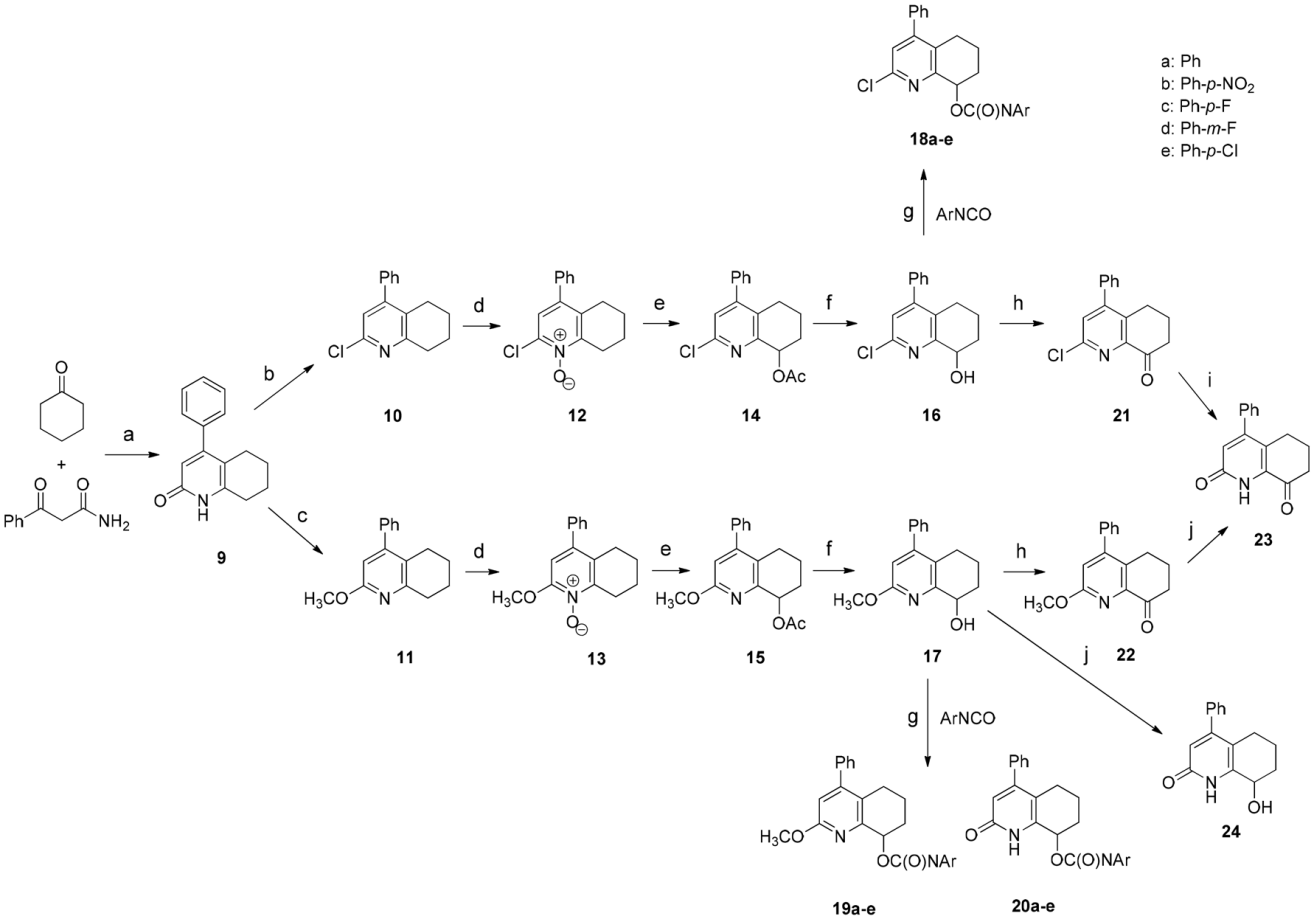
The synthetic pathways during preparation of potential PI3K kinase inhibitors: (**a**) TsOH/toluene, reflux; (**b**) PhP(O)Cl_2_, 160 °C; (**c**) Ag_2_CO_3_,CH_3_I/CHCl_3_, RT; (**d**) 30% H_2_O_2_/AcOH, 80 °C; (**e**) Ac_2_O, RT to 100 °C, 5 h; (**f**) KOH/MeOH, RT; (**g**) TEA/DCM, RT; (**h**) DSMO, (COCl)_2_,TEA/DCM, Ar, − 78 °C to RT; (**i**) 12 M HCl aq., reflux; (**j**) NaI/AcOH, reflux;

We synthesized the new PI3K inhibitors through the following pathway (Scheme 1). In the first step, we used a modified procedure for the synthesis of tetrahydroquinolin-2(1H)-one core (**9**)^25,26^. During condensation, we found out that it is additionally necessary to remove water formed with anhydrous MgSO_4_. Since it was not possible to perform direct functionalization of **9** at position 8, we had to transform tetrahydroquinolin-2(1H)-one (**9**) into 2-chlorotetrahydroquinoline (**10**) and 2-methoxytetrahydroquinoline (**11**) via typical chlorination with PhP(O)Cl_2_^27^ and alkylation with silver carbonate^28^. Compounds **10** and **11** were oxidized at position 8. However, this two-step process required the formation of N-oxides (**12**, **13**) which subsequently underwent intramolecular disproportionation into 8-acetoxy derivatives (**14**, **15**). The acetyl group was removed by alkaline hydrolysis. Structures **16** and **17** were important for multiway functionalization. The first pathway led to the formation of 2-chloro-4-phenyl-5,6,7,8-tetrahydroquinolin-8-yl arylcarbamates (**18a–e**) by the addition of alcohol **16** to various isocyanates in the presence of tertiary amine. Formation of 6,7-dihydroquinolin-8(5H)-one derivatives (**21**, **22**) required to perform Swern oxidation. The compound 4-phenyl-6,7-dihydroquinoline-2,8(1H,5H)-dione (**23**) might be independently formed through acidic hydrolysis of **21** or hydrolysis of **22** with acetic acid catalyzed by NaI. It must be noted that 8-hydroxytetrahydroquinolin-2(1H)-one (**24**) can only be formed from **17** via hydrolysis with AcOH and NaI, because acid hydrolysis of 2-chloro-tetrahydroquinolin-8-ol (**16**) led to the formation of a dehydrated product. However, the results of our subsequent experiments indicated that the preparation of intermediate **24** was not necessary at all. From the reaction mixtures consisting of **17**, triethyl amine, and appropriate isocyanate, we isolated a carbamate derivative of the parent compound (**19**) together with a significant amount of 2-oxo-4-phenyl-1,2,5,6,7,8-hexahydroquinolin-8-yl arylcarbamate (**20**). Generally, the amount of secondary products formed depends on the used isocyanate; however, we observed the aforementioned phenomenon. We experimentally evaluated several possible ways for demethylation.

To track the migration of the methyl group, we prepared **17** with a ^13^C-labeled methyl group. We introduced the labeled methyl group using commercially available ^13^CD_3_I as an alkylating agent. Surprisingly, our attempts to perform an identical reaction between ^13^CD_3_-labeled **17** and *p*-nitrophenyl isocyanate in the presence of amine were not successful. ^13^CD_3_-labeled **17** does not undergo demethylation. The first obvious solution was to remove the methyl group by a nucleophilic attack, for example, using water. However, during nuclear magnetic resonance-monitored reaction with nonlabeled **17**, we did not observe any CH_3_ signal of methanol in the reaction mixture using a pyridine base to uncover the aliphatic part of the spectrum. The thin-layer chromatographic analysis of this reaction mixture revealed the formation of the demethylated product **20**. We also observed that strong exclusion of water from the reaction mixture during the process may inhibit demethylation, which may be due to, for example, the molecular sieves present in the solvent. Therefore, we postulated that demethylation is caused by traces of water. Additionally, we found that compound **19**, which was formed already, did not undergo demethylation, even if it was treated with a fresh portion of isocyanate. Similarly, pure **17** was not demethylated in solution in the absence of isocyanate.

**Scheme 2:**
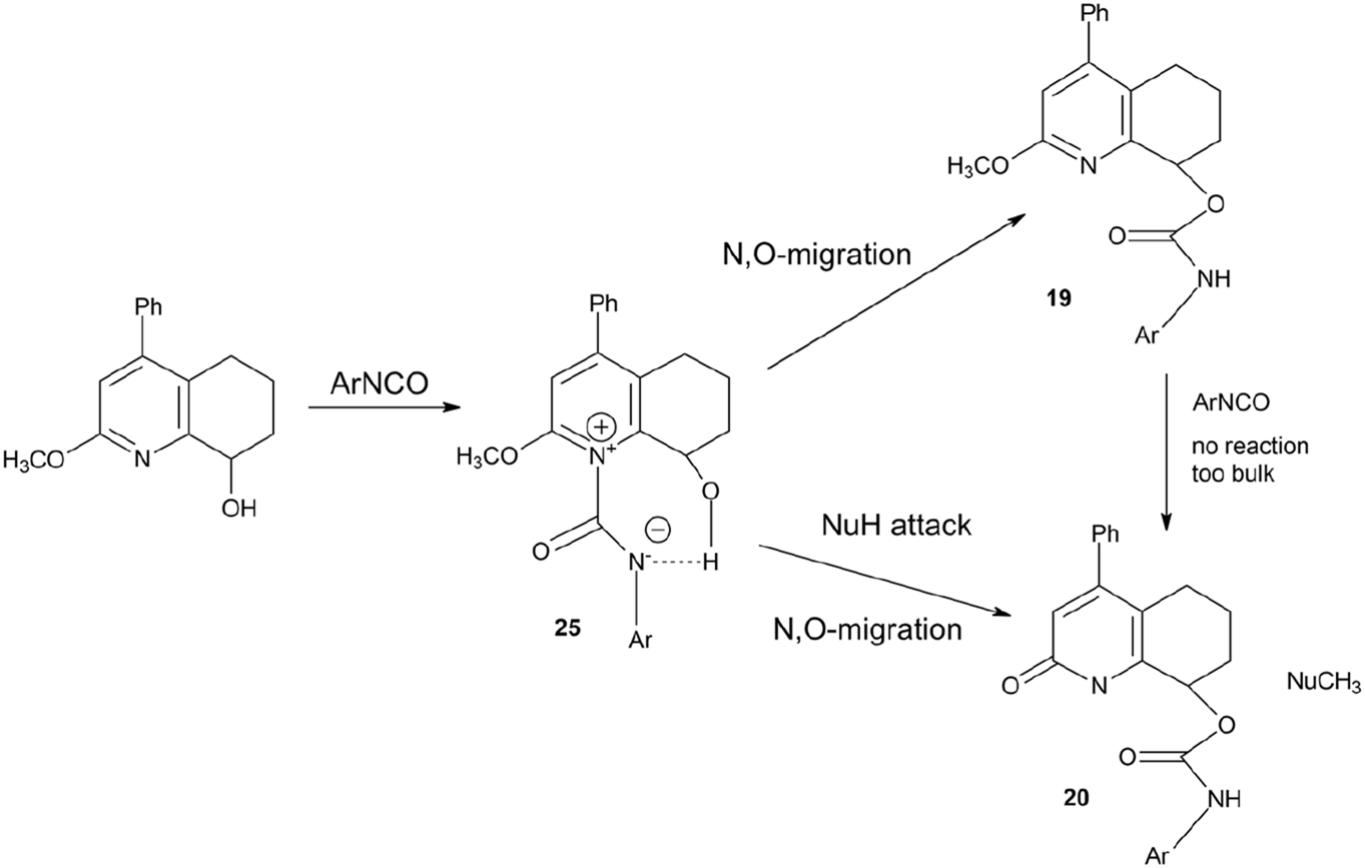
Tentative demethylation reaction mechanism.

Nevertheless, although the mechanism of demethylation is still unclear, we assumed a tentative reaction mechanism to elucidate the observed process (Scheme 2).

At the first step, 2-methoxy-4-phenyl-5,6,7,8-tetrahydroquinolin-8-ol (**17**) is acylated with isocyanate on the nitrogen atom. The resulting intermediate **25** is susceptible to nucleophilic attack on the methyl group due to the positive charge of nitrogen. In the next step, the migration of isocyanate moiety from pyridinium nitrogen to alcoholic oxygen is carry out in order to terminate the process. This step might be accompanied by simultaneous nucleophilic attack of water on the methyl group, leading to the formation of demethylation product **20**. The already formed product **19** did not undergo demethylation because nitrogen atom is associated with steric hindrance, which excludes the possibility of its reaction with isocyanate.

Thus, the complete lack of demethylation in experiments with ^13^CD_3_-labeled **17** strongly suggests the existence of reversed secondary isotope effects, which can influence the reaction rate, and indicates that the observed demethylation reaction should be of Sn2 type or a similar type involving modified sp^3^/sp^2^ hybridization of the methyl group.

### Biological activity

#### Tetrahydroquinoline derivatives displays potent cytotoxic and antiproliferative activities

**Figure 4:**
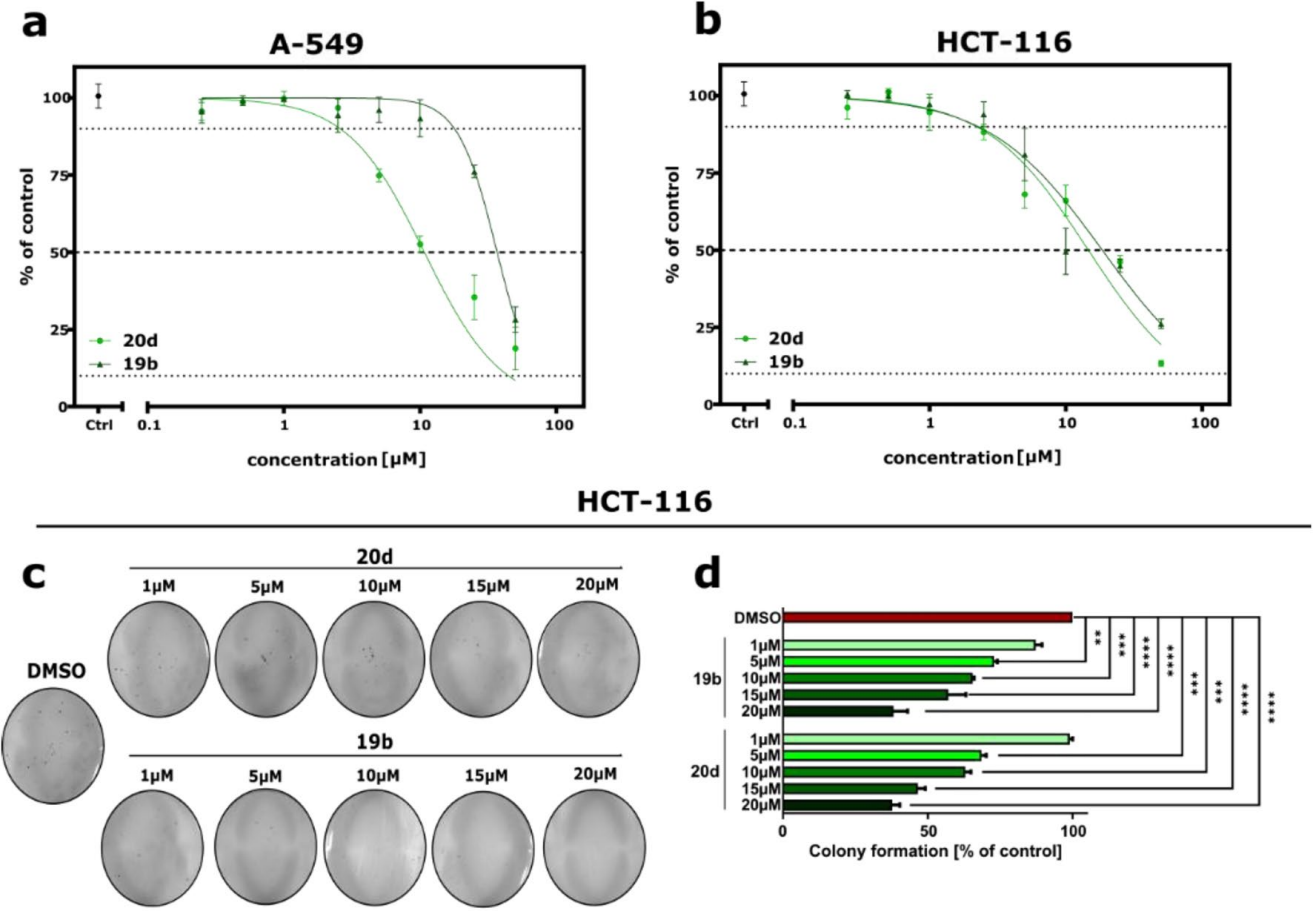
Dose–response curves of **20d** and **19b** in A-549 (**a**) and HCT-116 (**b**) after 72 h of treatment cells determined by the MTT assay. All data are presented as mean ± SEM of three independent experiments in triplicates. (**c**,**d**) The effect of the examined compound on the colony-forming ability of HCT-116 cells. (**c**) Representative images of colonies formed by HCT-116 cells after treatment with different concentrations of **20d** and **19b**. (**d**) Quantitative analysis of clonogenicity assays. Error bars represent the SEM of data obtained from three independent experiments. **p < 0.001, ***p < 0.0001, and ****p < 0.00001 vs. vehicle.

To evaluate the effects of the newly synthesized tetrahydroquinolin-2(1H)-one derivatives on cell viability, we treated human colon cancer cells HCT-116, human breast cancer cells MCF-7, and human non-small cell lung cancer cells A-549 with different concentrations of the compounds for 72 h. After incubation, the MTT (3-(4,5-dimethylthiazol-2-yl)-2,5-diphenyltetrazolium bromide) assay was performed and the cytotoxic potency of compounds (expressed in IC_50_) on the cells was determined in comparison to control cells treated with 1% of dimethyl sulfoxide (DMSO) (Table 1). Compounds **9**, **16**, **18b**, **18d**, **21**, **23**, **22**, **17**, **24**, and **20e** showed no cytotoxicity on tested cell lines at concentrations up to 50 µM. Other compounds showed moderate activity against A-549 and HCT-116 cell lines, while none of the compounds displayed cytotoxic effect on MCF-7 breast cancer cells. The IC_50_ values were estimated at the lowest micromolar concentration (cytotoxicity at ~ 13 µM) for compounds **19b**, **19c**, **19e**, **20a**, and **20d** in HCT-116 cells among the studied derivatives. While these derivatives showed pronounced cytotoxicity against colon cancer cells, only compounds **20d** and **19b** exhibited similar potency against A-549 lung cancer cells (Fig. 4a,b). We also compared the activity of **20d** and **19b** to the reference control drug Cisplatin, which is widely used in chemotherapy^29^. It was observed that the tested compounds were more effective and selective than Cisplatin in both HCT-116 and A-549 cell lines (Table 1), suppressing 50% of cell viability at 7.5- and 5-fold lower concentrations, respectively. Importantly, none of the investigated compounds caused a reduction in the viability of the nonmalignant human embryonic kidney cells (HEK293) at concentrations up to 50 µM. Next, we investigated the antiproliferative activity of **20d** and **19b** in HCT-116 cells using clonogenic assay and observed that both compounds effectively inhibited the formation of colonies in a concentration-dependent manner in this cell line (Fig. 4c,d).

**Table 1:** In vitro anticancer activity of investigated compounds (IC_50_ ± SD (µM)) towards human colon cancer (HCT-116), non-small cell lung adenocarcinoma (A-549), and human breast carcinoma (MCF-7), human and embryonic kidney cells (HEK293).

Compound	Cell lines				SI^b^
	HCT-116	A-549	MCF-7	HEK293	
	IC_50_ [µM]^a^				
**9**	> 50	> 50	> 50	> 50	ND
**16**	> 50	> 50	> 50	> 50	ND
**17**	> 50	> 50	> 50	> 50	ND
**18a**	39.83 ± 2.62	27.24 ± 1.53	> 50	> 50	1.55
**18b**	> 50	> 50	> 50	> 50	ND
**18c**	18.93 ± 1.26	23.83 ± 4.02	> 50	> 50	6.2
**18d**	> 50	> 50	> 50	> 50	ND
**18e**	> 50	45.33 ± 4.20	> 50	> 50	ND
**19a**	26.17 ± 1.69	> 50	> 50	> 50	1.99
**19b**	13.49 ± 0.20	15.69 ± 2.56	> 50	> 50	4.07
**19c**	12.96 ± 2.68	28.44 ± 0.56	> 50	> 50	2.51
**19d**	31.64 ± 0.58	41.07 ± 0.93	> 50	> 50	1.50
**19e**	13.88 ± 1.30	> 50	> 50	> 50	3.79
**20a**	13.11 ± 1.55	21.79 ± 0.22	> 50	> 50	2.91
**20b**	> 50	> 50	> 50	> 50	ND
**20c**	18.44 ± 2.04	23.83 ± 4.02	> 50	> 50	ND
**20d**	12.04 ± 0.57	12.55 ± 0.54	> 50	> 50	20.68
**20e**	> 50	> 50	> 50	> 50	ND
**21**	> 50	> 50	> 50	> 50	ND
**22**	> 50	> 50	> 50	> 50	ND
**23**	> 50	> 50	> 50	> 50	ND
**24**	> 50	> 50	> 50	> 50	ND
**Cisplatin**	24.89 ± 1.14	29.01 ± 0.12	33.80 ± 1.56	28.45 ± 1.97	1.05

^a^IC_50_ value represent a concentration that inhibits 50% of cell growth.

^b^SI value represent selectivity indexes (IC_50 HEK-293_/Average IC_50 cancer cell lines_); ND: not determined, since the compound was inactive in the experimental conditions.

**Figure 5:**
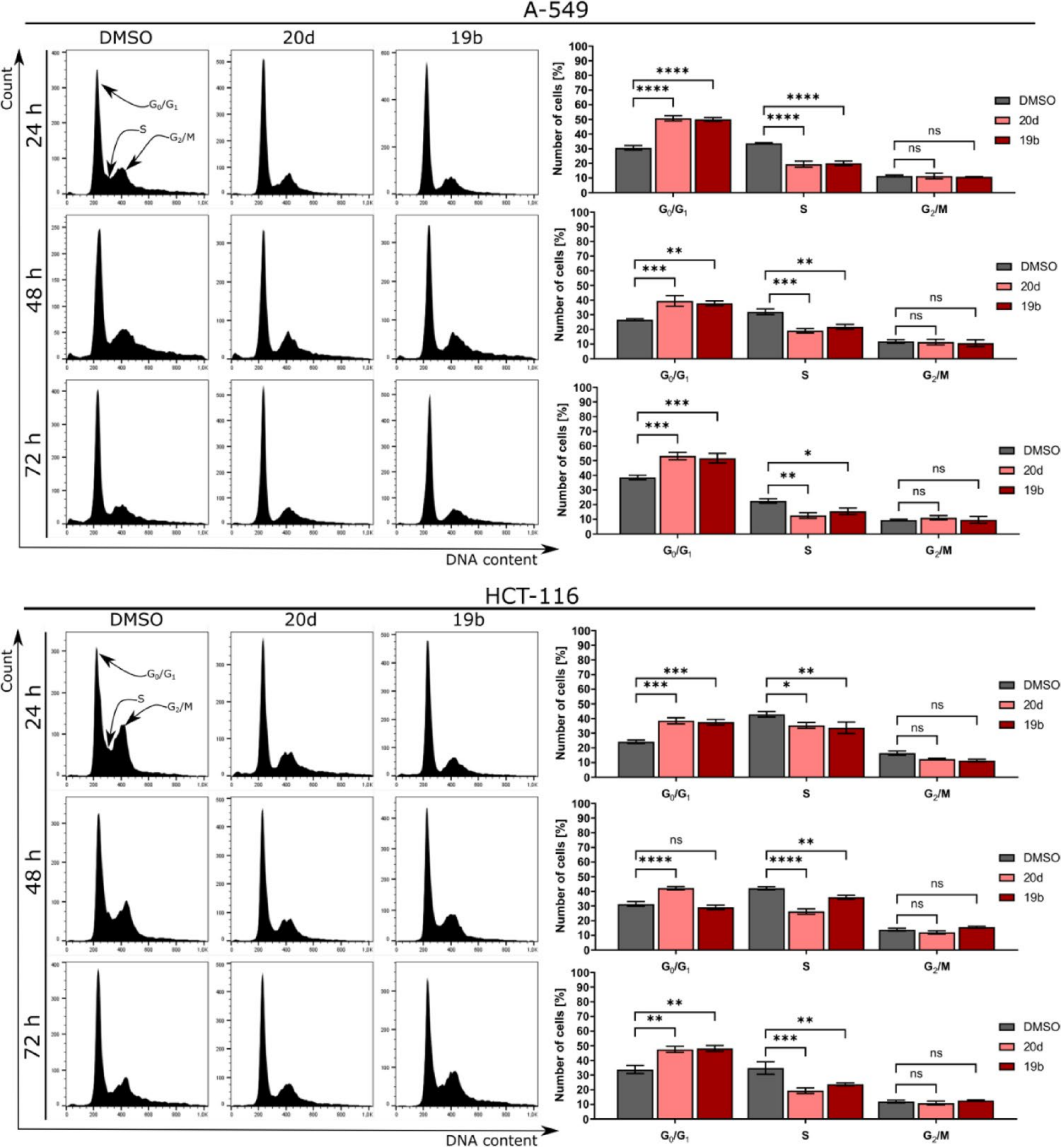
Cell cycle analyses. Representative histograms after DNA staining are presented in left panels. Statistical analyses of histograms are presented on right panels. Error bars show the SEM of data obtained in three independent experiments. Statistical differences were analyzed with a one-way ANOVA post-hoc Dunnet’s test. ns p > 0.05, *p < 0.01, **p < 0.001 ***p < 0.0001 vs. vehicle.

Based on their antiproliferative activity, **20d** and **19b** were evaluated for their effect on cell cycle phase distribution and DNA content by flow cytometry. The compounds induced alterations in cell cycle distribution in HCT-116 cells at every time-point of treatment. In comparison to vehicle, treatment with compounds **20d** and **19b** for 24 to 72 h led to a time-dependent increase (up to < 14%) in the number of cells in the G_0_/G_1_ phase (Fig. 5). Concomitantly, a substantial decrease in the number of cells in the S-phase as well as negligible change in the sub-G_1_ and G2/M fractions was observed, especially in the case of **20d**.

Considering its interesting in vitro antiproliferative activity and the strong inhibitory effect on cell cycle progression, we selected compound **20d** for the further analysis of the mechanism of its molecular action.

### 20d induces generation of ROS in HCT-116 cells

**Figure 6:**
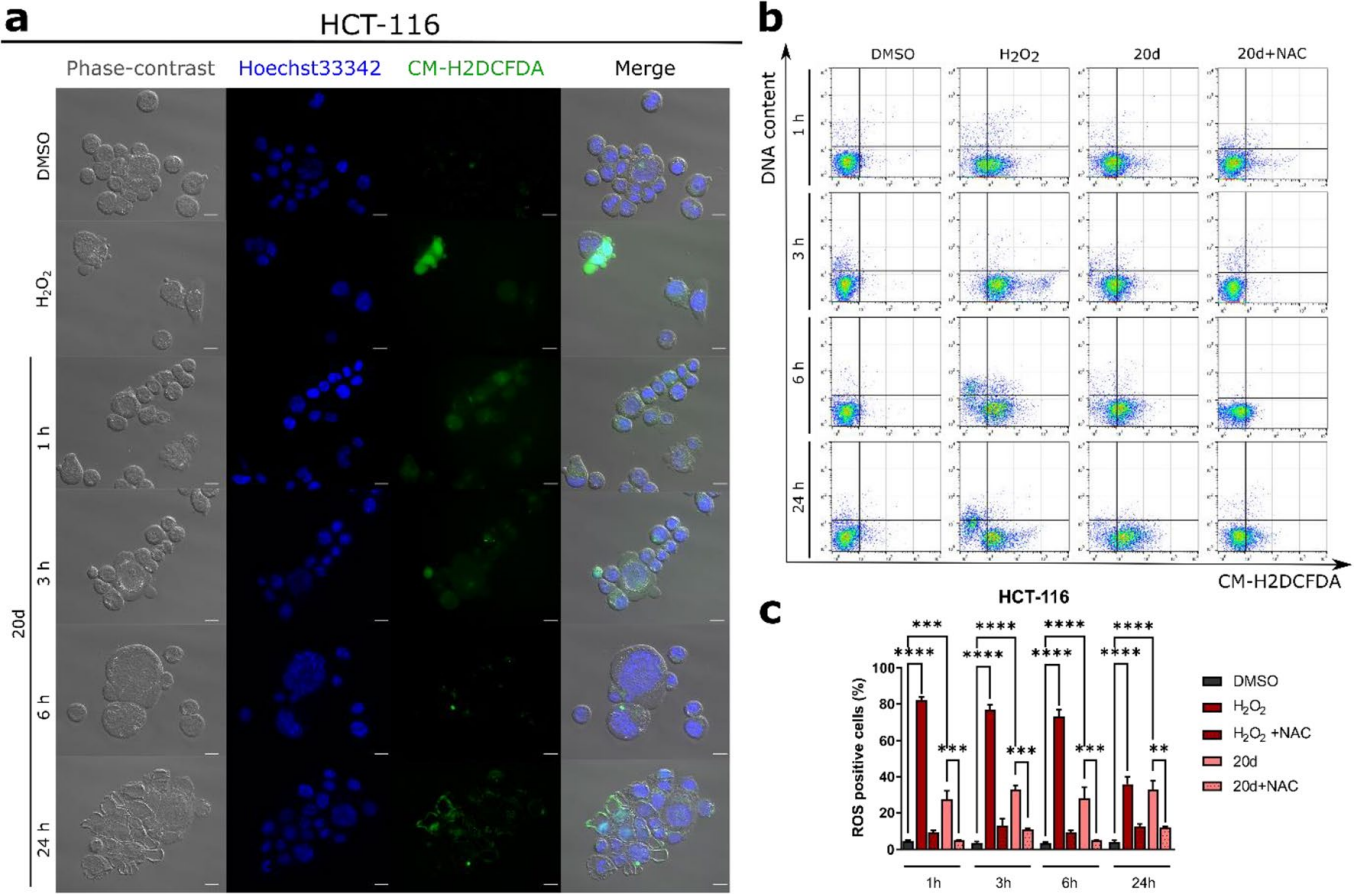
Effect of **20d** treatment on ROS induction in HCT-116 cells after 1, 3, 6, and 24 h of exposure. (**a**) Representative microscopic images of H2DCFDA-stained cells. Scale bar = 50 µm. (**b**) Representative dot plots of H2DCFDA-stained HCT-116 cells. DMSO and H_2_O_2_ were used as reference compounds. (**c**) Quantification of dot plots is depicted as mean ± SEM of data obtained from three independent experiments.

Reactive oxygen species (ROS) affect the cellular redox homeostasis resulting in extensive and irreparable damage and ultimately cell death^30^. Anticancer compounds with cytotoxic properties often cause disturbances in the redox state in cells. Therefore, we performed a time-dependent kinetic analysis of the intracellular ROS level in colon cancer cell lines after treatment with **20d**. As depicted in Fig. 6, the compound induced the generation of a high amount of ROS in HCT-116 cells, but not in A-549 cell line, independently of the exposure time (~ 30% and 5% increase in ROS level compared to vehicle).

We further attempted to determine whether **20d**-induced cancer cell death was related to the induction of ROS production in HCT-116 cells. For this purpose, we pretreated cells with N-acetyl-l-cysteine (NAC), which is a commonly used antioxidant and ROS scavenger. As shown in Fig. 6b,c, NAC diminished the cytotoxic effect of **20d** in HCT-116. This indicated that ROS-dependent cellular redox imbalance is the main mechanism of **20d**-mediated cell death in this cell line. HCT-116 cell line has high-frequency microsatellite instability, which is associated with deficient DNA mismatch repair (MMR)^31^. The MMR system corrects DNA-mismatched and insertion–deletion loop bases generated during DNA replication^32^. One of the key proteins involved in the MMR pathway is MLH1, which is mutated or epigenetically silenced in many MMR-deficient (MMR^–^) cells, such as HCT-116^33^. To further determine the selectivity for MMR deficiency, we analyzed the cell viability of human MutL homologue 1 (hMLH1)-deficient (MMR^–^) and hMLH1-proficient (MMR^+^) RKO human colon carcinoma cell lines. The latter was established by transfecting hMLH1 cDNA into MMR^−^ RKO cell line^34^. In MMR^–^ RKO cells, the hMLH1 gene promoter was silenced transcriptionally by hypermethylation^35^. The viability assay revealed that MMR^−^ RKO cells were 3.95-fold more sensitive to **20d** (*p* < 0.001) compared to the MMR^+^ RKO cells (Fig. S55 in supplementary information), and the respective IC_50_ values (mean ± SD) were 1.21 ± 0.13 and 4.78 ± 0.38 μM.

### **20d** oxidative properties are related to MMR deficiency

**Figure 7:**
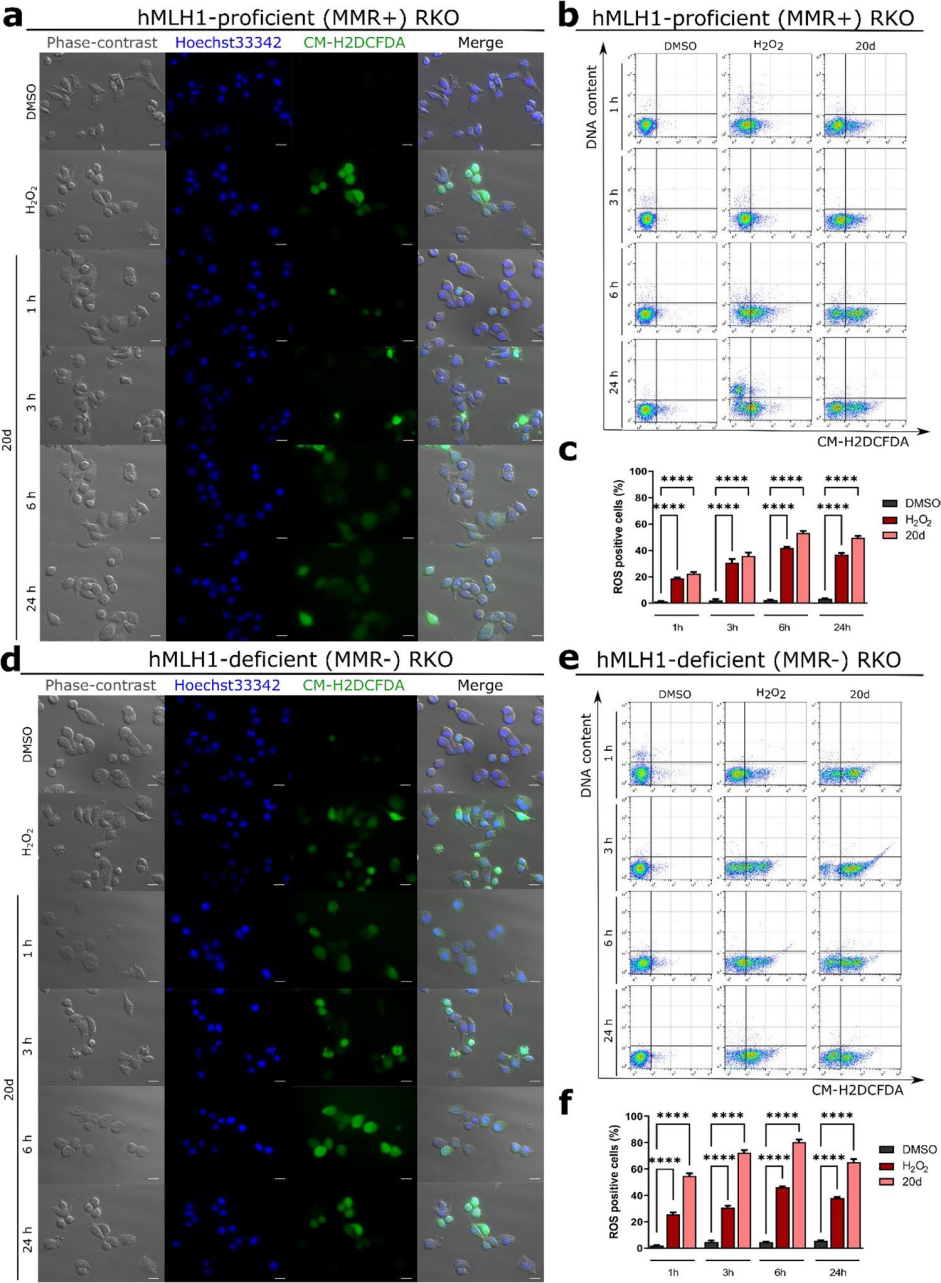
Effect of **20d** treatment on ROS induction in hMLH1-proficient and hMLH1-deficient RKO cells after 1, 3, 6, and 24 h of exposure. (**a**,**d**) Representative microscopic images of H2DCFDA-stained cells. Scale bar = 50 µm. (**b**,**e**) Representative dot plots of H2DCFDA-stained RKO cells. DMSO and H_2_O_2_ were used as reference compounds. (**c**,**f**) Quantification of dot plots is depicted as mean ± SEM of data obtained from three independent experiments.

Then, we confirmed whether the induction of cellular stress by **20d** is related to MMR deficiency by exposing MMR-deficient and MMR-proficient cells to the compound for different times and measuring the ROS levels. Our results indicated a greater increase in the level of ROS after 1 h of exposure in **20d**-treated MMR-deficient cells than in MMR-proficient cells (54.71 ± 2.70% and 22.27 ± 1.7%, respectively), in comparison to control (Fig. 7). This finding suggests that **20d** could modulate oxidative stress in human MLH1-defective colon cancer cells through synthetic lethality.

### **20d** treatment induces autophagy cell death in HCT-116 cells through the PI3K/AKT/mTOR signaling pathway

Due to its ability to mediate the redox signaling pathways, ROS interplays between two major types of programmed cell death: autophagy and apoptosis^36^. One of the early events in the process of apoptosis is the translocation of phosphatidylserine to the exposed membrane surface, which can be detected using Annexin V, a protein with a high affinity to this phospholipid^37^. In order to study the impact of **20d** on apoptotic cell death, flow cytometry analyses with double-staining using Annexin V-fluorescein isothiocyanate (FITC) conjugate and propidium ioidide (PI) was performed. As shown in Fig. S55 in the supplementary information, percentage of both early (Annexin V(+)/PI(−)) and late (Annexin V(+)/PI(+)) apoptotic as well as necrotic (Annexin V(−)/PI( +)) fractions, after treatment with HCT-116 cells incubated with **20d**, were on the same level as in DMSO-treated group. This result indicates that **20d** induced apoptosis- and necrosis-independent cell death in HCT-116 cells.

**Figure 8:**
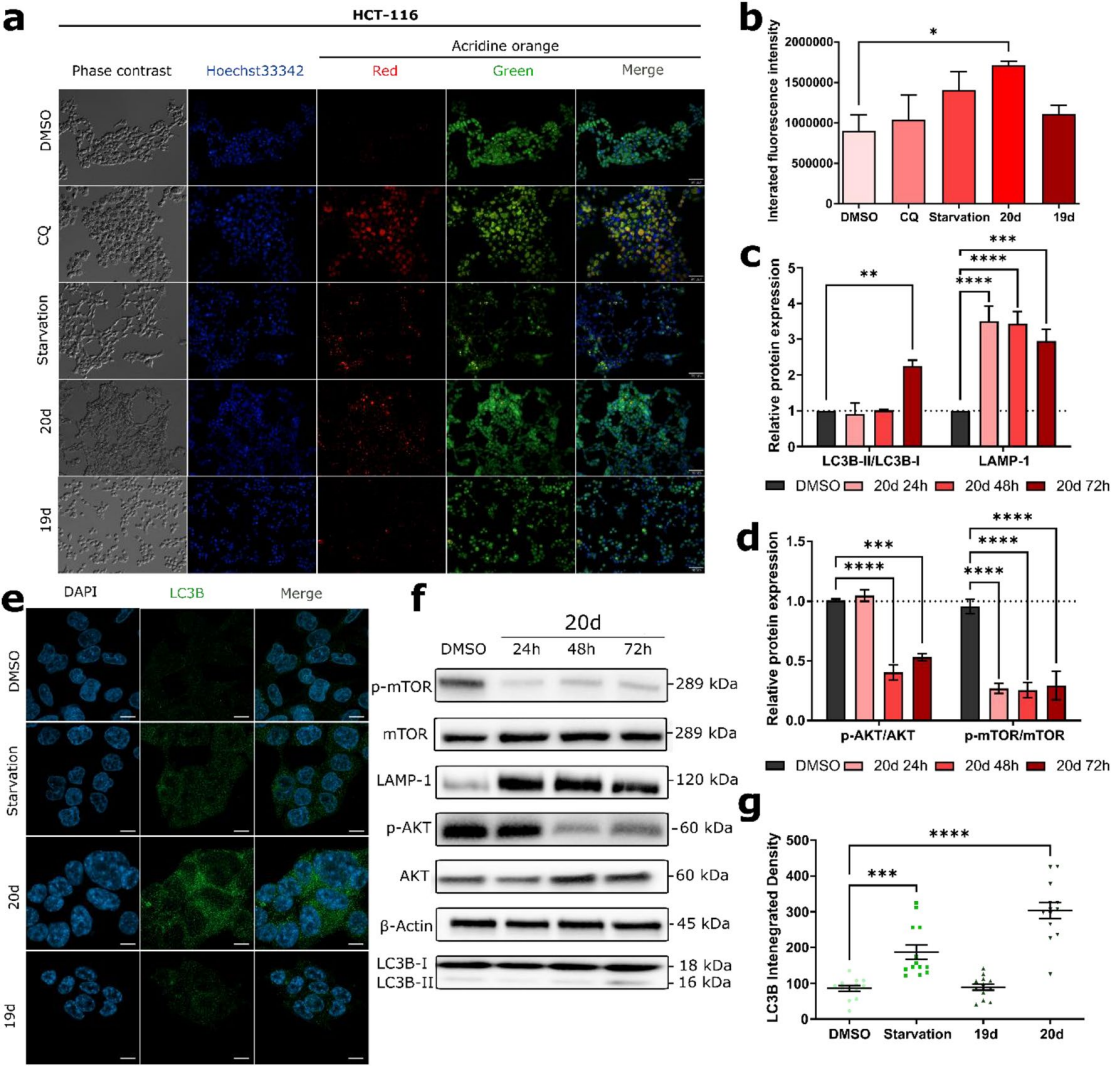
(**a**) Representative microscopic images of Hoechst33342- and AO-stained HCT-116 cells. Scale bar = 50 µm. Chloroquine was used as a reference. (**b**) Quantification of microscopic images, presenting the integrated red fluorescence intensity, depicted as mean ± SEM of data obtained from 10 random fields of view. (**c**,**d**,**f**) The expression of various proteins detected by Western blot assay after HCT-116 treatment with **20d** or DMSO. The level of each protein was normalized to that of β-actin. Error bars represent the data ± SEM (**e**) Representative microscopic images of HCT-116 cells. LC3B is depicted in green and nucleus in blue (DAPI). Scale bar = 10 µm (**g**) Quantification of microscopic images showing the integrated density of LC3B presented as mean ± SEM of data obtained from 10 random fields of view.

Then, to elucidate whether compound **20d** can act as an autophagy modulator, we stained cells with acridine orange (AO), a lysotropic agent^38^. AO is a weak base that can permeate the cell membrane in an uncharged state emitting green fluorescence. After protonation, AO forms aggregates in acidic vesicular organelles (AVOs), emitting red fluorescence^32^. As shown in Fig. 8a,b, treatment with **20d** significantly enhanced the accumulation of AVOs in HCT-116 cells, resulting in a 1.9-fold (*p* < 0.01) increase in red fluorescence in comparison to vehicle. One of the hallmarks of autophagy is the conjugation of LC3-I with phosphatidylethanolamine, followed by its conversion to autophagosome-associated form LC3-II^39^. Taking this into account, we evaluated the expression of LC3I/II in colon cancer cells after exposure to **20d**. Furthermore, we studied the expression of LAMP-1, a well-known lysosomal marker^40^. Immunoblot assays revealed that the levels of LC3-II were higher in **20d**-treated HCT-116 cells compared to control groups (Fig. 8c,f). This observation was also confirmed by increased punctate cytosolic LC3 fluorescence intensity when HCT-116 cells were assessed by confocal microscopy (Fig. 8e,g). In addition, a time-dependent increase in the expression of LAMP-1 protein was noted after **20d** treatment. It has been shown that multiple signaling pathways are involved in the modulation of autophagy, including the PI3K/AKT/mTOR pathway, which is especially involved in the stage of autophagosome elongation^41,42^. In this study, we investigated whether **20d**-induced autophagy in HCT-116 cells is related to PI3K/AKT/mTOR signaling. The results showed that **20d** significantly reduced the levels of phospho-AKT (Ser473) and phospho-mTOR (Ser2448) proteins, in a time-dependent manner (Fig. 8d,f). This proves that **20d** induced autophagy cell death in HCT-116 cells by interfering with the PI3K/AKT/mTOR signaling pathway.

### **20d** modulates expression of the cell cycle-related protein

**Figure 9:**
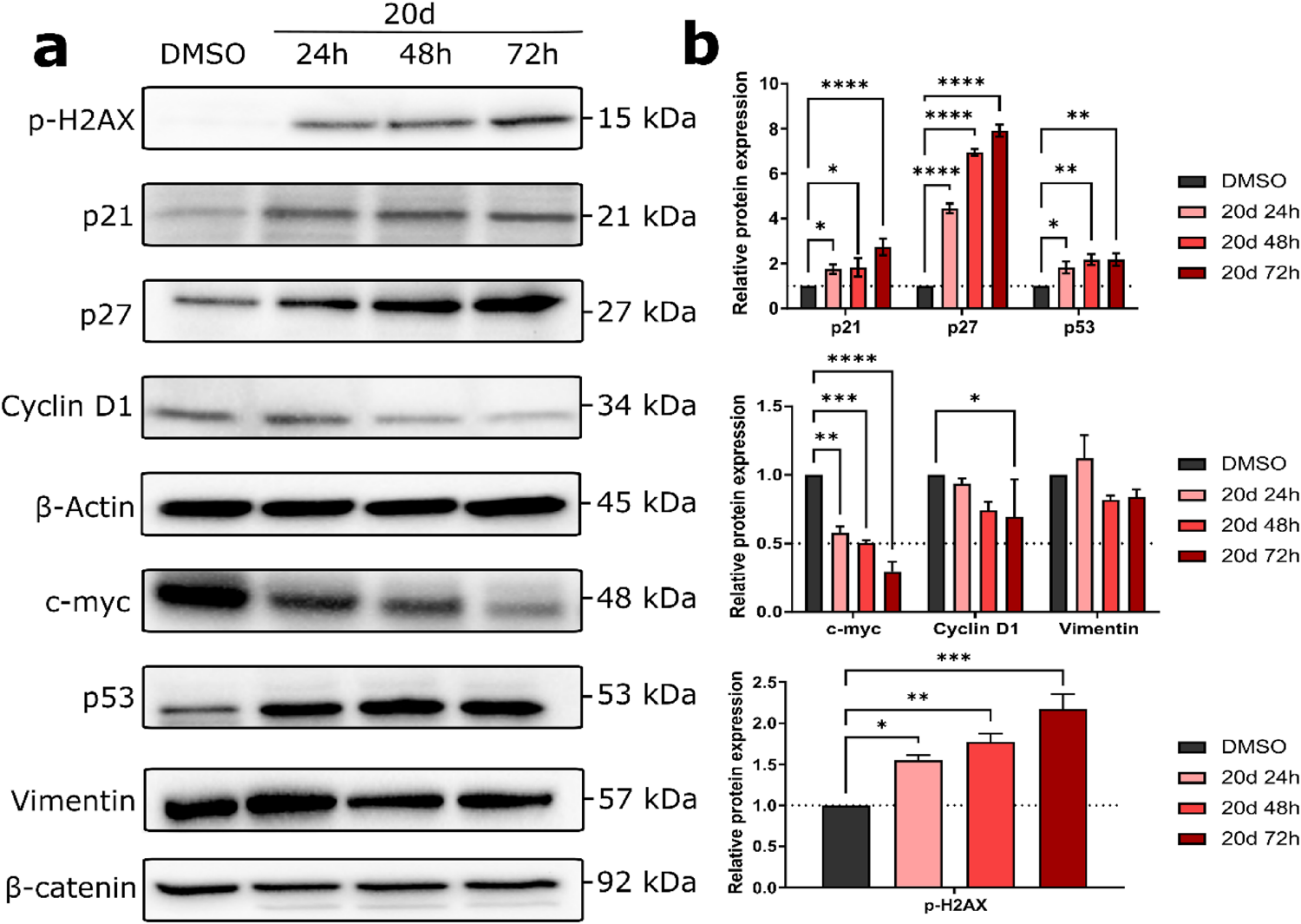
(**a**,**b**) The expression of various proteins detected by Western blot assay after HCT-116 treatment with **20d** or DMSO. The level of each protein was normalized to that of β-actin. Error bars represent the data ± SEM. *p < 0.01, **p < 0.001, ***p < 0.0001, and ****p < 0.00001 vs. vehicle.

To understand the mechanisms underlying **20d**-induced cellular death in detail, we estimated the expression levels of cyclin D1, p21, p27, and p53 in HCT-116 cells by Western blot assay for the indicated time (Fig. 9). p21 is a negative regulator of the cell cycle, which mediates many cellular processes primarily by inhibiting the activity of cyclin-dependent kinase (CDK) 2 and CDK1 (also known as CDC2), leading to growth arrest^43^. p27, another tumor suppressor, halts the cell cycle via CDK complexes, while also playing a role in survival, differentiation, and migration^44^.

As shown in Fig. 9, the expression of p53 and cell cycle regulatory proteins p21 and p27 were markedly higher in **20d**-treated cells (*p* < 0.001) than in the control group. Oxidative stress leads to the stabilization of p53, which is then activated to promote cell cycle arrest and induce autophagy by transcriptionally activating target genes, such as DNA damage-regulated autophagy modulator 1 (*DRAM1*)^45^ and Sestrin 1/2 (*SESN1* and *SESN2*)^46^. While upregulation of proteins p21 and p27 causes halting of G1/S-phase transition, transcription factor p53 upregulates many genes in response to double-strand breaks (DSBs), including proto-oncogene MYC, which codes for the transcription factor c-Myc^47^. Therefore, we analyzed the expression of c-myc after treatment with **20d** and found that it was downregulated in the treated cells as compared to vehicle. This suggests that p53 negatively regulates c-myc. Suppression of MYC may cause tumor cells to lose their neoplastic properties, because of diverting cellular resources toward stress response pathways, permitting cells to recognize DNA damage, leading to enforces their regression and differentiation^47^.

### **20d** induces DNA damage in HCT-116 cells

**Figure 10:**
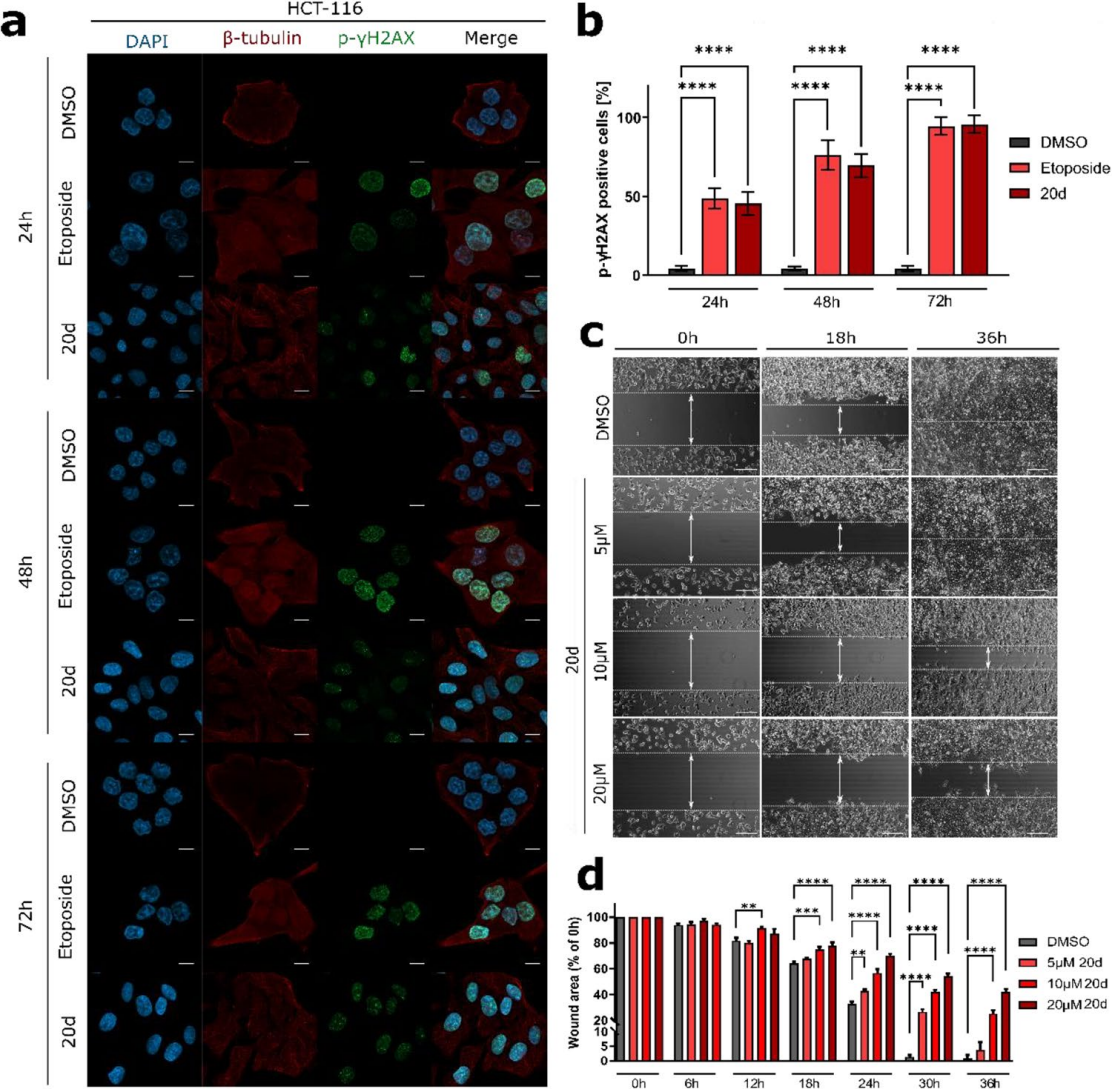
(**a**,**b**) Analyses of the induction of DSBs in HCT-116 cells. Representative microscopic images presenting immunofluorescence. Scale bar = 10 µm. Etoposide and DMSO were used as reference compounds. The microtubule is depicted in red, γ-H2AX in green, and nucleus in blue (DAPI). (**b**) Quantification of the average percent of p-γ-H2AX-positive cells depicted as mean ± SEM of data obtained from 15 random fields of view. (**c**,**d**) The effect of the **20d** compound on the migration ability of HCT-116 cells. (**c**) Representative images captured at 0, 18, and 36 h of treatment of cells with various concentrations of **20d** and DMSO (control). Scale bar = 100 µm (**d**) Quantification of wound healing by measuring the percent of the open scratch area relative to 0 h every 6 h for 36 h. Error bars represent the SEM of data obtained from three independent experiments. **p < 0.001, ***p < 0.0001, and ****p < 0.00001 vs. vehicle.

ROS formation can induce DNA oxidation followed by mutagenic alterations in DNA bases or double-helix breaks, resulting in cell cycle arrest and subsequently cell death^48^. Therefore, we further investigated the effect of **20d** on DNA damage response. HCT-116 cells were incubated with **20d** for 24–72 h, and then the expression of p-γ-H2AX, a known DNA damage marker, was determined by immunoblot and immunofluorescence. As shown in Figs. 9 and 10, even after 24 h, the expression of p-γ-H2AX was found to be significantly increased. It was also noted that **20d** led to this increase in p-γ-H2AX in a time-dependent manner.

β*-*Catenin is a member of the Armadillo repeat protein family and an important component of the cell–cell adhesion machinery. It is also involved in the Wnt growth factor signaling pathway^49,50^. As shown in Fig. 9, **20d** caused the cleavage of β-catenin, even after 24 h of exposure. Proteolysis of β-catenin has often been observed during apoptotic cell death. However, some reports indicate that such proteolysis may also occur due to the disorganization of endothelial adhesion junctions^51–53^. To investigate whether β*-*catenin regulates the expression of cyclin D1 in colon carcinoma cells, we determined its expression after **20d** treatment^54^. We noted a slight inhibition of cyclin D1 expression in **20d**-treated cells compared with the DMSO-treated cells after 72 h of treatment, which might be associated with its induction of growth arrest in colon cancer cells. Additionally, we analyzed the changes in the expression of vimentin, another protein known to be involved in cell–cell junctions and thus contribute to epithelial–mesenchymal transition^55^. As shown in Fig. 9, the expression of vimentin was significantly altered in **20d**-treated cells.

### Suppression of HCT-116 cells migration following **20d** treatment

A decrease in cell–cell adhesion correlates with tumor invasion and metastasis^56^. Migration leads to spreading and metastasis of cancer cells and is thus associated with poor prognosis of many types of cancer^57^. Moreover, studies indicate that metastasis is the major cause of death in patients with colorectal cancer (CRC)^58^. To investigate the effect of **20d** on migration, HCT-116 cells were cultured and investigated by wound healing assay. The percentage of migration was monitored over time by capturing a series of images after every 6 h up to 36 h of treatment. As shown in Fig. 10c,d treatment with **20d** led to a significant reduction (*p* < 0.0001) in migration in HCT-116 cells, compared to DMSO-treated vehicle. This antimigratory effect was more evident at 30 and 36 h of treatment, during which a 30–50% inhibition of migration was observed in HCT-116 cells treated with 10 and 20 µM **20d**. Importantly, we did not observe any detached cells after treatment, which indicates that exposure to **20d** did not cause cell death at any tested time-point. In addition, as shown in Fig. S65 in the supplementary information, the compound **20d** shows a slight cytotoxic effect after 24 h and 48 h exposure at concentrations of 10 and 20 µM, thus observed antimigratory properties of investigated compound is not affected by cell death.

## Discussion

CRC is the most diagnosed cancer in Europe and the second leading cause of cancer-related mortality^59^. The incidence of CRC is likely to increase considerably, mainly due to the aging of the human population and the resistance of CRC to drugs used in conventional chemotherapy^60^. The development of CRC, like other types of cancer, is fostered by the accumulation of genetic and epigenetic alterations, which mainly occurred in tumor suppressor genes, oncogenes, and genes involved in DNA repair mechanisms. Based on the origin of mutation, CRCs are classified as sporadic, inherited, and familial^61^. Moreover, many genomic instability alterations like chromosomal instability (CIN) phenotype, CpG island methylator phenotype (CIMP), and microsatellite instability (MSI) phenotype promote CRC development^62^. The most common CIN occurring in 75% of CRCs is characterized by alterations in chromosome segregation, loss of heterozygosity, abnormalities in the mitotic checkpoint, defective DNA damage response, and others^61^. Chromosome instability involves mutations in tumor suppressor genes and oncogenes, such as Adenomatous Polyposis Coli (APC) in Wnt signaling, and TP53 playing a crucial role in cell cycle arrest and induction of apoptosis. Other examples include KRAS and BRAF providing molecular switches leading to cellular proliferation and growth, and PI3K promoting cell growth and invasion^62,63^.

The PI3K/AKT/mTOR signaling pathway control many cellular processes such as proliferation, growth, motility, apoptosis, autophagy, and metabolism^64^. Therefore, alteration of this pathway is one of the promising therapeutic targets in therapy of primary and metastatic CRCs^65^. The PI3Ks belong to the lipid kinase family and are divided into 3 classes based on their substrate preference and sequence homology. The class I of PI3Ks consists of catalytic (p110α, p110β, p110γ, and p110δ) and regulatory (p85) subunits, and is most frequently mutated in cancers^65^. PI3K inhibitors are subdivided into three categories: (i) isoform-specific inhibitors, e.g. GSK2636771 as an inhibitor of PI3Kβ with anti-tumor activity in PTEN-deficient cancers^66^; (ii) dual PI3K/mTOR inhibitors, such as Dactolisib inhibiting the proliferation and migration of HT-29 human colorectal adenocarcinoma cells^67^; and (iii) pan-PI3K inhibitors, e.g. DHNQ decreasing Colo-205, and HCT-116 colorectal cell proliferation and survival^68^.

Considering the need for novel therapeutic strategies, we synthesized new quinolone derivatives and tested their anticancer properties. Among the synthesized compounds, **20d** and **19b** showed interesting in vitro antiproliferative effects against human colon cancer cell line HCT-116, but no cytotoxicity to nonmalignant embryonic cell line HEK293. Furthermore, the compounds effectively decreased the colony-forming ability of HCT-116 and induced cell cycle arrest at the G1 phase. The PI3K/AKT/mTOR pathway promotes the G1/S transition through transcriptional regulation and control of cyclins and cyclin-dependent kinases like cyclin D1, p21, or p27^68^. Therefore, inhibition of this pathway prevents G1 progression into S, leading to the accumulation of cells in the G1 phase.

Further analyses revealed that **20d** induced massive oxidative stress in colon cancer HCT-116, RKO MMR-proficient and MMR-deficient cells. ROS are involved in several steps in tumorigenesis and play a crucial role in the cellular signaling, regulating cell proliferation and cell survival^69^. However, excess ROS causes an imbalance between intracellular reduction–oxidation processes, causing to damage to cellular components like DNA, proteins, and lipids^30^. Prooxidative compounds are mostly highly selective to ROS-dependent cancer cells without affecting normal cells, representing a notable strategy in the anticancer battle^70^. Many studies report that excessive levels of ROS can induce autophagy through several distinct mechanisms involving Atg4, catalase, the mitochondrial electron transfer chain, and PI3K/AKT/mTOR pathway^71,72^.

Autophagy is controlled by several kinase cascades, among which, the main regulator is the mammalian target of rapamycin, complex 1 (mTORC1) kinase. This kinase is involved in each step of the autophagy process, including nucleation, autophagosome elongation, autophagosome maturation and termination^73^**.** Inhibition of mTORC1 suppresses the autophagy-initiating UNC-5 like autophagy activating kinase (ULK) and PI3K, followed by the initiation of the formation of a phagophore^41^. In the present study, **20d** was found to induce autophagic cell death in HCT-116 cells. A variety of autophagic markers related to the formation and maturation of autophagosomes and phagosomes like LC3-II and LAMP1 were observed. Moreover, **20d** suppressed the activity of mTOR and diminished the phosphorylation of AKT and the level of c-Myc*,* followed by decreasing the expression of several key proteins involved in the regulation of the cell cycle. Degtyarev et al. reported that knockdown or inactivation of AKT leads to markedly increased autophagy rather than apoptosis and interestingly this phenomenon was related to marked accumulation of ROS^18^. Indeed, we did not observe any typical features of apoptosis-like cell shrinkage, membrane blebbing, chromatin condensation, or loss of plasma membrane phosphatidylserine asymmetry. However, inhibition of the PI3K/AKT/mTOR pathway could promote cancer cell death via different mechanisms. Wen et al. reported that chaetocin, a natural product isolated from the *Chaetomium,* inhibited the proliferation of gastric cancer in cell xenografts and patient-derived xenografts through inactivation of the PI3K/AKT pathway by inducing ROS leading to both apoptotic and autophagy cell death^41^. Other reports showed that PI3K inhibitor LY294002 and the mTOR inhibitor rapamycin promoted the expression of autophagy-related proteins via inhibition of the PI3K/AKT/mTOR signaling pathway. Furthermore, it was found that **20d** suppressed the cell migration in HCT116 cells in a time-dependent manner. Indeed, inhibition of the PI3K/AKT/mTOR pathway has also been found to suppress migration in other tumors including breast cancer^74^, bile duct cancer^75^, and osteosarcoma^76^. These findings indicate that **20d** is a potential tumor suppressor in colon cancer.

In summary, we presented new quinolone derivatives with promising anticancer properties against CRCs. The most potent compound **20d** induced massive oxidative stress leading to autophagy by interfering with the PI3K/AKT/mTOR signaling pathway. The compound also caused deregulation of several proteins involved in cell cycle progression and maintenance and showed an anti-migratory effect on HCT-116 cells. Overall, the study provides convincing experimental evidence for the application of novel quinolone derivatives as compounds inhibiting cancer cell growth and metastasis. Thus, it can be concluded that **20d** could be used as a lead compound for drug discovery based on quinolone derivatives.

### Methods and experimental

General: Commercially available reagents were purchased from Sigma-Aldrich or Acros and used without further purification. DCM was distilled over P_4_O_10_ and stored over molecular sieves. The reagents were prepared as described in the literature. Analytical thin-layer chromatography was performed on aluminum sheets of UV-254 Merck silica gel, and flash chromatography using SilicaFlash P60 silica gel (40–63 µm). ^1^H and ^13^C NMR spectra were recorded with Bruker Avance III HD 400 MHz, and NMR chemical shifts were reported in δ (ppm) using residual solvent peaks as standards with the coupling constant J measured in Hz. High-resolution mass spectra were recorded with an Agilent 6540 Q-TOF system. Melting points were determined with a Warsztat Elektromechaniczny W-wa apparatus and were used uncorrected. All experimental data and spectra are collected in Supplementary Information.

### Docking study

Molecular docking of the designed molecules was performed using AutoDock Vina^20^ and AutoDock4.2 software packages^21,22,77^. The structures of kinases were deduced from PDB. We used the crystallographic structures of human γ^23^ and mouse δ^24^ PI3K for our analysis. In the case of crystallographic structures containing complexed ligand, the atoms of the ligand were selectively removed from the crystallographic structures of the proteins if necessary. The energy of the ligand molecules was minimized using an MM2 force field. During docking, the ligands had torsion allowed for all rotating dihedral angles.

### Cell culturing

A-549 (CCL-185) and MCF-7 (HB-8065) cells were cultured in RPMI-1640 medium, while HCT-116 (CVCL-427), (hMLH1)-deficient (MMR^–^) and hMLH1-proficient (MMR^+^) RKO cells were cultured in McCoy’s 5A medium. The culture media were supplemented with 10% fetal bovine serum, 2 mM l-glutamine, and antibiotics (penicillin 62.6 µg/ml and streptomycin 40 µg/ml). Cultivation of cell lines was carried out at 37 °C under a humidified atmosphere containing 5% CO_2_, and the cultures were routinely screened for *Mycoplasma* contamination. A-549, MCF-7, and HCT-116 cells were obtained from American Type Culture Collection (ATCC), while RKO colon carcinoma cells were a kind gift of Dr Timothy Kinsella (Brown University, USA).

### Cell viability assay

The viability of cells was determined in response to the newly synthesized compounds using the MTT (Sigma-Aldrich) assay. Briefly, cells were seeded into 96-well plates and allowed to attach overnight. Then, cells were exposed to various concentrations of compounds for 24, 48 or 72 h. Negative controls were treated with the same amount of DMSO solvent (1%). After treatment, the MTT solution (0.4 mg/ml) was added to each well and allowed to react with cells for 2–3 h at 37 °C. Next, the medium was removed, the formazan product formed was dissolved in 100 μl of DMSO, and the absorbance was determined at 540 nm using an ASYS UVM340 microplate reader (Biochrom Ltd.). The IC_50_ value, which represents the concentration of the compound required to inhibit cell growth by 50% compared to vehicle, was calculated using GraphPad Prism 8 software based on the curves plotting survival against dose averaged from at least three independent experiments.

### Colony formation assay

A total of 500 HCT-116 cells/well was seeded into six-well plates. After attachment, cells were pretreated with DMSO (1%) or various concentrations of **20d** and **19b** for 24 h. Following incubation, the medium was changed, and cells were cultured for 9 days. After culturing, cells were washed with phosphate-buffered saline (PBS), fixed with 100% methanol for 30 min, and then stained with 0.5% crystal violet for 15 min. The plates were air-dried, and visible colonies were counted using ImageJ software. The percentage viability of cells was calculated in comparison to the control.

### Cell cycle distribution analysis

HCT-116 and A-549 cells were seeded into culture plates and allowed to attach overnight. Then, cells were incubated with the investigated compounds for the indicated time points. After incubation, the harvested cells were fixed with ice-cold 75% ethanol and stored overnight at − 20 °C. Next, cells were rehydrated with PBS in ice for 15 min and washed twice in PBS. Finally, cells were stained with 20 μg/μl propidium iodide (PI) and 50 μg/μl RNase A in PBS for 30 min at room temperature. Cell cycle distribution was determined using a Guava easyCyte 8 flow cytometer (Merck Millipore) and FlowJo v10 software.

### Annexin V-FITC staining

To determine the proapoptotic activity of compounds, cells were seeded into Petri dishes. After overnight attachment, cells were treated with IC_50_ concentrations of **20d** and **19b** for 6, 24, and 48 h. DMSO (1%, Merck) and Etoposide (10 μM, Sigma-Aldrich) were used as references. Cells were harvested and stained with FITC-Annexin V, (Thermo Fisher, V13242) according to the manufacturer’s instructions. Finally, cells were stained with propidium iodide (Thermo Fisher) and assessed using a Guava easyCyte 8 cell sorter (Merck Millipore). The results were analyzed using FlowJo v10 software.

### Measurement of extracellular ROS activity

The extracellular ROS levels were determined using an H2DCFDA (2′,7′-dichlorodihydrofluorescein diacetate) probe (Thermo Fisher) following the manufacturer’s protocol. Briefly, HCT-116 and A-549 cells were treated with IC_50_ concentrations of **20d** and **19b** for 1, 3, 6, and 24 h. DMSO (1%, Merck) and 250 μM H_2_O_2_ were used as a negative and positive control, respectively. NAC (500 μM, Sigma-Aldrich) was used as an ROS scavenger to confirm the ROS activity. After treatment with **20d** and **19b**, cells were incubated with H2DCFDA at 37 °C for 30 min. Subsequently, cells were harvested by trypsinization, rinsed with PBS, and stained with 7-AAD (Thermo Fisher). Finally, the stained cells were analyzed by flow cytometry using a Guava easyCyte 8 cell sorter (Merck Millipore) and FlowJo v10 software.

### Confocal live cell imaging

ROS analysis was performed by seeding the cells into cover glass-bottom 24-well plates for live-cell imaging. Cells were treated with compounds and stained as described above for flow cytometry. Images were acquired with an LSM 800 inverted laser-scanning confocal microscope (Carl Zeiss), equipped with an airyscan detector for high-resolution confocal scanning, using a ×63 1.4-NA Plan Apochromat objective (Carl Zeiss). The incubation chamber was maintained at 37 °C with 5% CO_2_ throughout the analysis.

### Western blot

HCT-116 cells were seeded into 60-mm Petri dishes at a density of 1.5 × 10^6^ cells per dish and incubated overnight. Then, cells were treated with IC_50_ concentration of **20d** or 1% DMSO for 24, 48, and 72 h. After treatment, cells were lysed in Laemmli buffer with a protease and phosphatase inhibitors cocktail (Roche), followed by sonification The lysate was cleared by centrifuging at 16,000×*g* for 15 min at 12 °C. The supernatant was collected, and the extract was quantified for protein using the DC Protein Assay (Bio-Rad). Proteins were resolved by 10% or 12% sodium dodecyl sulfate–polyacrylamide gel electrophoresis and transferred to polyvinylidene difluoride membranes (Bio-Rad). Membranes were blocked using 5% (w/v) bovine serum albumin (Sigma-Aldrich) in 1× TBST (pH 7.4 and 0.1% Tween-20) for 1 h at room temperature and subsequently incubated overnight with primary antibodies at 4 °C. The blots were washed thrice with TBST and then incubated with horseradish peroxidase-conjugated antibodies for 1 h at room temperature. After washing, the membranes were evaluated for detecting the protein bands using enhanced chemiluminescence detection reagent kit (Thermo Fisher) and ChemiDoc XRS + Imaging System (Bio-Rad). The band intensity was measured using ImageLab 5.2 software (Bio-Rad). All the used antibodies are listed in Table 2.

**Table 2:** List of antibodies used in Western Blot.

Antibody name	Company	Dilution
Anti-mTOR, #2983	Cell signaling	1:1000
Anti-p(Ser2448) mTOR, #5536	Cell signaling	1:1000
Anti-LAMP1 (D4O1S), #15665	Cell signaling	1:1000
Anti- LC3B, #2775	Cell signaling	1:1000
Anti-AKT, #4691	Cell signaling	1:1000
Anti-pAKT, #9271	Cell signaling	1:1000
Anti-c-myc, #5605	Cell signaling	1:1000
Anti-p-H2AX	Cell signaling	1:100
Anti-vimentin, #5741	Cell signaling	1:1000
Anti-actin, sc-1616	Santa Cruz	1:100
Anti-β-catenin, sc-133240	Santa Cruz	1:250
Anti-cyclin D1, sc-246	Santa Cruz	1:100
Anti-p21, sc-397	Santa Cruz	1:250
Anti-p27, sc-1641	Santa Cruz	1:250
Anti-p53, sc-126	Santa Cruz	1:250
Anti-mouse-HRP, 715-035-150	Jacson ImmunoResearch Labs	1:10,000
Anti-rabbit-HRP, 711-035-152	Jacson ImmunoResearch Labs	1:10,000
Anti-goat-HRP, 705-036-147	Jacson ImmunoResearch Labs	1:10,000

### Wound healing assay

The antimigratory properties of **20d** were investigated in HCT-116 cell line using wound healing assay. Briefly, cells were seeded and allowed to attach to the Ibidi-silicone insert on a cover glass-bottom 24-well plate for live-cell imaging. Next, the inserts were dislodged, and subsequently, cells were washed with a fresh medium and incubated with various concentrations of **20d** in an imaging chamber (cellVivo incubation system, Olympus). For monitoring the migration of cells by live-cell microscopy with time-lapse photography, images were captured every 15 min for 36 h under 10× magnification using a fluorescence microscope (IX83 Inverted Microscope, Olympus) equipped with an XC50 phase-contrast digital color camera (Olympus). The distance between gaps was measured using ImageJ software.

### Statistical analyses

Statistical analysis was performed using GraphPad Prism 8 software. Unless stated otherwise, statistical significance was determined using one-way analysis of variance (ANOVA) with post hoc Dunnett’s test. A probability value of < 0.05 was considered statistically significant.

## Supplementary Information

Supplementary Information.

## Data Availability

The datasets presented in the current study are available from the corresponding author on reasonable request.
