# Supplementary material for: Design, synthesis, and biological evaluation of tetrahydroquinolinones and tetrahydroquinolines with anticancer activity
Source: Sci Rep. 2022 Jun 15;12:9985. doi: 10.1038/s41598-022-13867-x (PMC9200803; doi:10.1038/s41598-022-13867-x)
Supplement: Supplementary file 1 — Supplementary Information. [file 41598_2022_13867_MOESM1_ESM.docx]

**SUPPLEMENTARY INFORMATION**

**Design, synthesis, and biological evaluation of tetrahydroquinolinones and tetrahydroquinolines with anticancer activity**

**Małgorzata Ryczkowska^1^‡, Natalia Maciejewska^2^‡, Mateusz Olszewski^2^, Milena Witkowska^1^, and Sławomir Makowiec^1^***

^1^ Department of Organic Chemistry, Faculty of Chemistry, Gdansk University of Technology, Narutowicza 11/12, 80-233, Gdansk, Poland.

^2^ Department of Pharmaceutical Technology and Biochemistry, Faculty of Chemistry, Gdansk University of Technology, Narutowicza 11/12, 80-233, Gdansk, Poland.*mak@pg.edu.pl

**EXPERIMENTAL DATA**

**1. 3-oxo-3-phenyl-propanamide [1]**

Ethyl benzoylacetate (3.6 ml, 21 mmol) and 50 ml of 24% amonnia solution (amonnia water) were placed in round-bottomed flask with stir bar. EtOH was added dropwise unless clear solution was obtained. The reaction temperature was rised up to 45^o^C and kept for 16h. After cooling to RT water was evporated azeotropically with toluene. The residue was dried under preasure. Raw 3-oxo-3-phenyl-propanamide (2.4 g, 12.5 mmol, 59%) was obtained as yellow powder and used in the next step without farther purification.

White amorphous powder, yield: 59%,


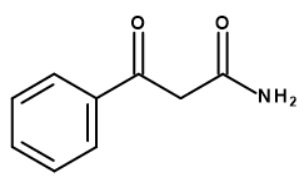
^1^H NMR (CDCl_3_, 400 MHz): δ= 7.98 (d, *J* = 7.5 Hz, 2 H), 7.61 (t, *J* = 7.7 Hz, 1 H), 7.50 – 7.46 (m, 2 H), 7.15 (s, 1 H), 6.03 (s, 1 H), 3.97 (s, 2 H)

^13^C NMR (CDCl_3_, 100 MHz): δ= 195.69, 168.55, 136.2, 134.25, 129.01, 128.68, 45.25

HRMS (ESI+): *m*/*z*

[M + H]+ calcd for C9H10NO2: 164.0712 ; found: 164.0712.

**
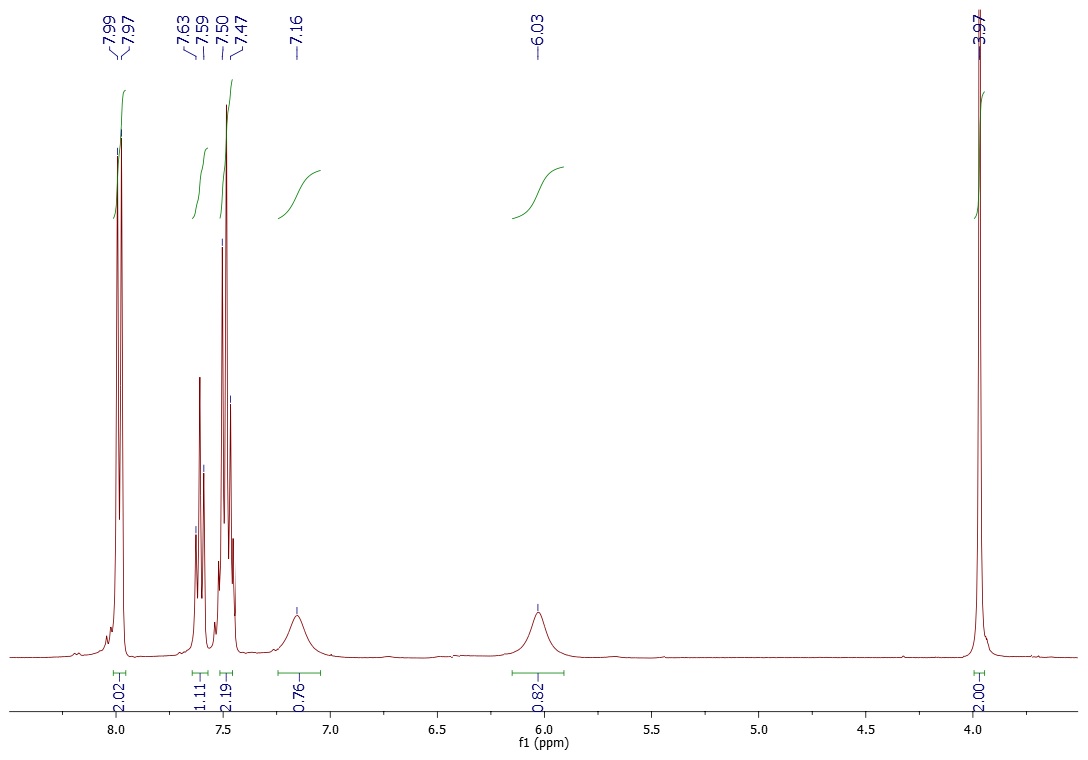
**

**Figure S1. ^1^H NMR of 3-oxo-3-phenyl-propanamide**


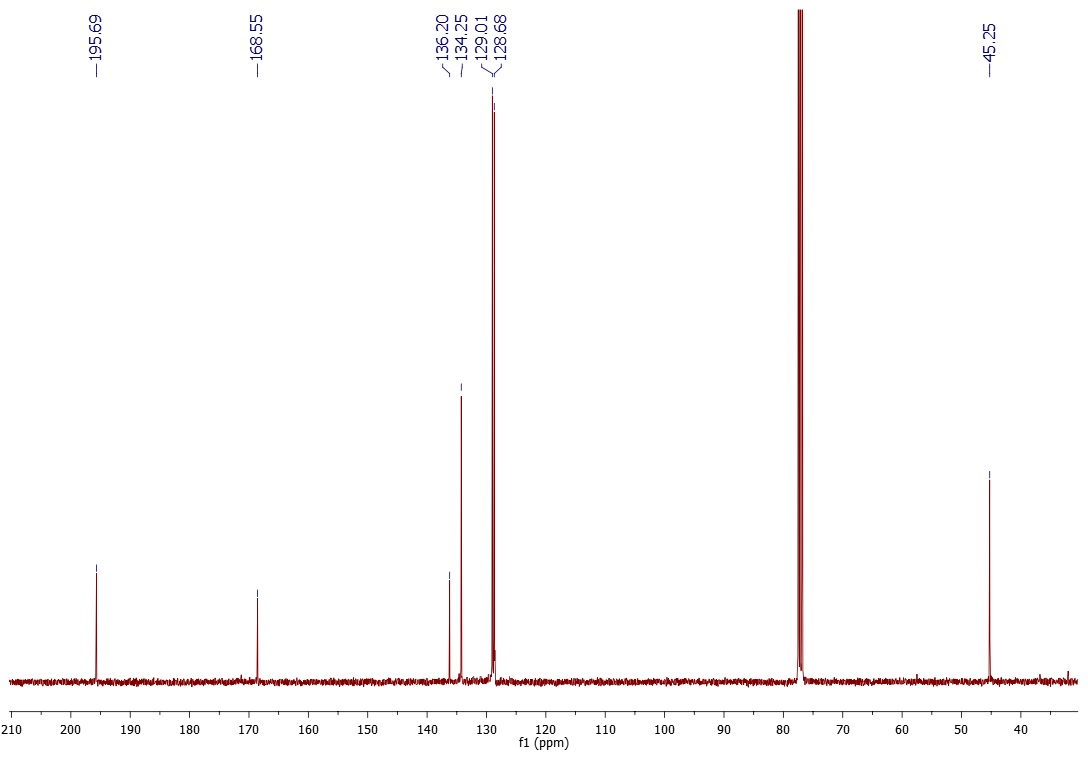


**Figure S2. ^13^C NMR of 3-oxo-3-phenyl-propanamide**

**2. 4-phenyl-5,6,7,8-tetrahydro-1H-quinolin-2-one (9) [1]**

A mixture of 3-oxo-3-phenyl-propanamide (0.1 g, 0.61 mmol), cyklohexanone (0.064 ml, 0.61 mmol), TsOH (0.232 g, 1.22 mmol) and 2.5 ml of toluene was placed in round-bottomed flask with stir bar. Reaction mixture was heated to reflux and left overnight. The solvent was evaporated. Residue was dissolved in DCM and washed with water, 2M NaOH and brine. Organic layer was dried with anhydrous MgSO_4._ The crude product was isolated by flash column chromatography (dichloromethane/methanol 60:1). The title compound (0.115 g, 0.51 mmol, 83%) was obtained as a white powder.


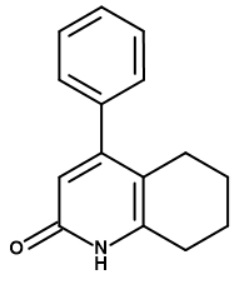
White powder, yield: 83%, decomposition: around 242^o^C

Mixture of tautomers 3:2

^1^H NMR (CDCl_3_, 400 MHz): δ= 7.43 – 7.37 (m, 3 H_Ar_ , NH), 7.30 – 7.26 (m, 3 H), 6.42 (s, 1 H), 5.30 (s, OH), 2.82 (t, *J* = 5.9 Hz, 2 H), 2.32 (s, 2 H), 1.85 – 1.79 (m, 2 H), 1.7 – 1.64 (m, 2 H)

^13^C NMR (CDCl_3_, 100 MHz): δ= 157.33, 143.76, 138.85, 128.41, 128.25, 128.11, 116.65, 114.35, 27.47, 25.72, 22.96, 21.73

HRMS (ESI+): *m*/*z*

[M + H]+ calcd for C15H16NO: 226.1232 ; found: 226.1249.

**
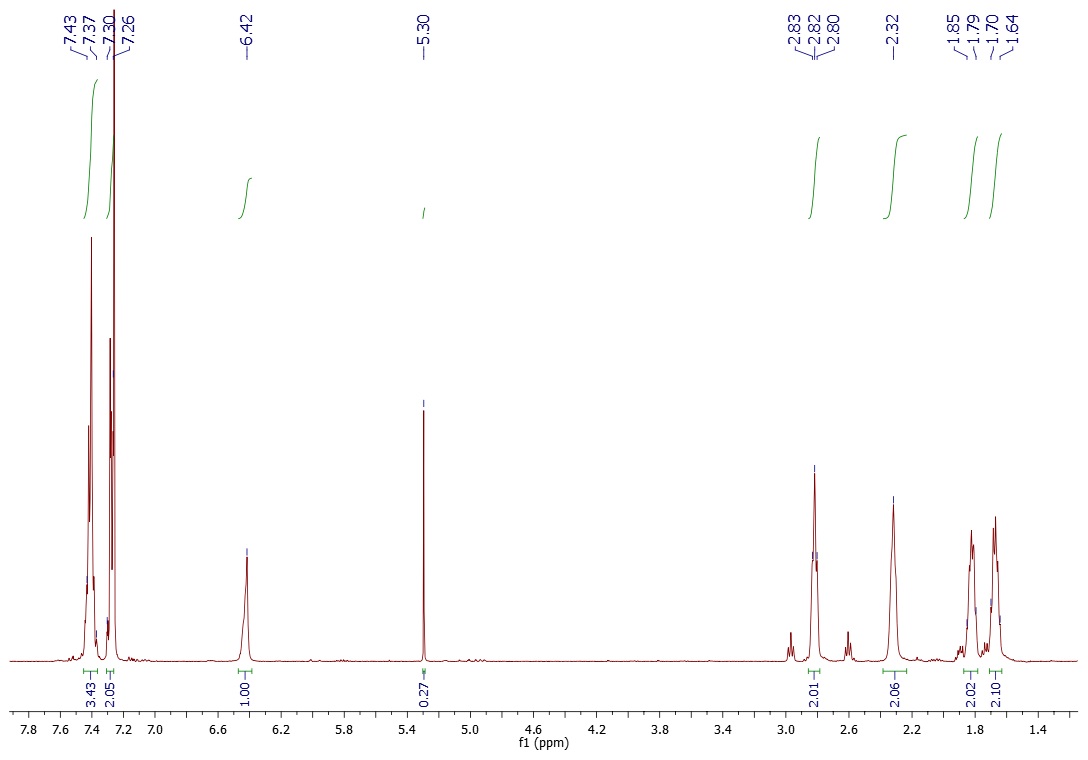
**

**
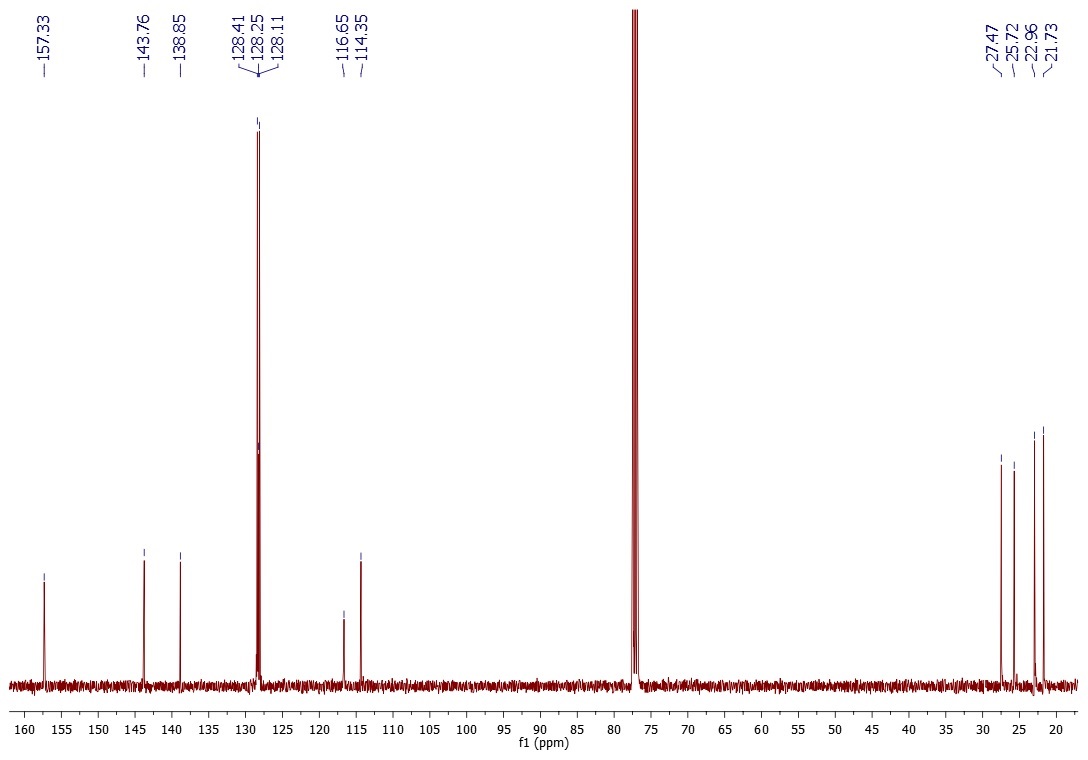
Figure S3. ^1^H NMR of 4-phenyl-5,6,7,8-tetrahydro-1H-quinolin-2-one**

**Figure S4. ^13^C NMR of 4-phenyl-5,6,7,8-tetrahydro-1H-quinolin-2-one**

**3. 2-chloro-4-phenyl-5,6,7,8-tetrahydroquinoline (10) [2]**

**2** (1.46 g, 6.67 mmol) and 6 ml of PhPOCl_2_ were placed in round-bottomed flask with stir bar. The reaction temperature was raised up to 160^O^C and left overnight. Post-reaction mixture was gently diluted with water and washed with DCM. Organic layer was dried with anhydrous MgSO_4_. After solvent evaporation the crude product was isolated by flash column chromatography (ethyl acetate : hexane 1:30). The title compound (1.41 g, 5.75 mmol, 87%) was obtained as white cristals.

White cristals, yield: 87%, mp: 83-85^o^C


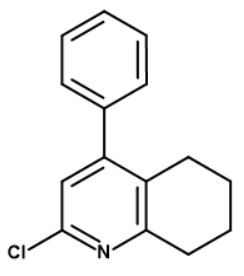
^1^H NMR (CDCl_3_, 400 MHz): δ= 7.46 – 7.38 (m, 3 H), 7.28 – 7.26 (m, 2 H), 7.02 (s, 1 H), 2.98 (t, *J* = 6.6 Hz, 2 H), 2.58 (t, *J* = 6.2 Hz, 2 H), 1.89 (qu, *J* = 6.1 Hz, 2 H), 1.72 (qu, *J* = 6.1 Hz, 2 H)

^13^C NMR (CDCl_3_, 100 MHz): δ= 158.64, 152.92, 147.79, 138.23, 129.11, 128.58, 128.45, 128.37, 122.02, 32.9, 27.17, 22.81

HRMS (ESI+): m/z

[M + H]+ calcd for C15H15ClN: 244.0893 ; found: 244.0927.

**
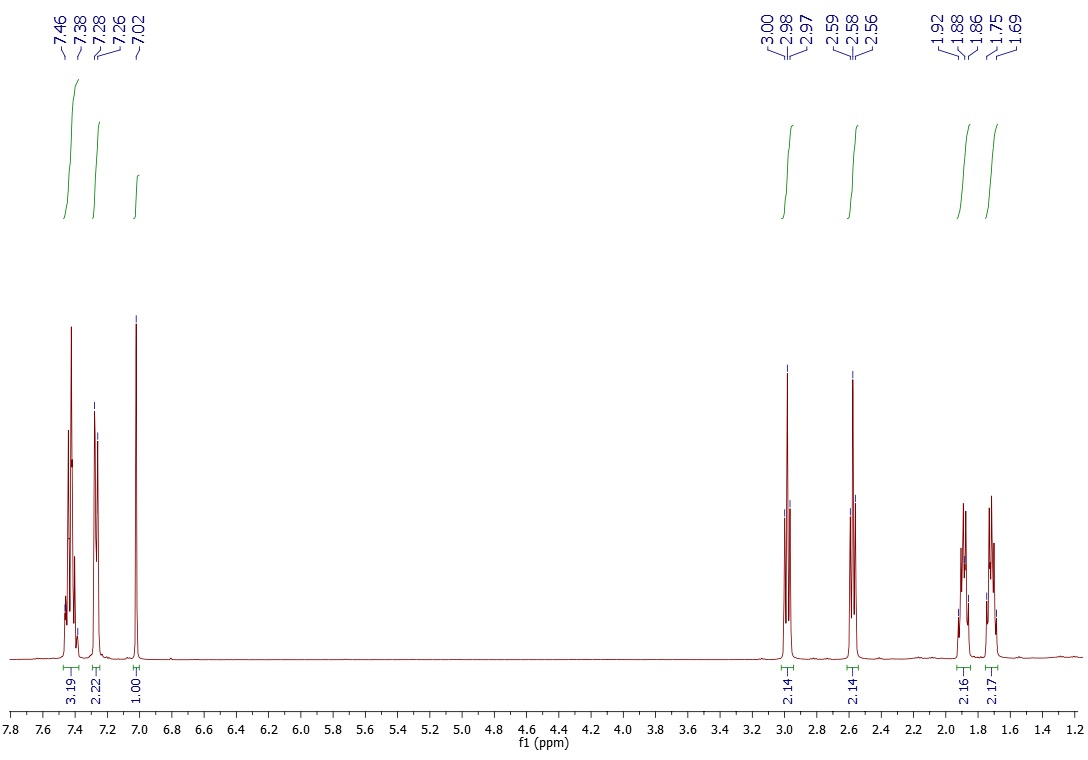
**

**Figure S5. ^1^H NMR of 2-chloro-4-phenyl-5,6,7,8-tetrahydroquinoline**

**
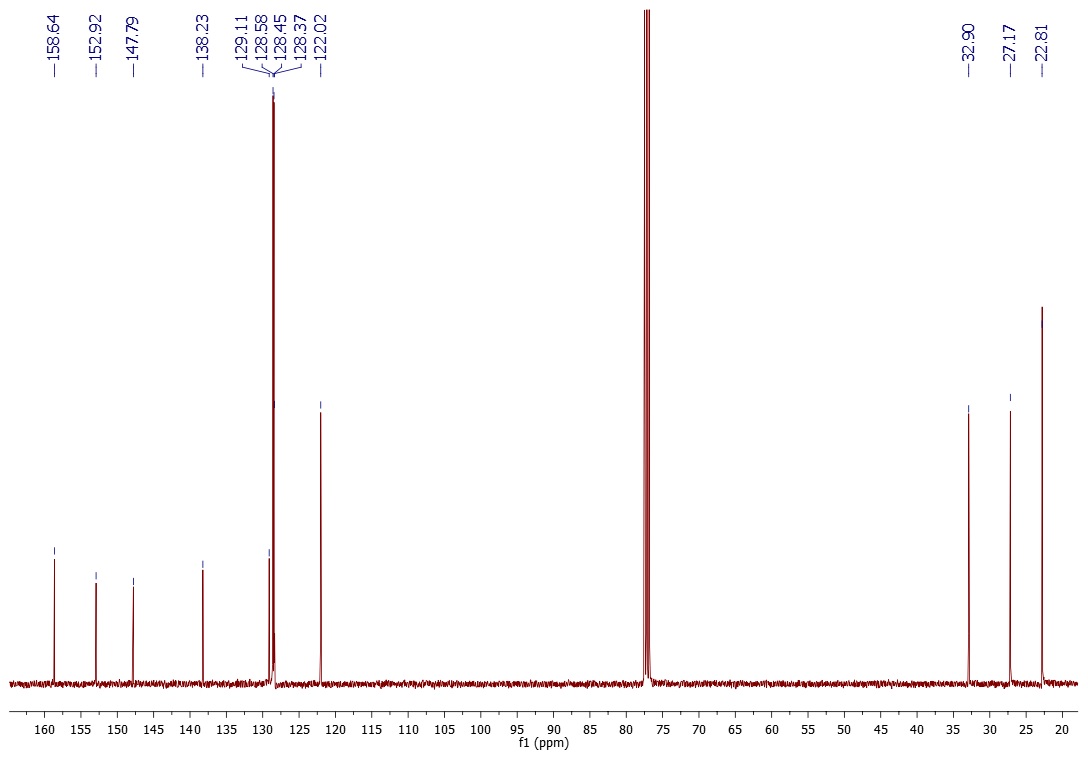
**

**Figure S6. ^13^C NMR of 2-chloro-4-phenyl-5,6,7,8-tetrahydroquinolin**

**4. 2-methoxy-4-phenyl-5,6,7,8-tetrahydroquinoline (11) [3]**

A solution of **2** (0.06 g, 0.27 mmol) in 2 ml of CHCl_3_ was placed in round-bottomed flask with stir bar. Ag2CO3 (0.042 g, 0,15 mmmol) and MeI (0.079 ml, 1.1 mmol) were added. The mixture was left overnight in RT. Ag_2_CO_3_ was filltred on celite and CHCl_3_ was evaporated. Crude product was purified by flash column chromatography (ethyl acetate : hexane 1:4). Compound **11** (0.019 g, 0.075 mmol, 68%) was obtained as white cristals

White amorphous powder, yield: 85%


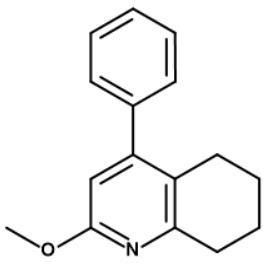
^1^H NMR (CDCl_3_, 400 MHz): δ= 7.44 – 7.35 (m, 3 H), 7.29 – 7.27 (m, 2 H), 6.46 (s, 3 H), 2.9 (t, *J* = 6.4 Hz, 2 H), 2.49 (t, *J* = 6.2, 2 H), 1.89 – 1.83 (m, 2 H), 1.73 – 1.67 (m, 2 H)

^13^C NMR (CDCl_3_, 100 MHz): δ= 161.66, 154.82, 153.10, 139.72, 128.56, 128.35, 127.82, 122.72, 108.22, 53.57, 32.93, 26.90, 23.35, 23.16

HRMS (ESI+): m/z

[M + H]+ calcd for C16H18NO: 240.1388 ; found: 240.1410

**
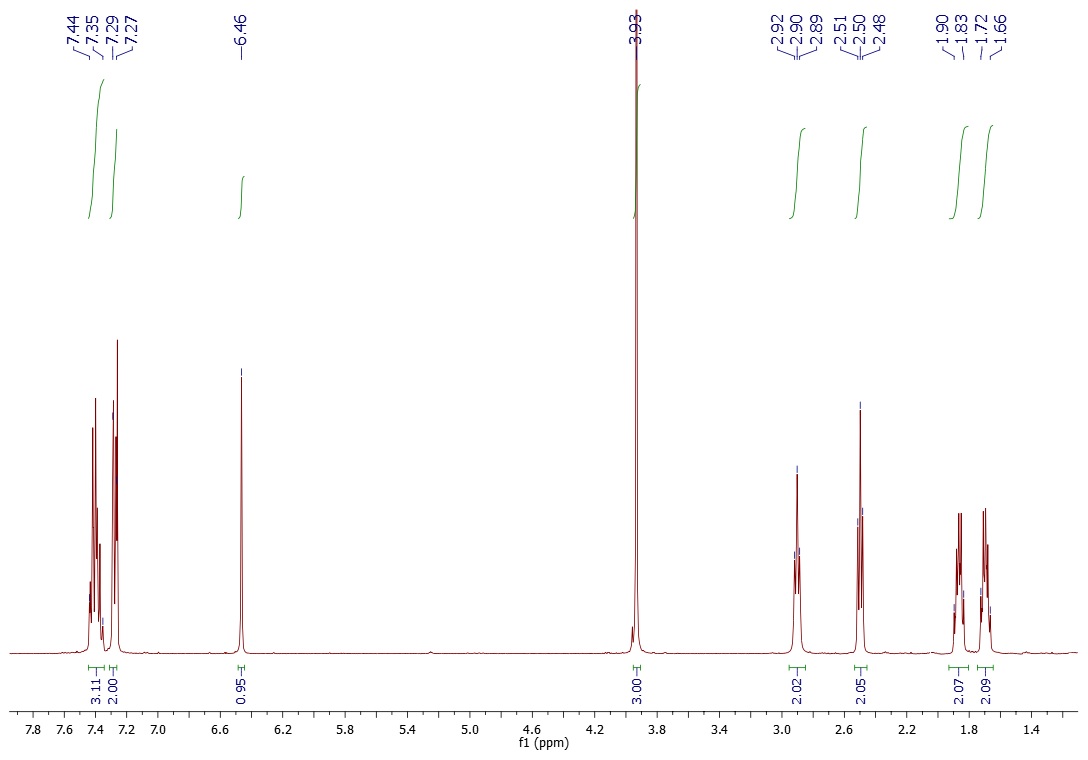
**

**Figure S7. ^1^H NMR of 2-methoxy-4-phenyl-5,6,7,8-tetrahydroquinoline**

**
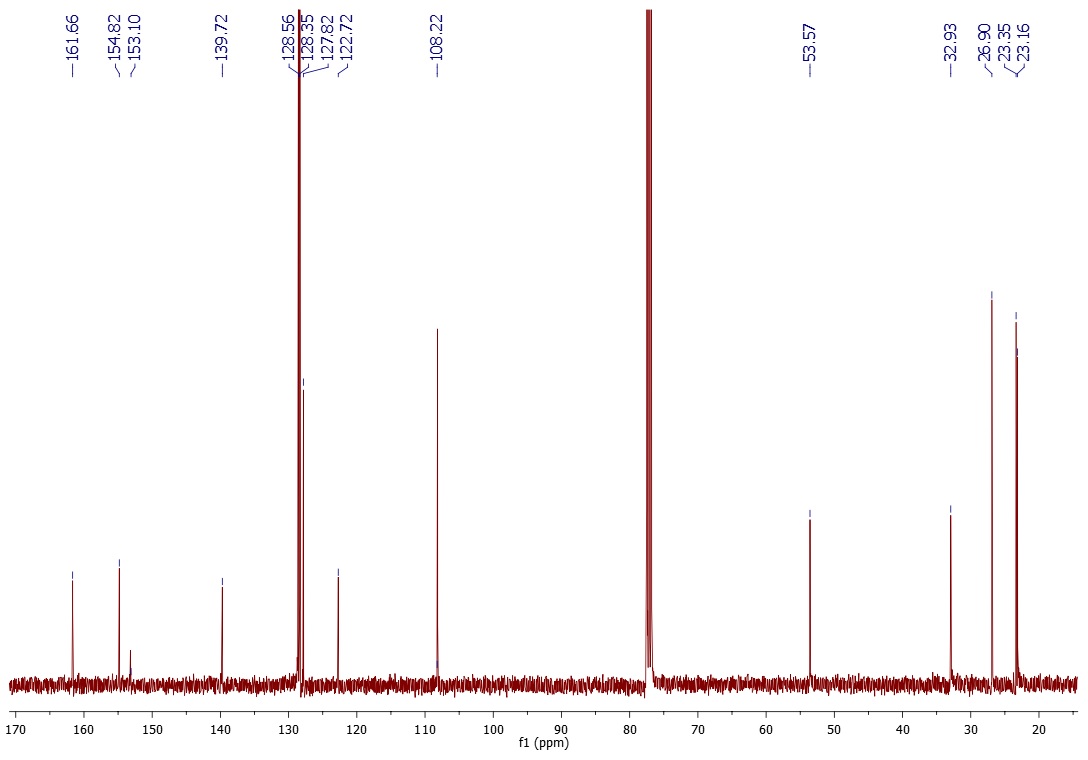
**

**Figure S8. ^13^C NMR of 2-methoxy-4-phenyl-5,6,7,8-tetrahydroquinoline**

**5. (2-chloro-4-phenyl-5,6,7,8-tetrahydroquinolin-8-yl) acetate (14) [2]**

The mixture of **10** (0.093 g, 0.38mmol), 30% hydrogen peroxide (0.182 ml, 1.61 mmol) and acetic acid (0.7 ml, 12 mmol) was placed in round-bottomed flask with stir bar. The reaction temperature was heated to 80^O^C and left overnight. After solvent evaporation the residue was diluted with water and neutralized with K_2_CO_3_. Pyridine N-oxide **12** was extracted with DCM and dried with anhydrous MgSO4. After solvent evaporation 0,078 g of pyridine N-oxide was obtained as yellow amorphous powder. Without farther purification pyridine N-oxide was placed in round-bottomed flask and 4 ml of acetic anhydride was added. Reaction mixture was left in RT for 1 h and then heated to 100^o^C for next 4h. After cooling and solvent evaporation crude product was isolated by flash column chromatography (ethyl acetate : hexane 1:10). The title compound (0.073 g, 0.24 mmol, 64%) was obtained as bright yellow powder.


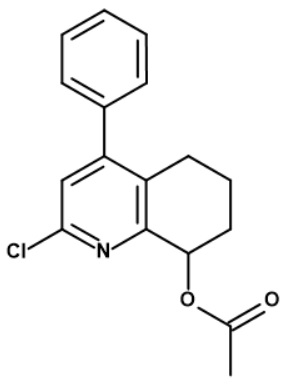
Bright yellow amorphous powder, yield: 64%

^1^H NMR (CDCl_3_, 400 MHz): δ= 7.48 – 7.42 (m, 3 H), 7.28 – 7.25 (m, 2 H), 7.15 (s, 1 H), 5.93 (t, *J* = 4.6 Hz, 1 H), 2.66 (dt, *J^2^* = 17.4 Hz, *J^3^* = 5.0 Hz, 1 H), 2.55 (ddd, *J^2^* = 17.3 Hz, *J^3^* = 9.1 Hz, *J^3^* = 5.8 Hz, 1 H), 2.21 – 2.15 (m, 1 H), 2.13 (s, 3 H), 2.07 – 1.99 (m, 1 H), 1.87 – 1.74 (m, 1 H)

^13^C NMR (CDCl_3_, 100 MHz): δ= 170.34, 154.16, 153.36, 148.97, 137.69, 130.72, 128.73, 128.67, 128.41, 124.52, 71.10, 28.78, 26.95, 21.60, 18.48

HRMS (ESI+): m/z

[M + H]+ calcd for C17H17ClNO2: 302.0948 ; found: 302.0970.

**
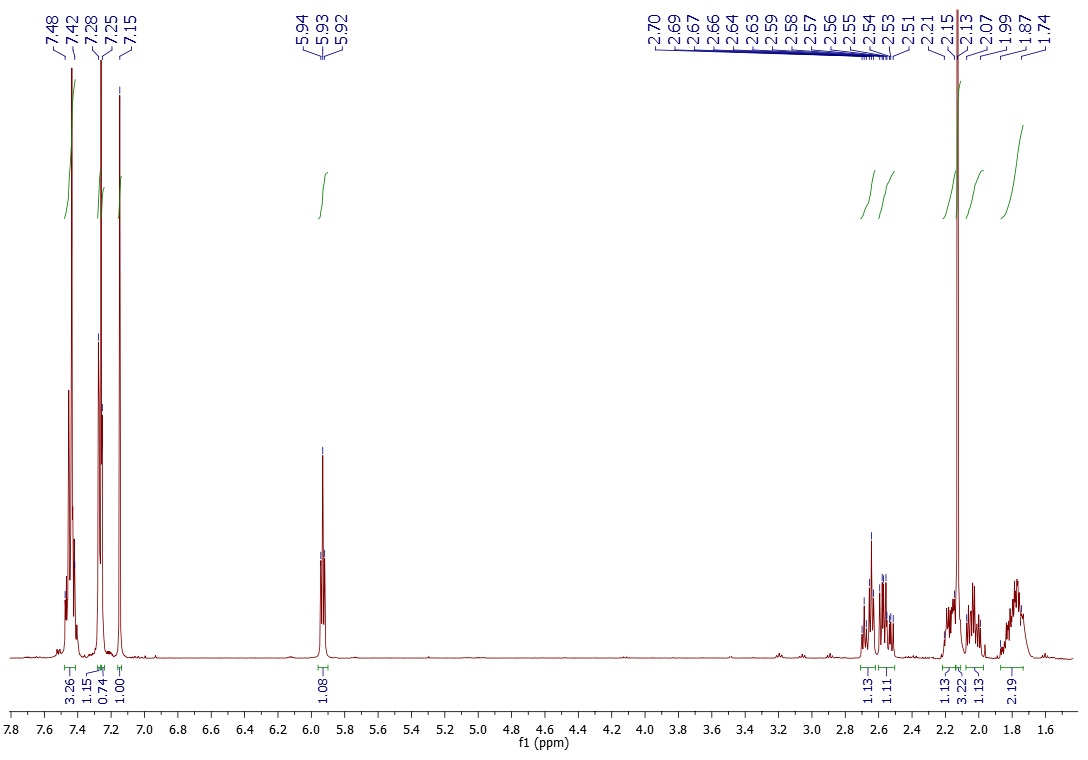
**

**Figure S9. ^1^H NMR of (2-chloro-4-phenyl-5,6,7,8-tetrahydroquinolin-8-yl) acetate**

**
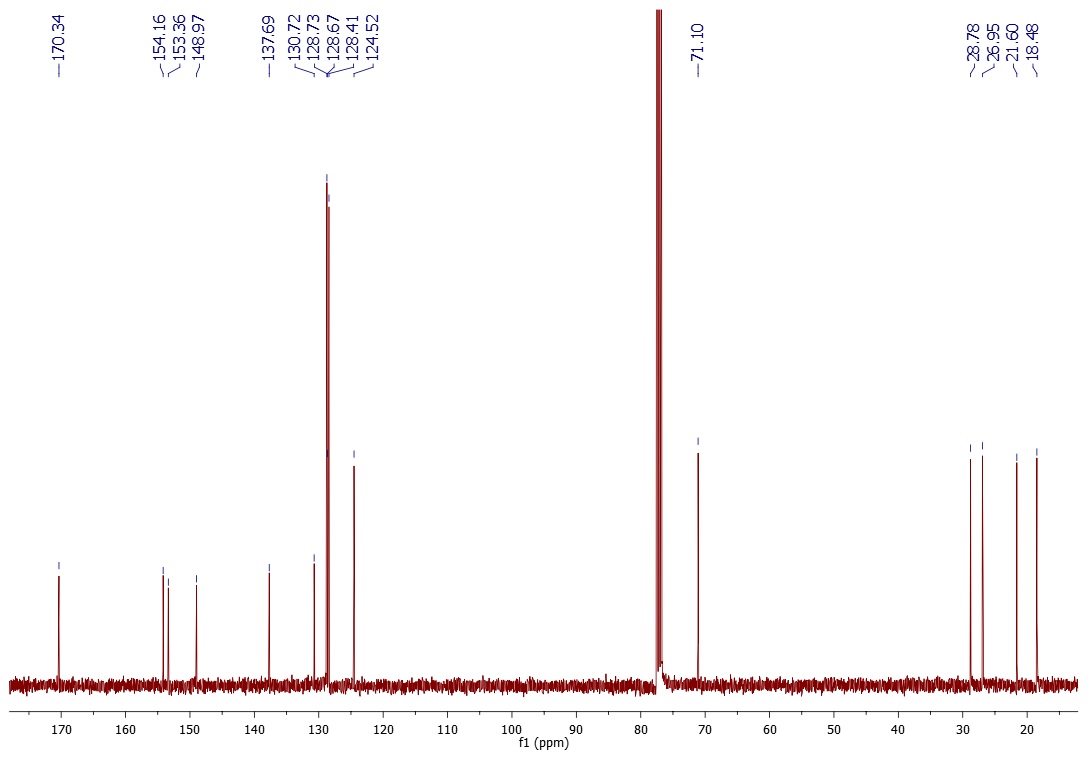
**

**Figure S10. ^13^C NMR of (2-chloro-4-phenyl-5,6,7,8-tetrahydroquinolin-8-yl) acetate**

**6. (2-methoxy-4-phenyl-5,6,7,8-tetrahydroquinolin-8-yl) acetate (15)**

The title compound was synthesised according to procedure of **14**. **15** (0.263, 0.88 mmol, 51%) was obtained as colorless oil.


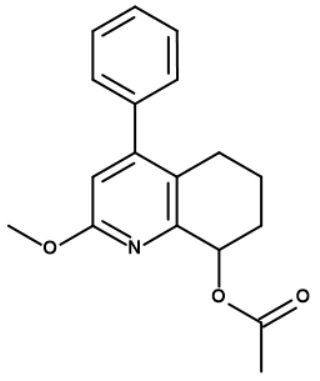
Colorless oil, yield: 51%

^1^H NMR (CDCl_3_, 400 MHz): δ= 7.44 – 7.38 (m, 3 H), 7.27 – 7.25 (m, 2 H), 6.58 (s, 1 H), 5.99 (t, *J^3^* = 5.3, 1 H), 3.9 (s, 3 H), 2.58 (dt, *J^2^* = 16.8 Hz, *J^3^* = 5.7 Hz, 1 H), 2.48 (ddd, *J^2^* = 16.7 Hz, *J^3^* = 8.1 Hz, *J^3^* = 5.5 Hz, 1 H), 2.15 (s, 3 H), 2.09 – 2.04 (m, 2 H), 1.88 – 1.79 (m, 1 H), 1.78 – 1.69 (m, 1 H)

^13^C NMR (CDCl_3_, 100 MHz): δ= 170.75, 162.18, 153.16, 150.55, 139.17, 128.50, 128.44, 128.02, 124.10, 111.12, 71.20, 53.40, 29.17, 26.60, 21.53, 19.43

HRMS (ESI+): m/z

[M + H]+ calcd for C18H20NO3: 298.1443 ; found: 298.1417

**
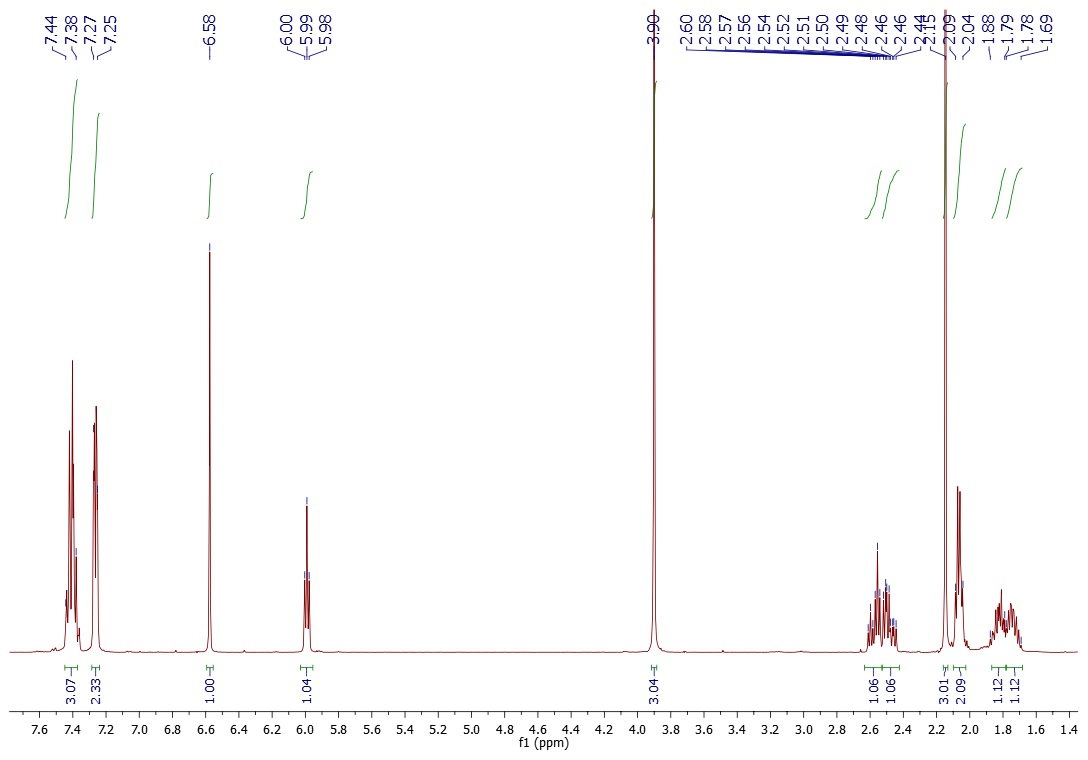
**

**Figure S11. ^1^H NMR of (2-methoxy-4-phenyl-5,6,7,8-tetrahydroquinolin-8-yl) acetate**

**
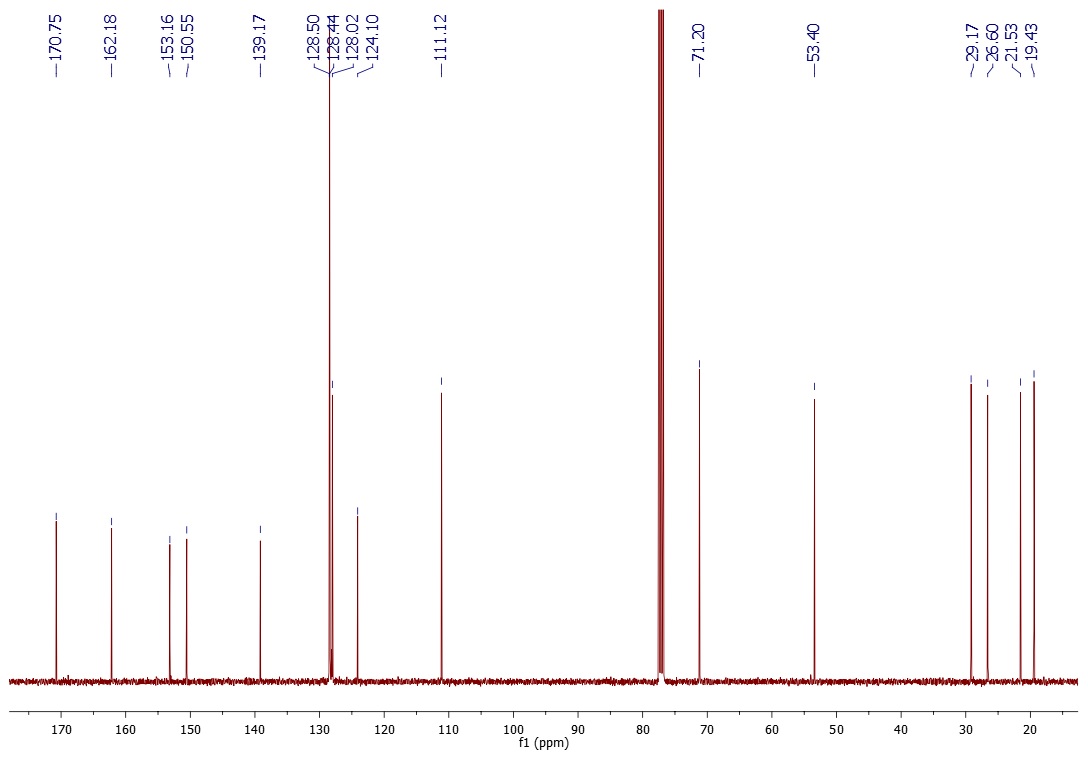
**

**Figure S12. ^13^C NMR of (2-methoxy-4-phenyl-5,6,7,8-tetrahydroquinolin-8-yl) acetate**

**7. 2-chloro-4-phenyl-5,6,7,8-tetrahydroquinolin-8-ol (16)**

A solution of KOH (0.19 g, 3.4 mmol) in 5 ml of methanol was placed in round-bottomed flask with stir bar and **14** (0.177 g, 0.59 mmol) was added**.** The mixture was left overnight in RT. After methanol evaporation the residue was diluted with ethyl acetate and washed twice with 2M HCl. The organic layer was dried with anhydrous MgSO_4_. Final product was purified by flash column chromatography (ethyl acetate : hexane 1:5). The title compound (0.134 g, 0.50 mmol, 85%) was obtained as white cristals.


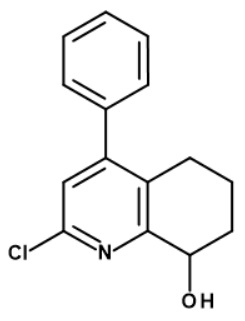
White cristals, yield: 85%, mp: 88 – 90^o^C

^1^H NMR (CDCl_3_, 400 MHz): δ= 7.47 – 7.42 (m, 3 H), 7.28 – 7.25 (m, 2 H), 7.12 (s, 1 H), 4.75 (t, *J* = 7.0 Hz, 1 H), 2.7 – 2.54 (m, 2 H), 2.29 – 2.22 (m, 1 H), 1.95 – 1.82 (m, 2 H), 1.76 – 1.65 (m, 1 H)

^13^C NMR (CDCl_3_, 100 MHz): δ= 159.14, 153.59, 148.32, 137.75, 128.88, 128.73, 128.69, 128.35, 123.73, 68.96, 30.28, 27.04, 19.43

HRMS (ESI+): m/z

[M + H]+ calcd for C15H15ClNO: 260.0842 ; found: 260.0878.

**
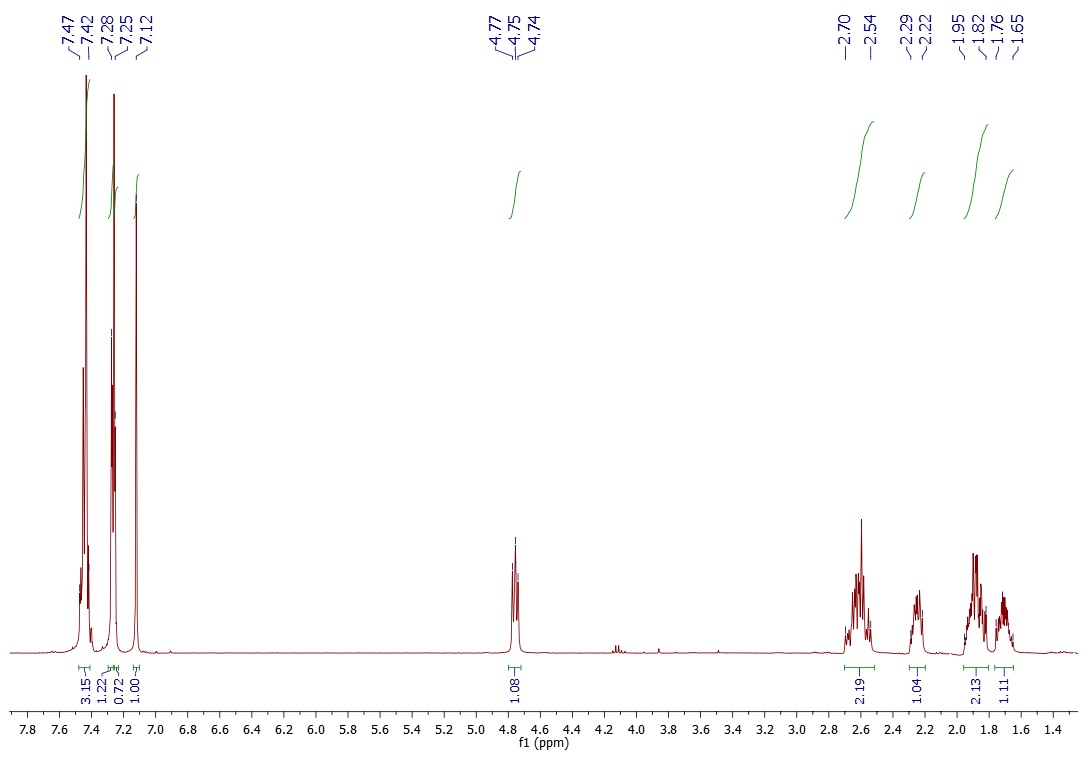
**

**Figure S13. 1H NMR of 2-chloro-4-phenyl-5,6,7,8-tetrahydroquinolin-8-ol**

**
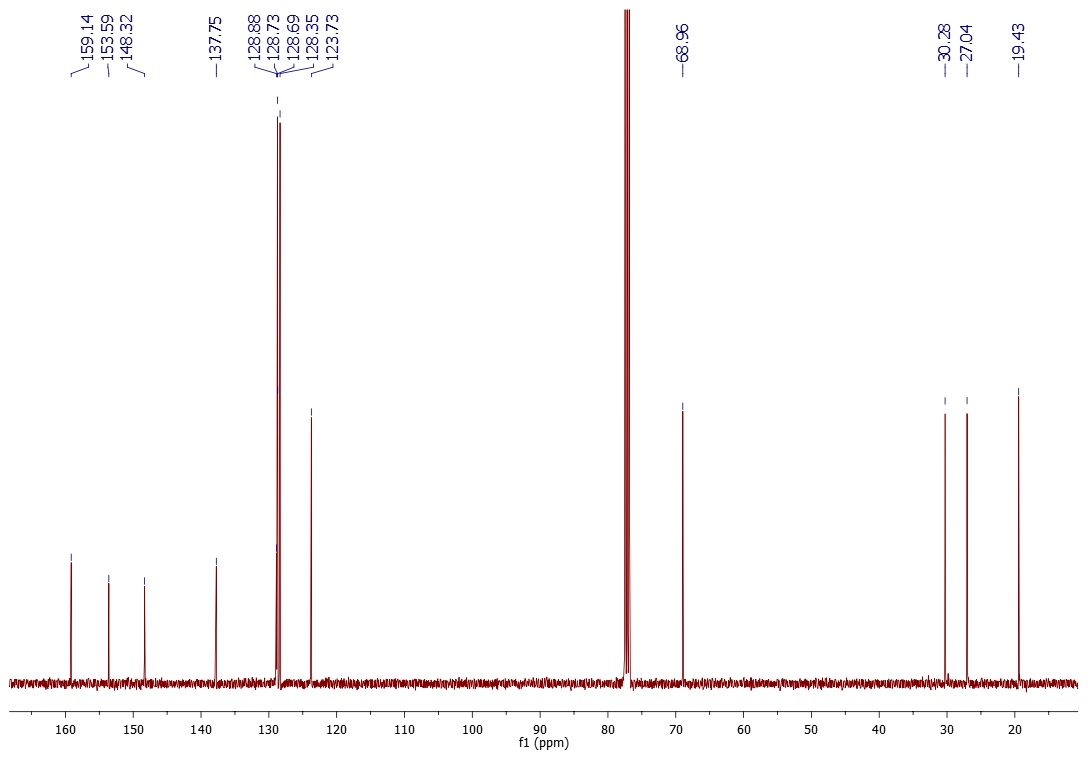
**

**Figure S14. ^13^C NMR of 2-chloro-4-phenyl-5,6,7,8-tetrahydroquinolin-8-ol**

**8. 2-methoxy-4-phenyl-5,6,7,8-tetrahydroquinolin-8-ol (17)**

Title compound was synthesised according to procedure of **16**. **17** (0.19, 0.74 mmol, 85%) was obtained as white cristals.

White cristals, yield: 85%, mp: 61 – 63^o^C


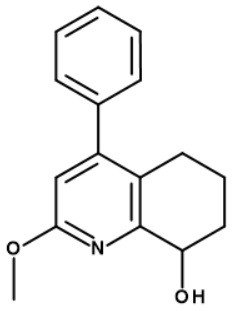
^1^H NMR (CDCl_3_, 400 MHz): δ= 7.44 – 7.39 (m, 3 H), 7.28 – 7.25 (m, 2 H), 6.56 (s, 1 H), 4.7 (dd, *J^3^* = 8.2 Hz, *J^3^* = 6.0 Hz, 1 H), 4.04 (s, 1 H), 3.97 (s, 3 H), 2.62 – 2.47 (m, 2 H), 2.31 – 2.25 (m, 1 H), 1.93 – 1.85 (m, 1 H), 1.83 – 1.63 (m, 2 H),

^13^C NMR (CDCl_3_, 100 MHz): δ= 159.19, 153.36, 148.48, 137.81, 128.70, 128.34, 128.22, 123.64, 110.16, 69.09, 54.02, 30.30, 27.01, 19.42

HRMS (ESI+): m/z

[M + H]+ calcd for C16H18NO2: 256.1338 ; found: 256.1369

**
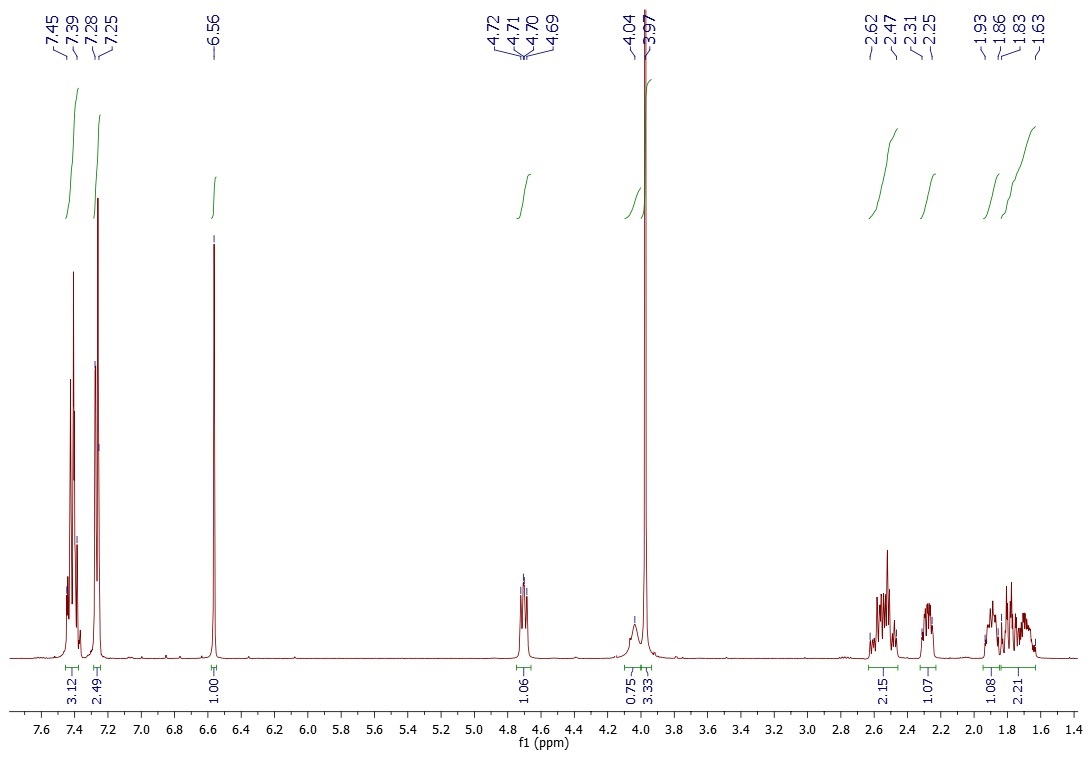
**

**Figure S15. ^1^H NMR of 2-methoxy-4-phenyl-5,6,7,8-tetrahydroquinolin-8-ol**

**
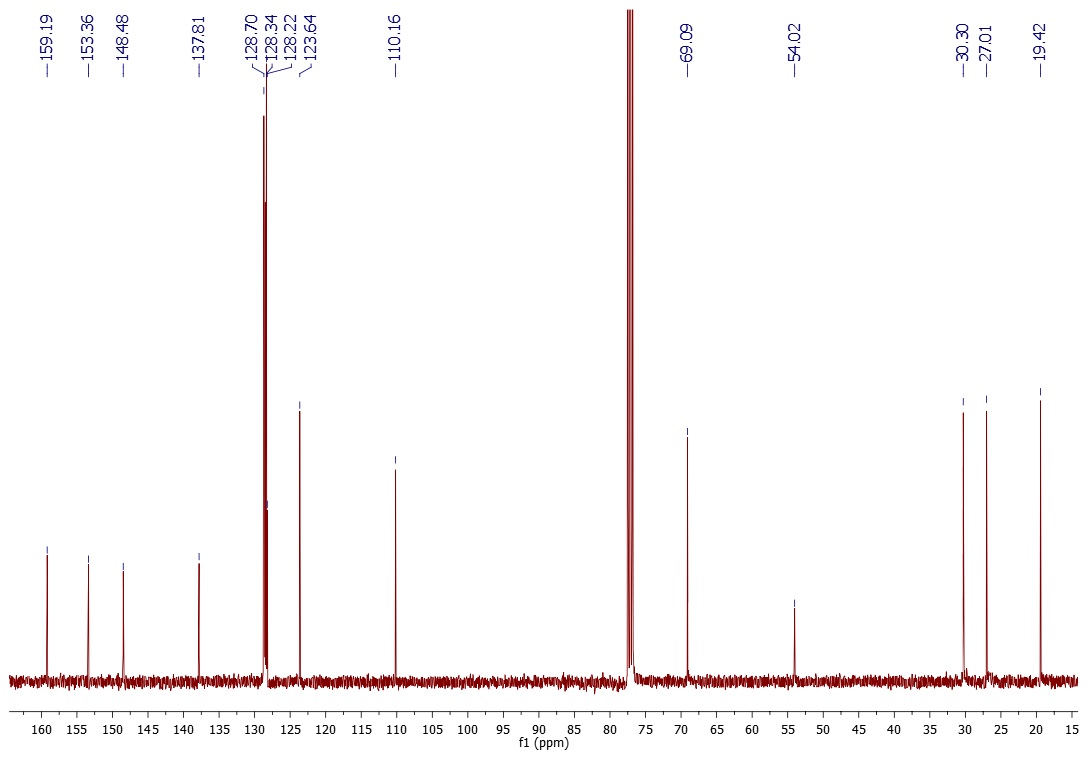
**

**Figure S16. ^13^C NMR of 2-methoxy-4-phenyl-5,6,7,8-tetrahydroquinolin-8-ol**

**General procedure for 18a-e**

A solution of coresponding isocyanate (0,3 mmol) in 3 ml of DCM was placed in round-bottomed flask with stir bar. Then NEt_3_ (0,32 mmol) was added and the mixture was left for 20 min. In the next step **16** (0.15 mmol) was added in one portion. The mixture was left overnight in RT. After solvent evaporation coresponding crude products were purified by flash column chromatography (ethyl acetate : hexane 1:6). Yields and characteristic of title compounds are collected in a table below.

**Table S1.**

| **Compound** | **Yield** | **Characteristic** |
| --- | --- | --- |
| 18a | 95 | White cristals |
| 18b | 88 | Bright yellow amorphous powder |
| 18c | 63 | White cristals |
| 18d | 62 | White amorphous powder |
| 18e | 65 | White amorphous powder |

**9. (2-chloro-4-phenyl-5,6,7,8-tetrahydroquinolin-8-yl) N-phenylcarbamate (18a)**


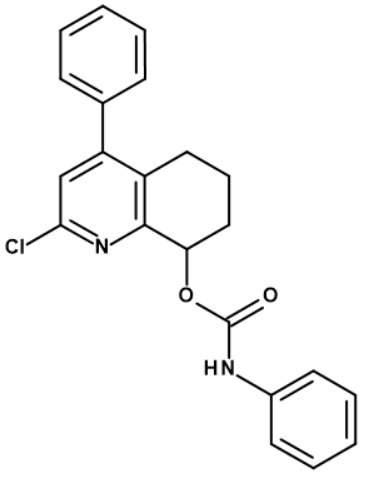


White cristals, yield: 95%, mp: 148 - 150^o^C

^1^H NMR (CDCl_3_, 400 MHz): δ= 7.49 – 7.4 (m, 5 H), 7.33 – 7.26 (m, 4 H), 7.17 (s, 1 H), 7.06 (t, *J* = 7.4 Hz, 1 H), 6.82 (s, 1 H), 5.91 (t, *J* = 4.6, 1 H), 2.68 (dt, *J^2^* = 17.4 Hz, *J^3^* = 5.1 Hz, 1 H), 2.57 (ddd, *J^2^* = 17.0 Hz, *J^3^* = 8.9 Hz, *J^3^* = 5.8 Hz, 1 H), 2.33 – 2.26 (m, 1 H), 2.15 – 2.07 (m, 1 H), 1.9 – 1.76 (m, 2 H)

^13^C NMR (CDCl_3_, 100 MHz): δ= 154.21, 153.52, 152.86, 149, 138.07, 137.62, 130.79, 129.17, 128.77, 128.74, 128.43, 124.59, 123.51, 118,84, 71.91, 29.06, 26.99, 18.53

HRMS (ESI+): m/z

[M + H]+ calcd for C22H20ClN2O2: 379.1213 ; found: 379.1258.

**
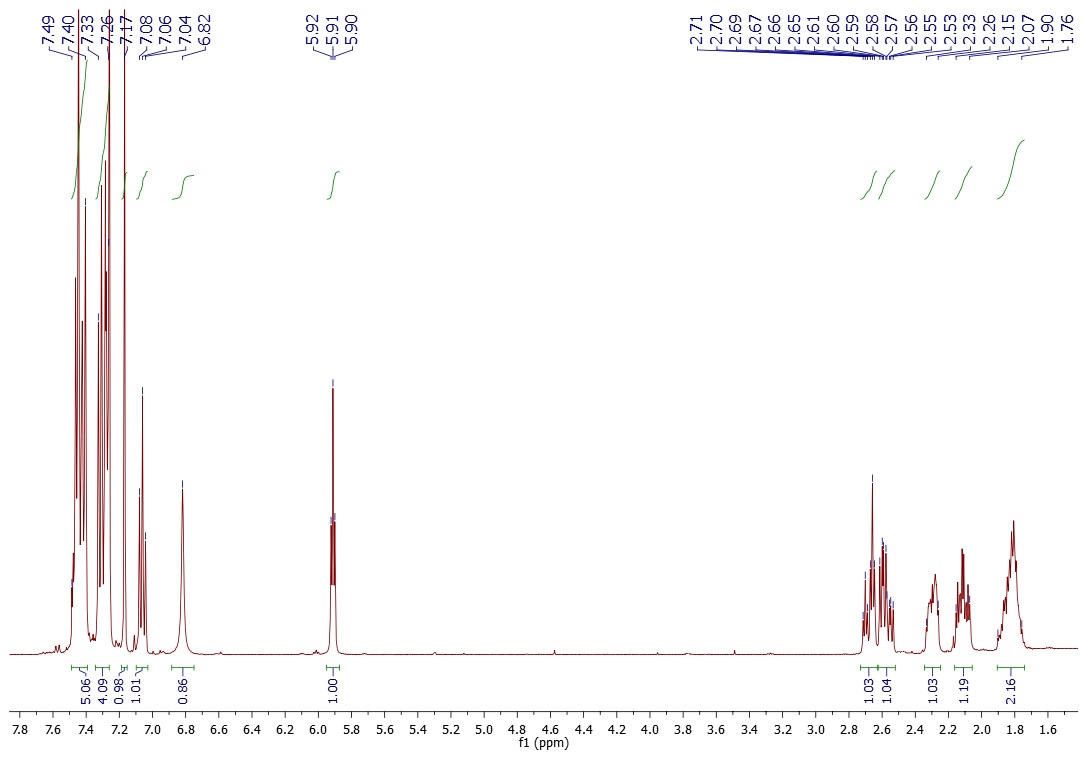
**

**Figure S17. ^1^H NMR of (2-chloro-4-phenyl-5,6,7,8-tetrahydroquinolin-8-yl) N-phenylcarbamate**

**
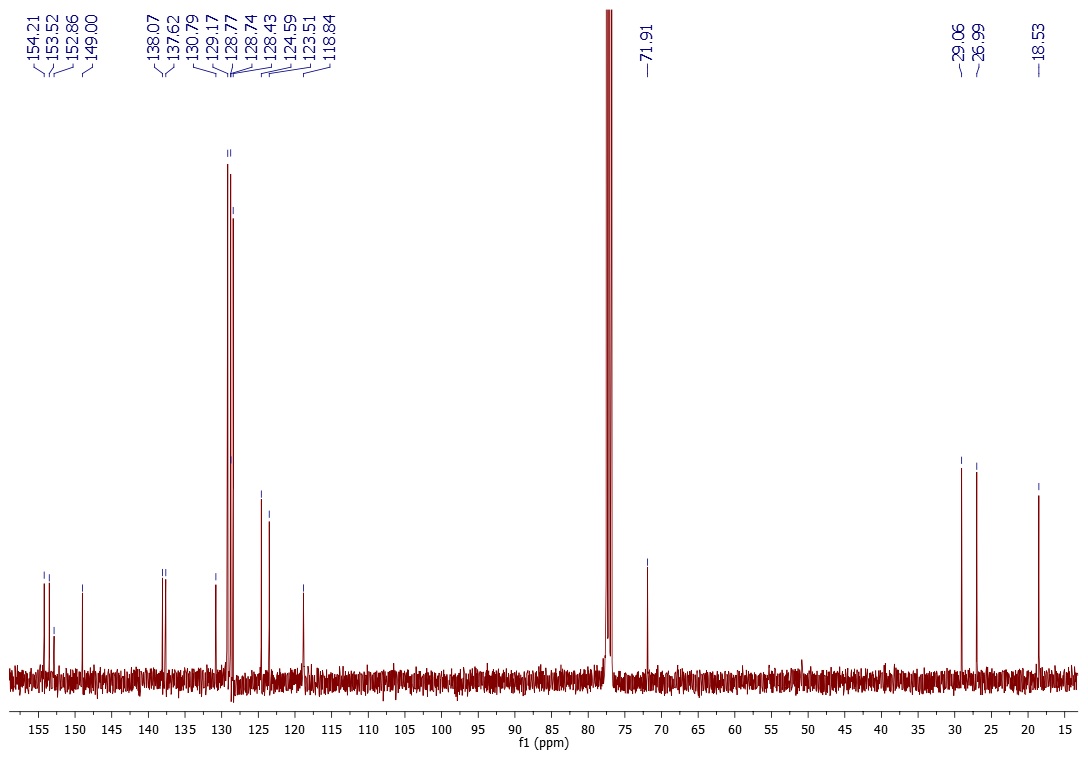
**

**Figure S18. ^13^C NMR of (2-chloro-4-phenyl-5,6,7,8-tetrahydroquinolin-8-yl) N-phenylcarbamate**

**10. (2-chloro-4-phenyl-5,6,7,8-tetrahydroquinolin-8-yl) N-(4-nitrophenyl)carbamate (18b)**


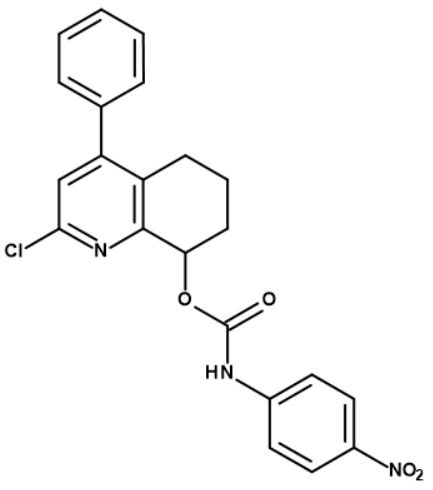


Bright yellow amorphous powder, yield: 88%

^1^H NMR (CDCl_3_, 400 MHz): δ= 8.21 – 8.17 (m, 2 H), 8.03 (s, 1 H), 7.6 – 7.56 (m, 2 H), 7.46 – 7.43 (m, 3 H), 7.25 – 7.23 (m, 2 H), 7.17 (s, 1 H), 5.91 (t, *J* = 4.6, 1 H), 2.70 – 2.54 (m, 2 H), 2.28 – 2.21 (m, 1 H), 2.16 – 2.08 (m, 1 H), 1.83 – 1.76 (m, 2 H)

^13^C NMR (CDCl_3_, 100 MHz): δ= 153.77, 153.73, 152.40, 148.99, 144.48, 143.04, 137.30, 130.92, 128.93, 128.85, 128.38, 125.33, 124.68, 117.89, 72.13, 29.09, 26.99, 18.49

HRMS (ESI+): m/z

[M + H]+ calcd for C22H19ClN3O4: 424.1064 ; found: 424.1096

**
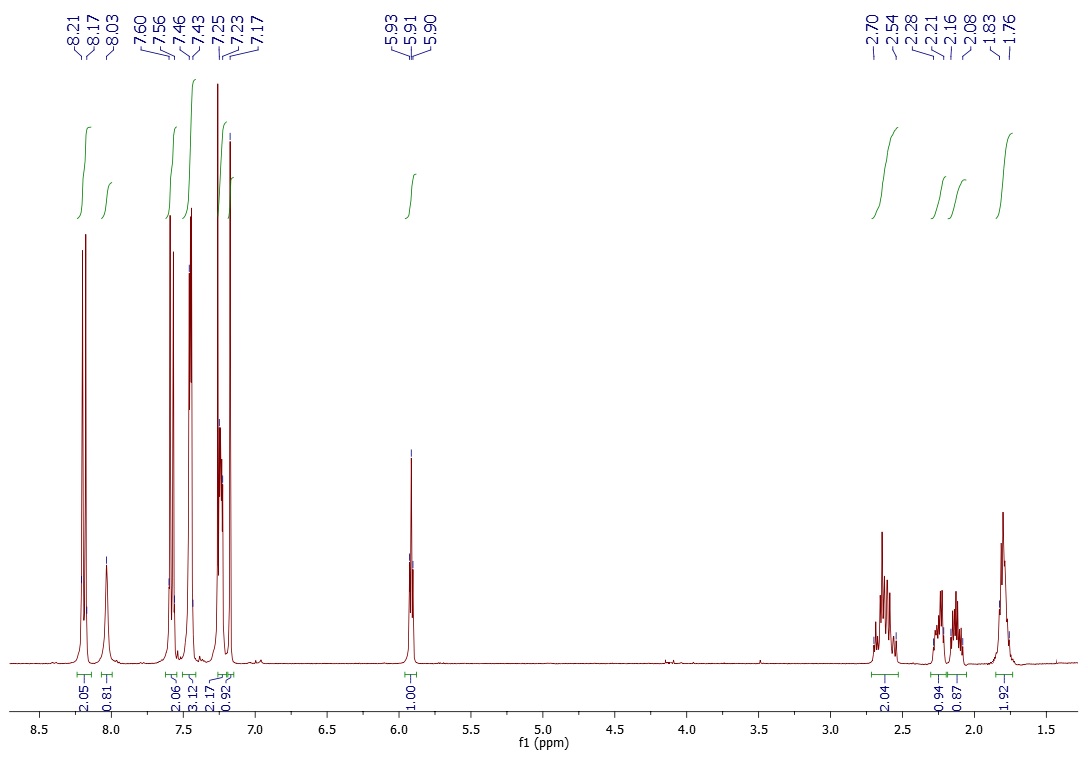
**

**Figure S19. ^1^H NMR of (2-chloro-4-phenyl-5,6,7,8-tetrahydroquinolin-8-yl) N-(4-nitrophenyl)carbamate**

**
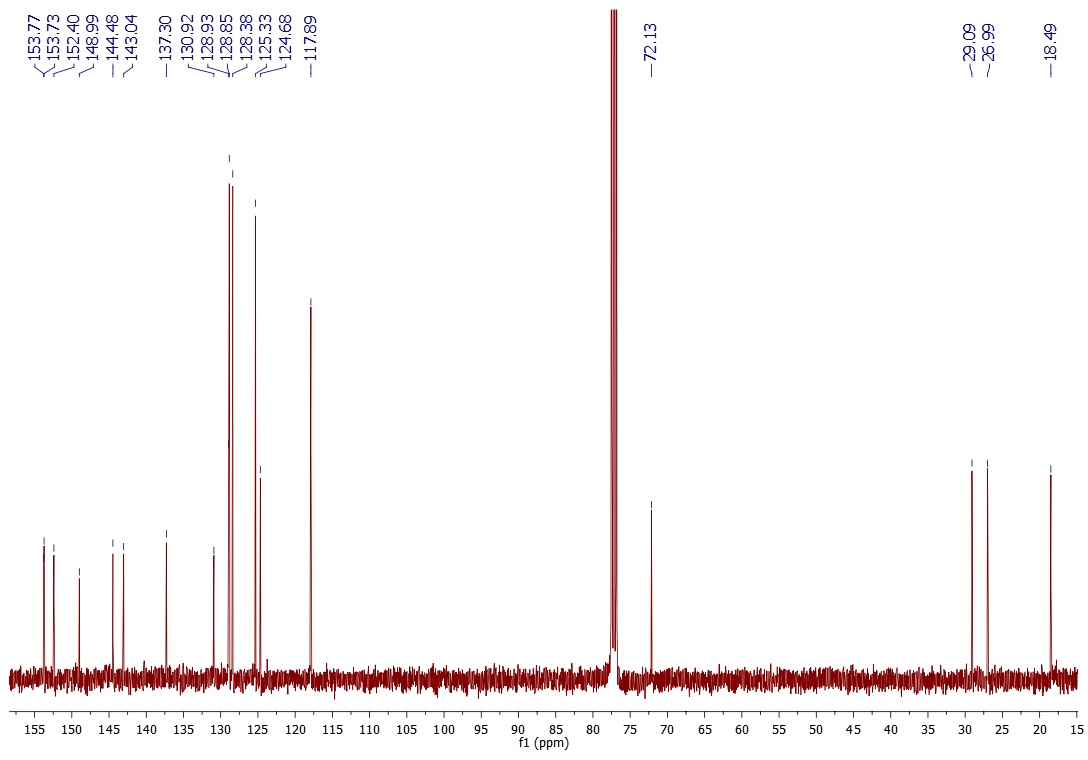
**

**Figure S20. ^13^C NMR of (2-chloro-4-phenyl-5,6,7,8-tetrahydroquinolin-8-yl) N-(4-nitrophenyl)carbamate**

**11. (2-chloro-4-phenyl-5,6,7,8-tetrahydroquinolin-8-yl) N-(4-fluorophenyl)carbamate (18c)**


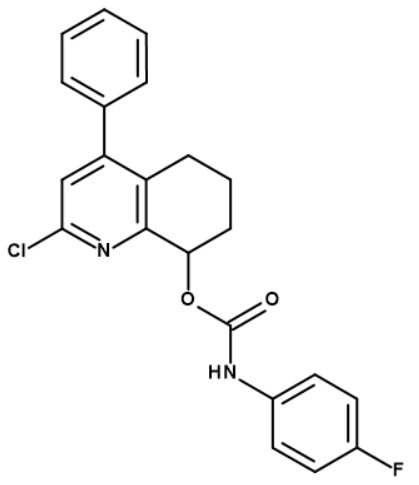


White cristals, yield: 63%, mp: 113 – 115^o^C

^1^H NMR (CDCl_3_, 400 MHz): δ= 7.48 – 7.43 (m, 3 H), 7.39 – 7.36 (m, 2 H), 7.27 – 7.25 (m, 2 H), 7.16 (s, 1 H), 7.02 – 6.97 (m, 3 H), 5.89 (t, *J* = 4.7, 1 H), 2.67 (dt, *J^2^* = 17.4 Hz, *J^3^* = 5.2 Hz, 1 H), 2.57 (ddd, *J^2^* = 17.0 Hz, *J^3^* = 8.4 Hz, *J^3^* = 6.2 Hz, 1 H), 2.30 – 2.23 (m, 1 H), 2.15 – 2.06 (m, 1 H), 1.84 – 1.75 (m, 2 H)

^13^C NMR (CDCl_3_, 100 MHz): δ= 159.07 (d, *J^1^* = 240,5 Hz), 154.20, 153.48, 153.10, 149, 137.57, 134.14 (d, *J^4^* = 2.6 Hz), 130.74, 128.77, 128.75, 128.41, 124.53, 120.59 (d, *J^3^* = 4.5 Hz), 115.74 (d, *J^2^* = 22.4 Hz), 71.91, 29.09, 26.99, 18.53

HRMS (ESI+): m/z

[M + H]+ calcd for C22H19ClFN2O2: 397.1119 ; found: 397.1149

**
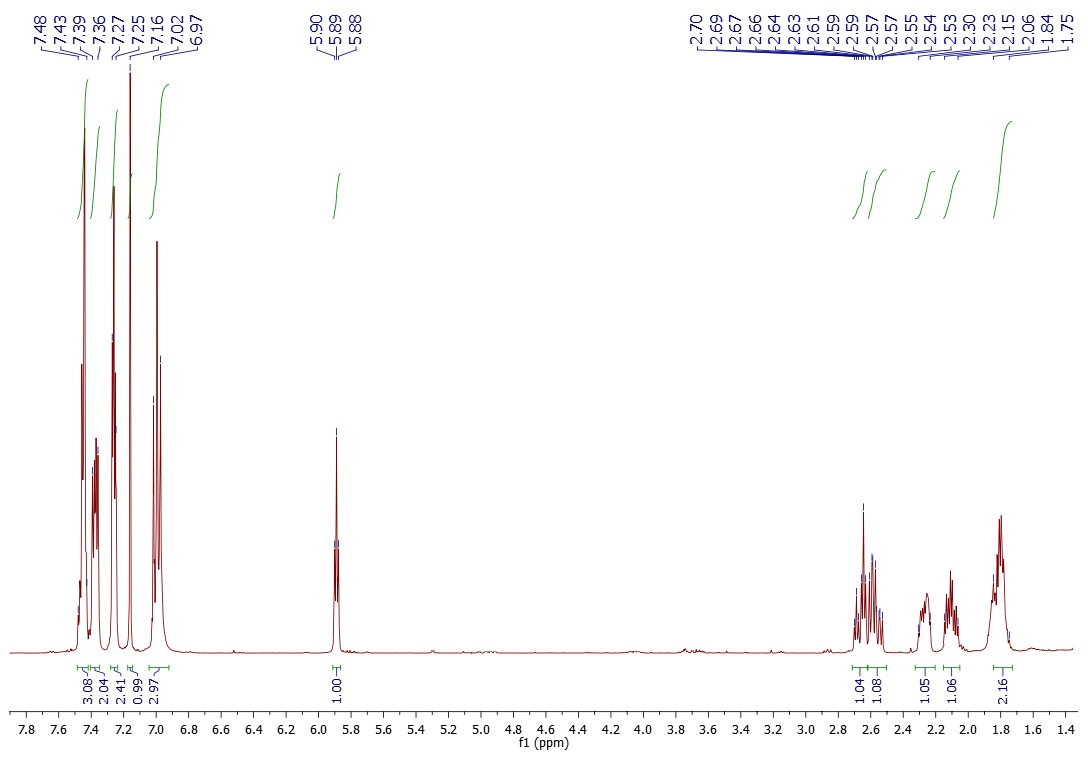
Figure S21. ^1^H NMR of (2-chloro-4-phenyl-5,6,7,8-tetrahydroquinolin-8-yl) N-(4-fluorophenyl)carbamate**

**
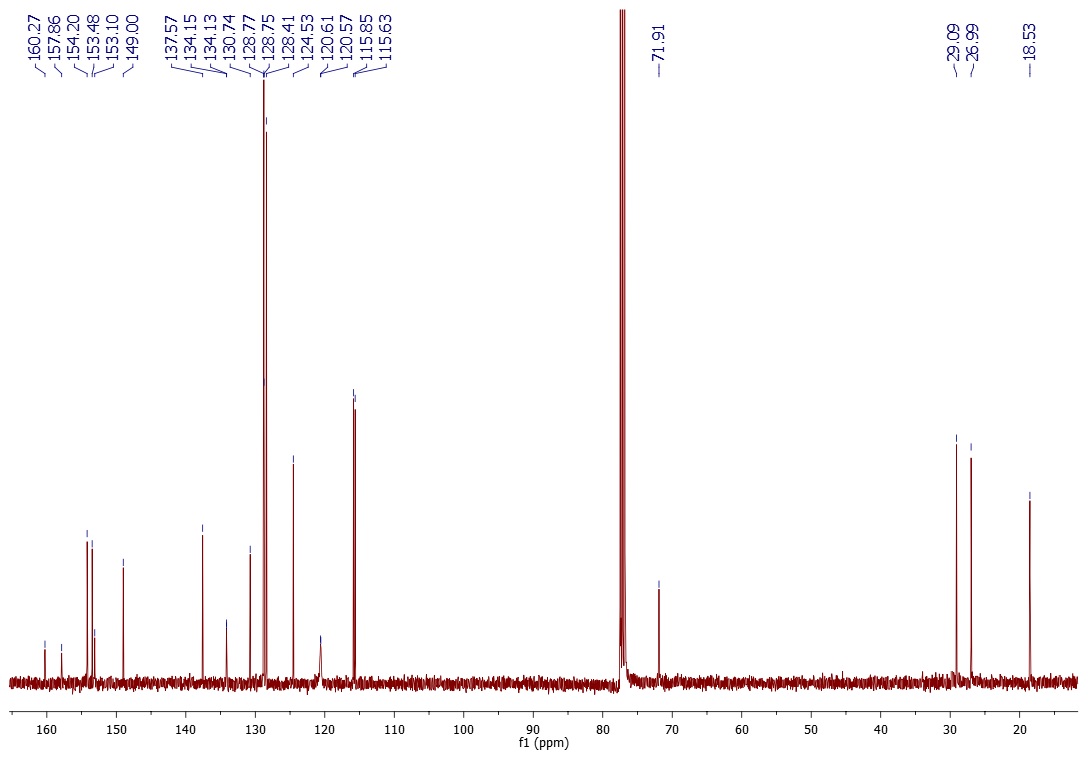
**

**Figure S22. ^13^C NMR of (2-chloro-4-phenyl-5,6,7,8-tetrahydroquinolin-8-yl) N-(4-fluorophenyl)carbamate**

**12. (2-chloro-4-phenyl-5,6,7,8-tetrahydroquinolin-8-yl) N-(3-fluorophenyl)carbamate (18d)**


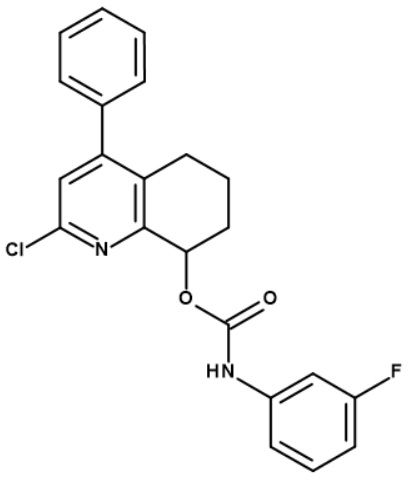
White amorphous powder, yield: 62%

^1^H NMR (CDCl_3_, 400 MHz): δ= 7.48 – 7.43 (m, 3 H), 7.35 (dt, *J* = 11.1 Hz, *J* = 2.0 Hz, 1 H), 7.27 – 7.23 (m, 3 H), 7.22 – 7.2 (m, 1 H), 7.16 (s, 1 H), 7.06 (dd, *J_1_* = 8.1 Hz, *J_2_*= 1.3 Hz, 1 H), 6.74 (tdd, *J* = 8.3 Hz, *J* = 2.3 Hz, *J* = 0.6 Hz, 1 H), 5.98 (t, *J* = 4.6 Hz, 1 H), 2.66 (dt, *J^2^* = 17.4 Hz, *J^3^* = 5.1 Hz, 1 H), 2.61 – 2.53 (m, 1 H), 2.30 – 2.23 (m, 1 H), 2.15 – 2.06 (m, 1 H), 1.84 – 1.77 (m, 2 H)

^13^C NMR (CDCl_3_, 100 MHz): δ= 163.31 (d, *J^1^* = 22.7 Hz), 154.07, 153.50, 152.67, 149, 139.85 (d, *J^3^* = 11.0 Hz), 137.53, 130.76, 130.16 (d, *J^3^* = 9.4 Hz), 128.77, 128.40, 124.55, 114.03 (d, *J^4^* = 2.7 Hz), 110.04 (d, *J^2^* = 21.3 Hz), 106.16 (d, *J^2^* = 26.7 Hz), 71.93, 29.06, 26.98, 18.50

HRMS (ESI+): m/z

[M + H]+ calcd for C22H19ClFN2O2: 397.1119 ; found: 397.1148

**
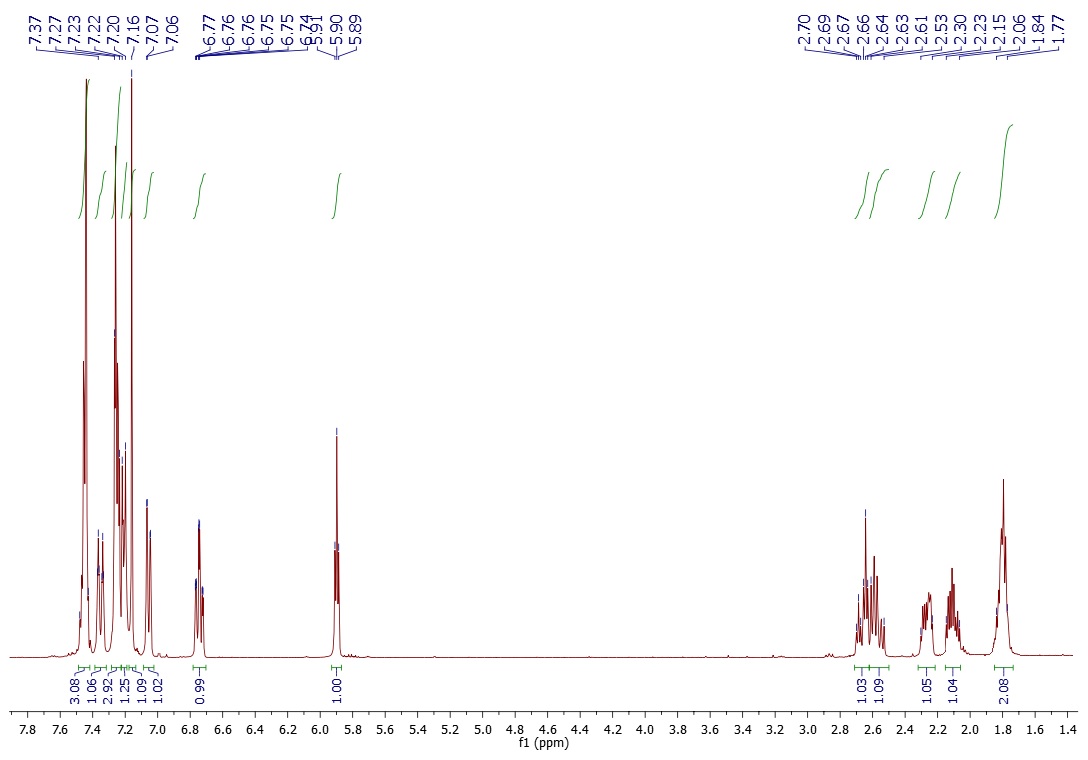
**

**Figure S23. ^1^H NMR of (2-chloro-4-phenyl-5,6,7,8-tetrahydroquinolin-8-yl) N-(3-fluorophenyl)carbamate**

**
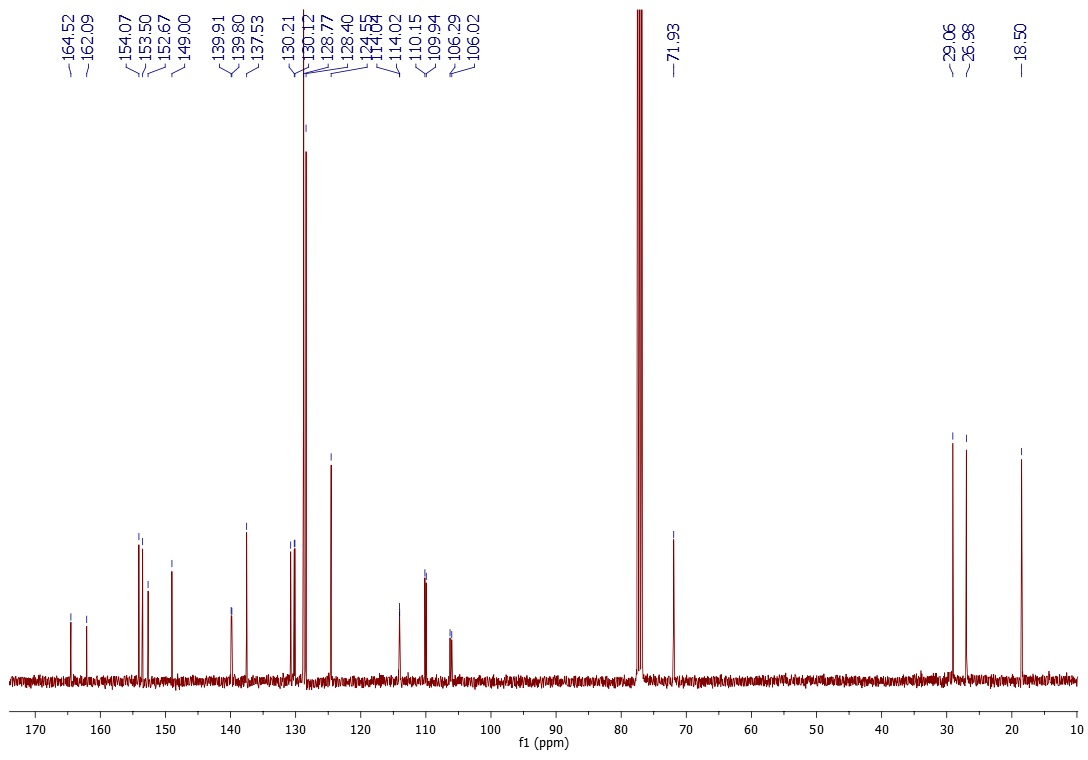
**

**Figure S24. ^13^C NMR of (2-chloro-4-phenyl-5,6,7,8-tetrahydroquinolin-8-yl) N-(3-fluorophenyl)carbamate**

**13. (2-chloro-4-phenyl-5,6,7,8-tetrahydroquinolin-8-yl) N-(4-chlorophenyl)carbamate (18e)**


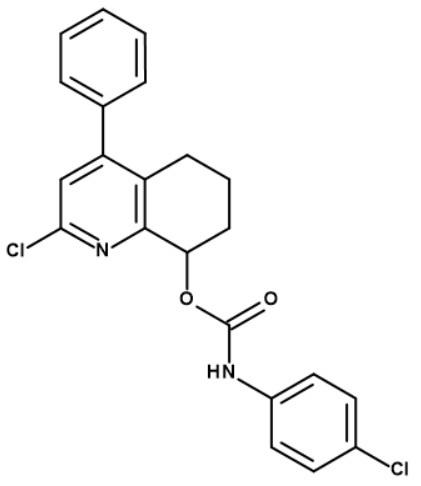
White amorphous powder, yield: 65%

^1^H NMR (CDCl_3_, 400 MHz): δ= 7.46 – 7.43 (m, 3 H), 7.38 – 7.36 (m, 2 H), 7.28 – 7.23 (m, 5H) 7.15 (s, 1 H), 5.88 (t, *J* = 4.6, 1 H), 2.65 (dt, *J^2^* = 17.4 Hz, *J^3^* = 5.2 Hz, 1 H), 2.60 – 2.52 (m, 1 H), 2.29 – 2.22 (m, 1 H), 2.14 – 2.04 (m, 1 H), 1.82 – 1.76 (m, 2 H)

^13^C NMR (CDCl_3_, 100 MHz): δ= 154.13, 153.51, 152.85, 148.96, 137.48, 136.88, 130.75, 129.07, 128.77, 128.39, 128.30, 124.51, 120.03, 71.79, 29.09, 26.99, 18.49

HRMS (ESI+): m/z

[M + H]+ calcd for C22H19Cl2N2O2: 413.0824 ; found: 413.0869

**
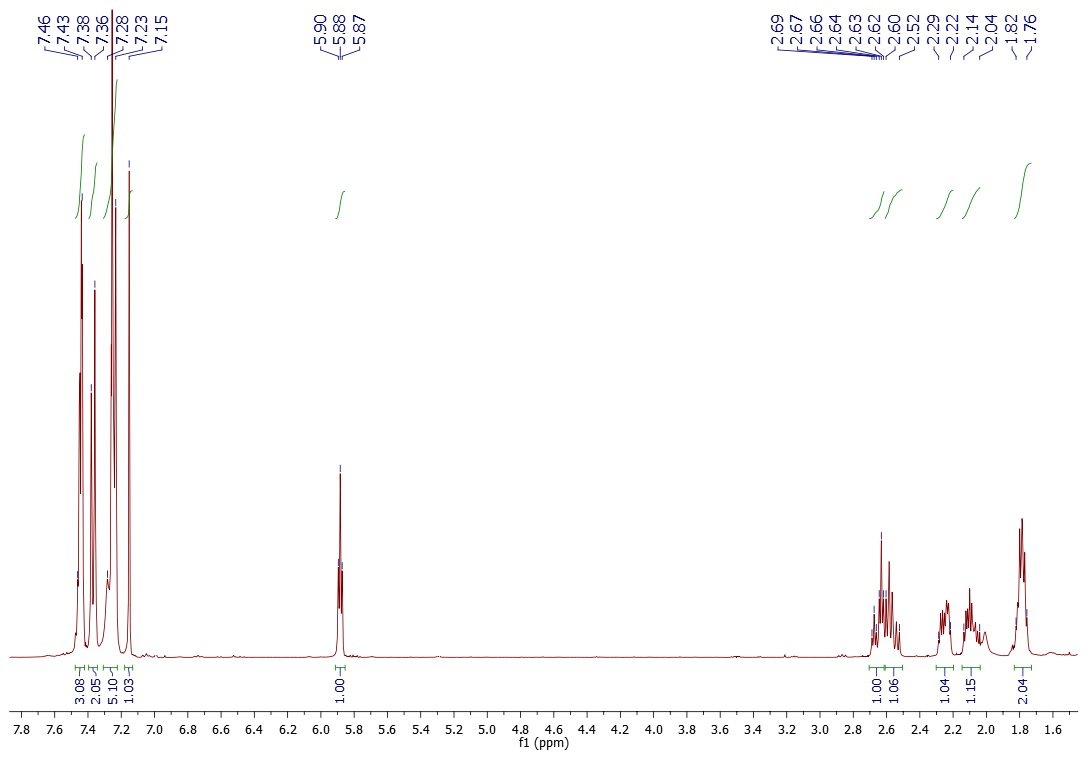
**

**Figure S25. ^1^H NMR of (2-chloro-4-phenyl-5,6,7,8-tetrahydroquinolin-8-yl) N-(4-chlorophenyl)carbamate**

**
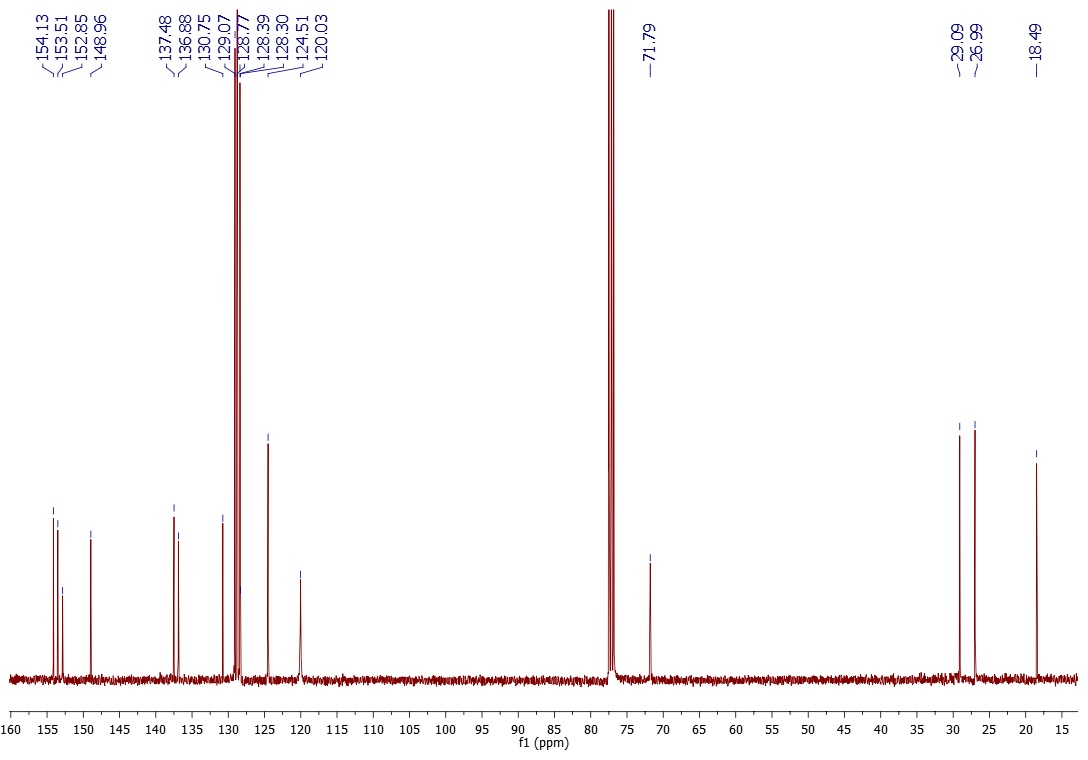
**

**Figure S26. ^13^C NMR of (2-chloro-4-phenyl-5,6,7,8-tetrahydroquinolin-8-yl) N-(4-chlorophenyl)carbamate**

**General procedure for 19a-e and 20a-e**

Title compounds were synthesised according to procedure of **18a-e**.

After solvent evaporation coresponding crude products were purified twice by flash column chromatography (DCM 100% and ethyl acetate : hexane 1:5). Yields and characteristic of title compounds are collected in a table below.

**Table S2.**

| **Compound** | **Yield** | **Characteristic** |
| --- | --- | --- |
| 19a | 14% | Colorless oil |
| 19b | 22% | Bright yellow cristals |
| 19c | 24% | White cristals |
| 19d | 19% | Colorless oil |
| 19e | 21% | White cristals |
| 20a | 80% | White cristals |
| 20b | 70% | Bright yellow cristals |
| 20c | 64% | White amorphous powder |
| 20d | 63% | White amorphous powder |
| 20e | 69% | White amorphous powder |

**14. (2-methoxy-4-phenyl-5,6,7,8-tetrahydroquinolin-8-yl) N-phenylcarbamate (19a)**


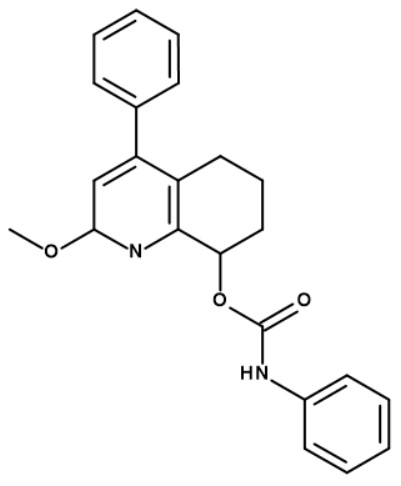


Colorless oil, yield: 14%

^1^H NMR (CDCl_3_, 400 MHz): δ= 7.45 – 7.40 (m, 5 H), 7.33 – 7.27 (m, 4 H), 7.06 (t, *J* = 7.4, 1 H), 6.88 (s, 1 H), 6.61 (s, 1 H), 5.96 (t, *J* = 5.3, 1 H), 3.90 (s, 3 H), 2.59 (dt, *J^2^* = 16.9 Hz, *J^3^* = 5.7 Hz, 1 H), 2.51 (ddd, *J^2^* = 16.5 Hz, *J^3^* = 8.0 Hz, *J^3^* = 5.6 Hz, 1 H), 2.22 – 2.11 (m, 2 H), 1.91 – 1.74 (m, 2 H)

^13^C NMR (CDCl_3_, 100 MHz): δ= 162.10, 153.93, 153.43, 150.43, 138.93, 138.31, 129.19, 128.54, 128.49, 128.25, 124.33, 123.40, 118.68, 111.13, 71.93, 53.93, 29.24, 26.59, 19.37

HRMS (ESI+): m/z

[M + H]+ calcd for C23H23N2O3: 375.1709 ; found: 375.1711

**
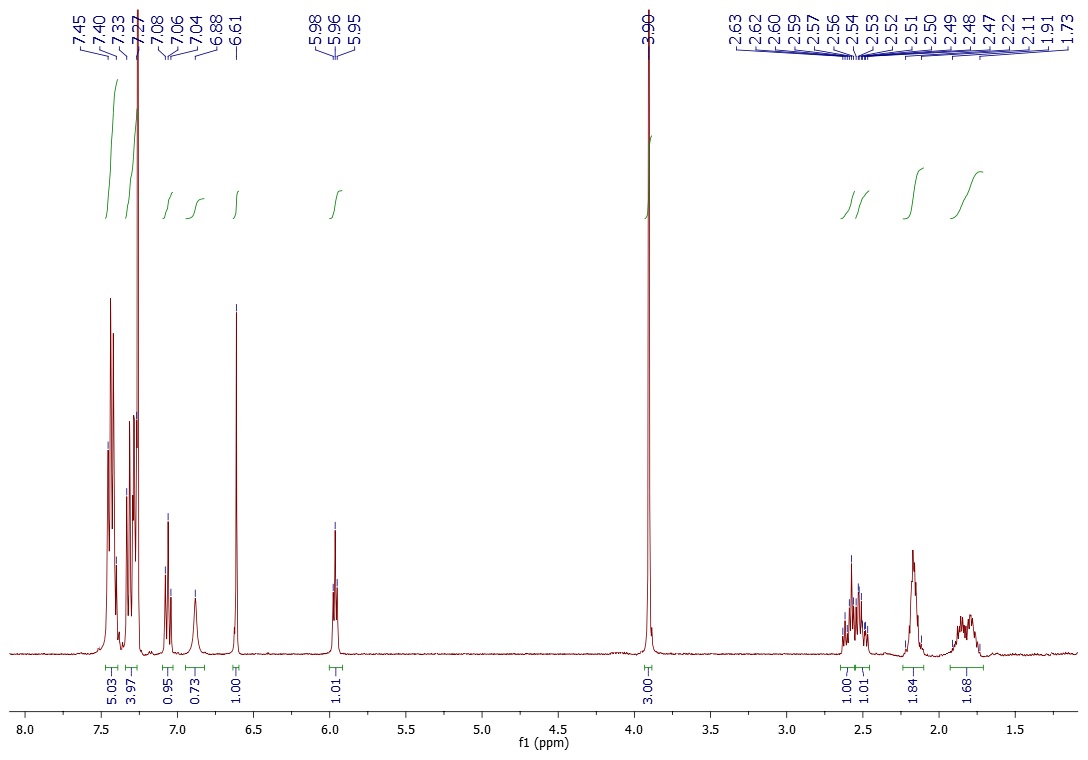
**

**Figure S27. ^1^H NMR of (2-methoxy-4-phenyl-5,6,7,8-tetrahydroquinolin-8-yl) N-phenylcarbamate**

**
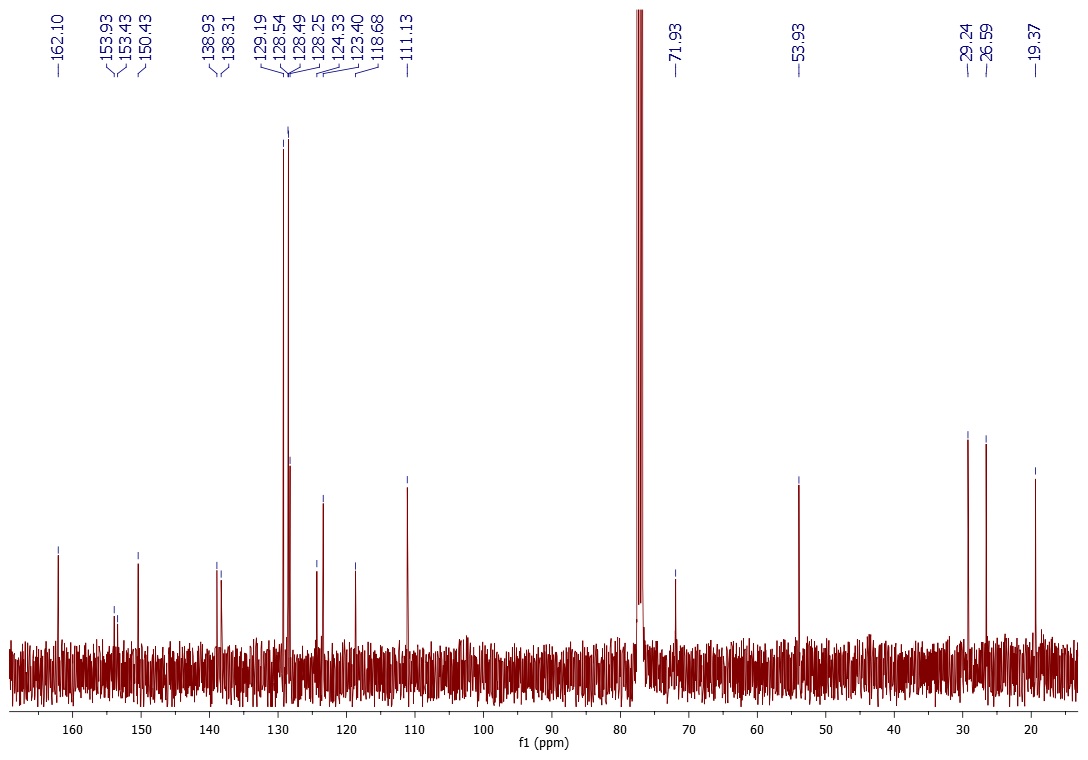
**

**Figure S28. ^13^C NMR of (2-methoxy-4-phenyl-5,6,7,8-tetrahydroquinolin-8-yl) N-phenylcarbamate**

**15. (2-methoxy-4-phenyl-5,6,7,8-tetrahydroquinolin-8-yl) N-(4-nitrophenyl)carbamate (19b)**


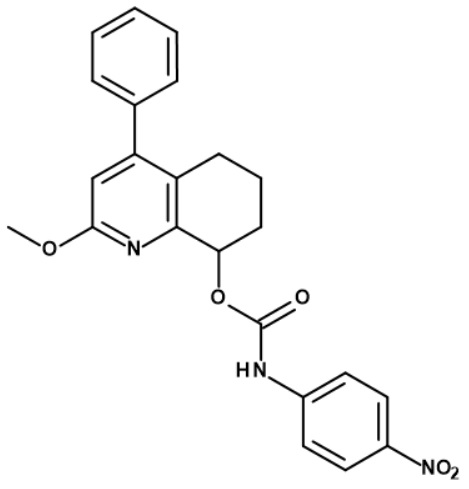


Bright yellow cristals, yield: 22%, mp: 183 – 185^o^C

^1^H NMR (CDCl_3_, 400 MHz): δ= 8.21 (d, *J* = 9.1 Hz, 2 H), 7.62 (d, *J* = 9.1 Hz, 2 H), 7.46 – 7.41 (m, 3 H), 7.36 (s, 1H), 7.27 – 7.25 (m, 2 H), 6.63 (s, 1 H), 5.98 (t, *J* = 5.2, 1 H), 3.88 (s, 3 H), 2.63 – 2.48 (m, 2 H), 2.22 – 2.18 (m, 2 H), 1.88 – 1.76 (m, 2 H)

^13^C NMR (CDCl_3_, 100 MHz): δ= 162.19, 154.13, 152.77, 149.87, 149.83, 144.38, 143.08, 138.71, 128.60, 128.45, 128.38, 125.40, 117.83, 111.22, 72.73, 53.94, 29.15, 26.53, 19.32

HRMS (ESI+): m/z

[M + H]+ calcd for C23H22N3O5: 420.1559 ; found: 420.1556

**
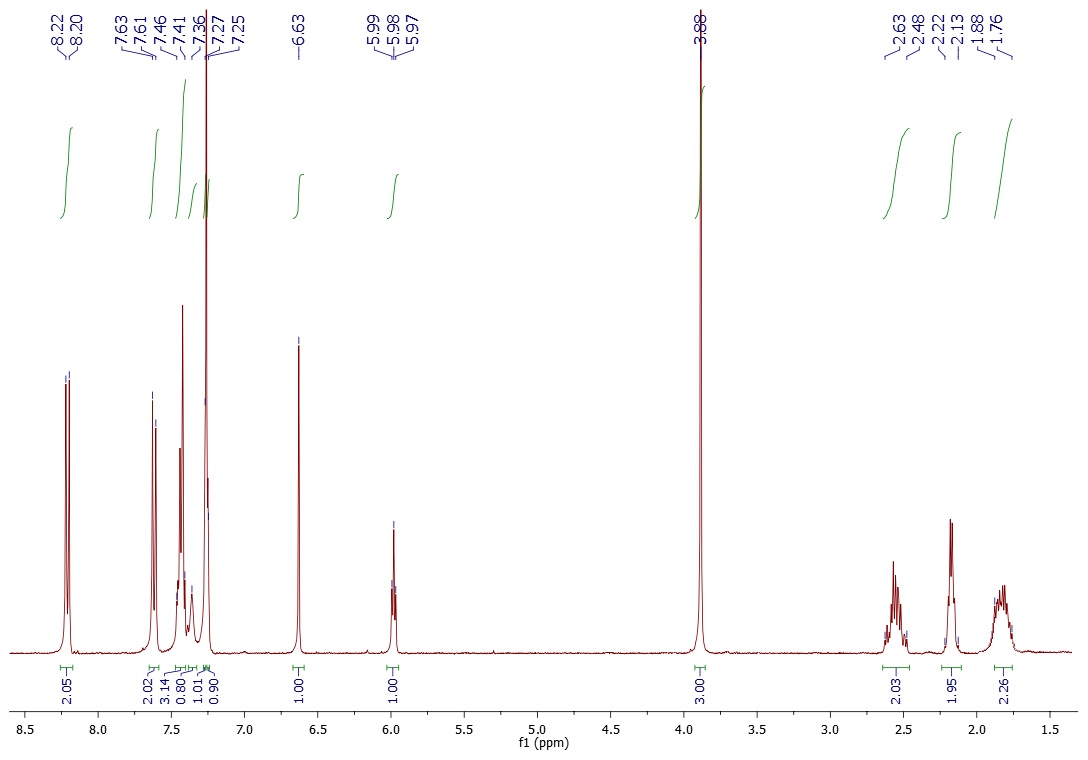
**

**Figure S29. ^1^H NMR of (2-methoxy-4-phenyl-5,6,7,8-tetrahydroquinolin-8-yl) N-(4-nitrophenyl)carbamate**

**
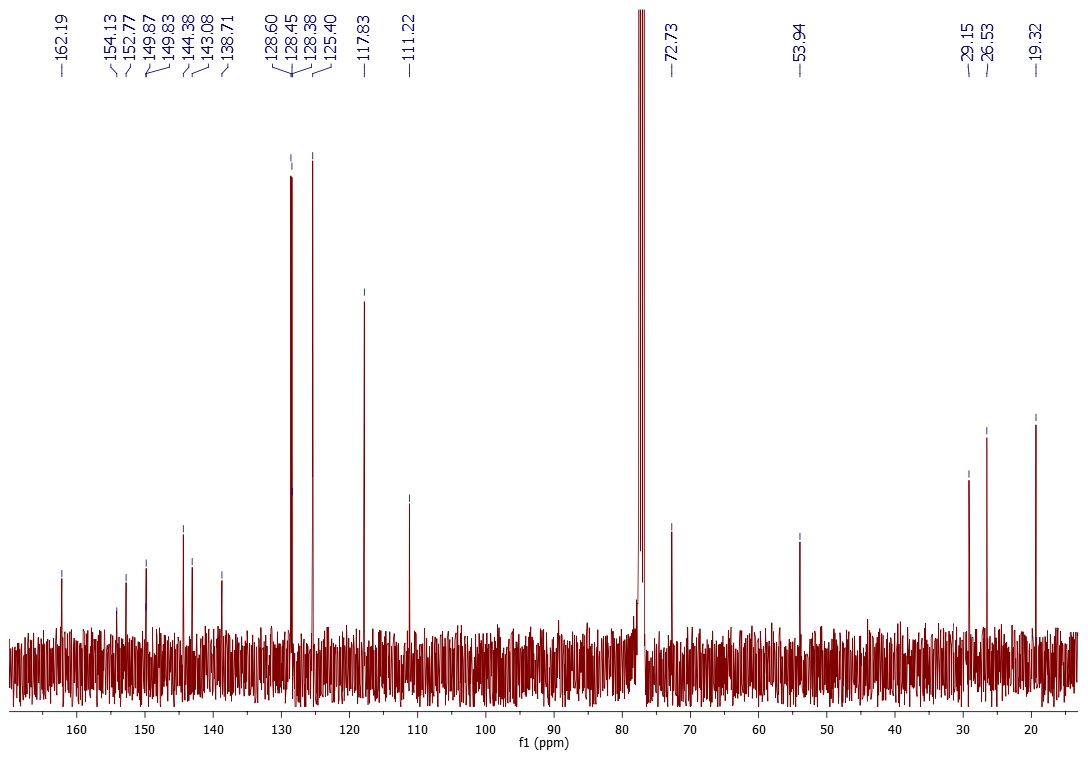
**

**Figure S30. ^13^C NMR of (2-methoxy-4-phenyl-5,6,7,8-tetrahydroquinolin-8-yl) N-(4-nitrophenyl)carbamate**

**16. (2-methoxy-4-phenyl-5,6,7,8-tetrahydroquinolin-8-yl) N-(4-fluorophenyl)carbamate (19c)**


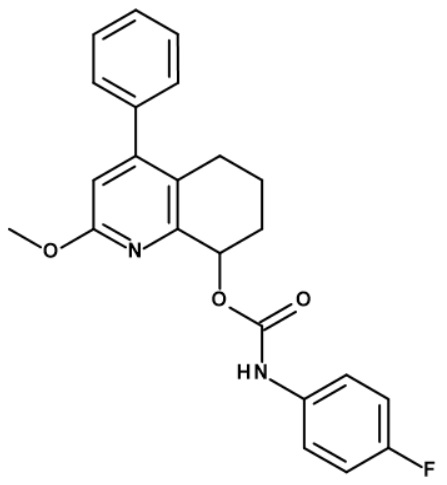


White cristals, yield: 24%, mp: 125 – 127^o^C

^1^H NMR (CDCl_3_, 400 MHz): δ= 7.45 – 7.38 (m, 5 H), 7.29 – 7.26 (m, 2 H), (m, 3 H), 7.05 – 6.98 (m, 2 H), 6.79 (s, 1 H), 6.60 (s, 1 H), 5.95 (t, *J* = 5.2, 1 H), 3.87 (s, 3 H), 2.59 (dt, *J^2^* = 16.9 Hz, *J^3^* = 5.7 Hz, 1 H), 2.50 (ddd, *J^2^* = 16.6 Hz, *J^3^* = 8.0 Hz, *J^3^* = 5.6 Hz, 1 H), 2.21 – 2.11 (m, 2 H), 1.90 – 1.75 (m, 2 H)

^13^C NMR (CDCl_3_, 100 MHz): δ= 162.22, 159.02 (d, *J^1^* = 240,6 Hz), 153.64, 153.53, 150.51, 139.03, 134.31 (d, *J^4^* = 2.6 Hz), 128.51, 128.15, 124.18, 120.38 (d, *J^3^* = 4.3 Hz), 115.80 (d, *J^2^* = 22.4 Hz), 111.20, 72.19, 53.65, 29.85, 27.06, 19.39

HRMS (ESI+): m/z

[M + H]+ calcd for C23H22FN2O3: 393.1614 ; found: 393.1641

**
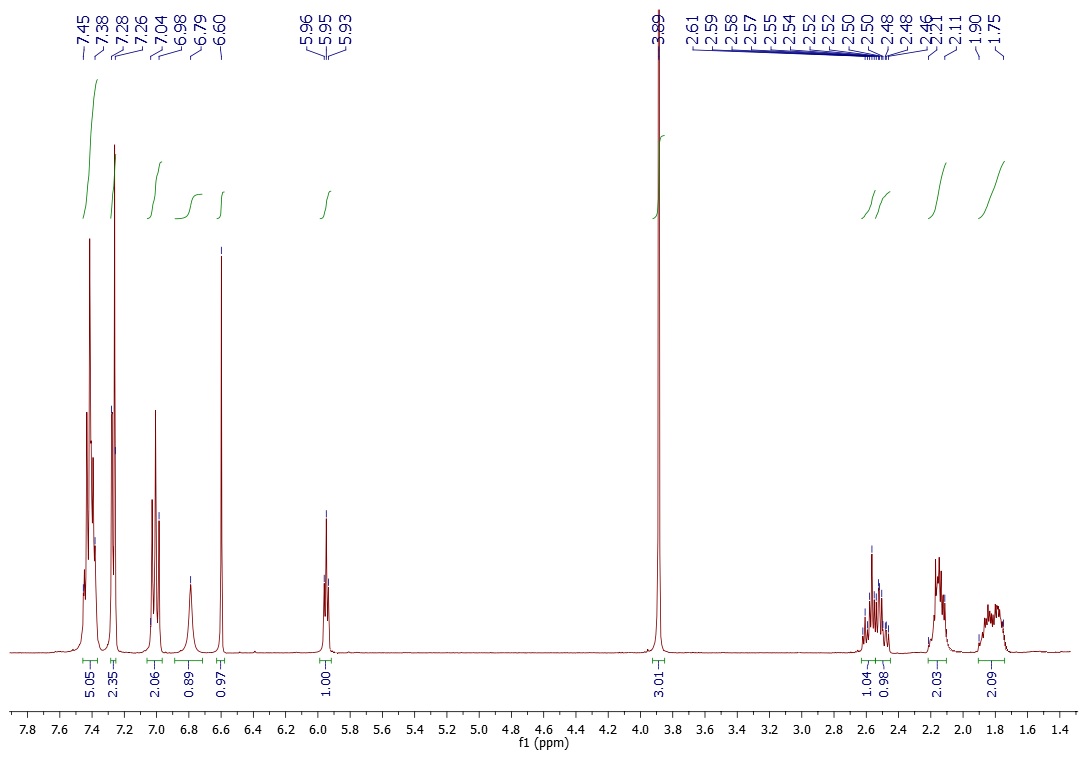
**

**Figure S31. ^1^H NMR of (2-methoxy-4-phenyl-5,6,7,8-tetrahydroquinolin-8-yl) N-(4-fluorophenyl)carbamate**

**
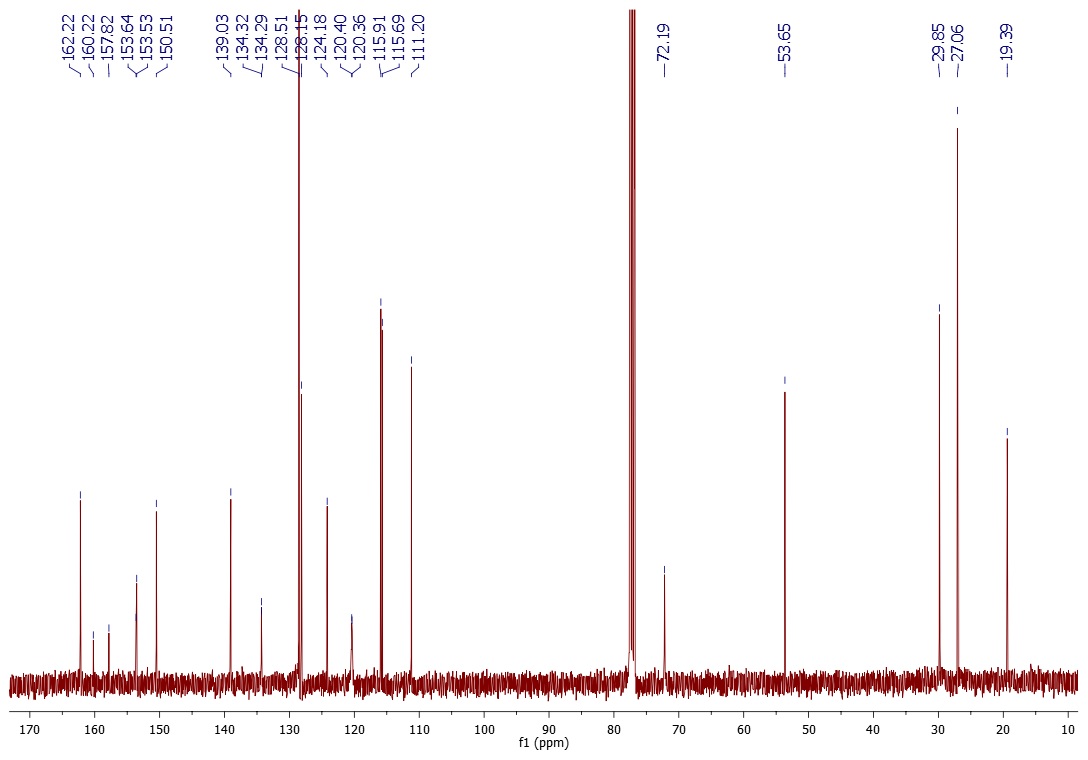
**

**Figure S32. ^13^C NMR of (2-methoxy-4-phenyl-5,6,7,8-tetrahydroquinolin-8-yl) N-(4-fluorophenyl)carbamate**

**17. (2-methoxy-4-phenyl-5,6,7,8-tetrahydroquinolin-8-yl) N-(3-fluorophenyl)carbamate (19d)**


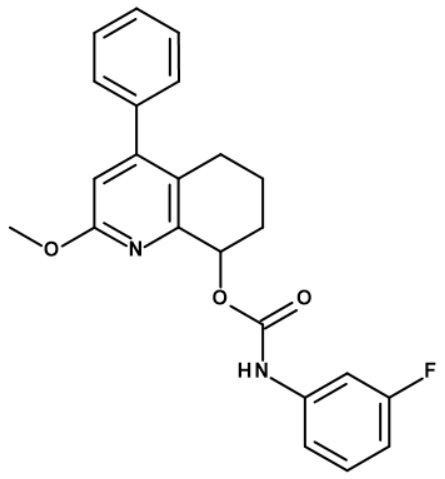


Colorless oil, yield: 19%

^1^H NMR (CDCl_3_, 400 MHz): δ= 7.46 – 7.38 (m, 4 H), 7.28 – 7.21 (m, 3H), 7.06 (dd, *J^3^* = 8.0 Hz, *J^5^* = 1.1 Hz, 1 H), 6.89 (s, 1 H), 6.75 (td, *J^3^* = 8.2 Hz, *J^3^* = 2.2 Hz, 1 H), 6.60 (s, 1 H), 5.96 (t, *J* = 5.2 Hz, 1 H), 3.88 (s, 3 H), 2.59 (dt, *J^2^* = 17.0 Hz, *J^3^* = 5.7 Hz, 1 H), 2.50 (ddd, *J^2^* = 16.5 Hz, *J^3^* = 8.0 Hz, *J^3^* = 5.7 Hz, 1 H), 2.18 – 2.09 (m, 2 H), 1.90 – 1.73 (m, 2 H)

^13^C NMR (CDCl_3_, 100 MHz): δ= 163.37 (d, *J^1^* = 242.8 Hz), 162.23, 153.56, 153.20, 150.35, 139.97 (d, *J^3^* = 11.0 Hz), 139.00, 130.24 (d, *J^3^* = 9.5 Hz), 128.51, 128.50, 128.16, 124.20, 113.87 (d, *J^4^* = 1.4 Hz), 111.25, 110.03 (d, *J^2^* = 21.3 Hz), 106.04 (d, *J^2^* = 26.6 Hz), 72.33, 53.67, 29.25, 26.58, 19.38

HRMS (ESI+): m/z

[M + H]+ calcd for C23H22FN2O3: 393.1614 ; found: 393.1631

**
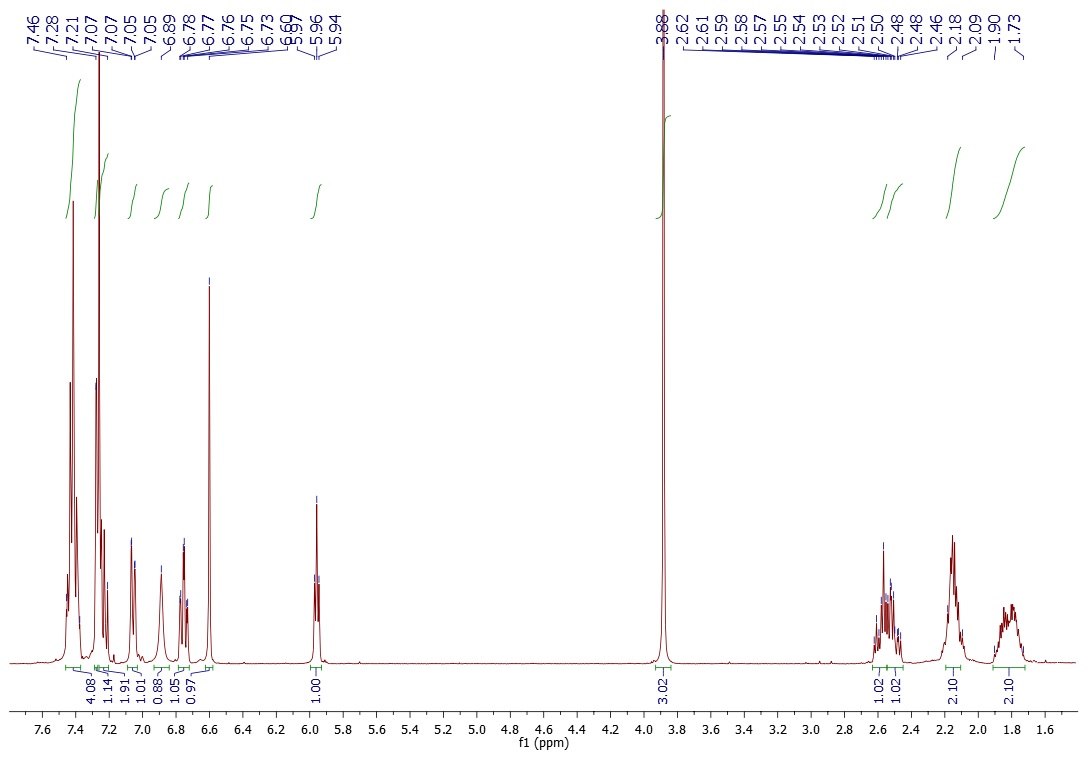
**

**Figure S33. ^1^H NMR of (2-methoxy-4-phenyl-5,6,7,8-tetrahydroquinolin-8-yl) N-(3-fluorophenyl)carbamate**

**
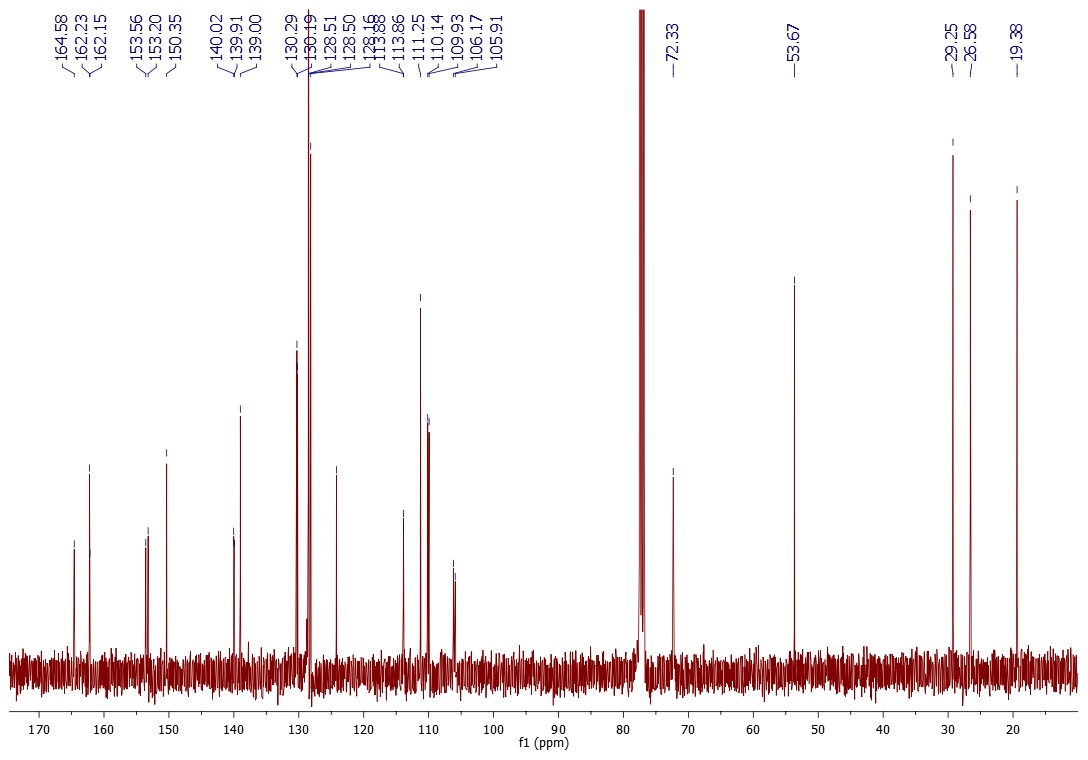
**

**Figure S34. ^13^C NMR of (2-methoxy-4-phenyl-5,6,7,8-tetrahydroquinolin-8-yl) N-(3-fluorophenyl)carbamate**

**18. (2-methoxy-4-phenyl-5,6,7,8-tetrahydroquinolin-8-yl) N-(4-chlorophenyl)carbamate (19e)**


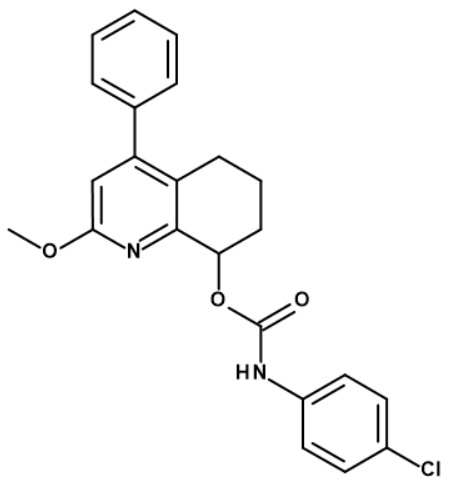


White cristals, yield: 21%, mp: 144 – 146^o^C

^1^H NMR (CDCl_3_, 400 MHz): δ= 7.45 – 7.38 (m, 5 H), 7.29 – 7.25 (m, 4 H), 6.84 (s, 1 H), 6.60 (s, 1 H), 5.95 (t, *J* = 5.2, 1 H), 3.88 (s, 3 H), 2.58 (dt, *J^2^* = 16.9 Hz, *J^3^* = 5.7 Hz, 1 H), 2.50 (ddd, *J^2^* = 16.6 Hz, *J^3^* = 8.0 Hz, *J^3^* = 5.6 Hz, 1 H), 2.21 – 2.08 (m, 2 H), 1.90 – 1.72 (m, 2 H)

^13^C NMR (CDCl_3_, 100 MHz): δ= 162.23, 153.56, 153.36, 150.41, 139, 136.95, 129.18, 128.51, 128.49, 128.35, 128.17, 124.20, 119.86, 111.21, 72.29, 53.66, 29.26, 26.58, 19.38

HRMS (ESI+): m/z

[M + H]+ calcd for C23H22ClN2O3: 409.1318 ; found: 409.1332

**
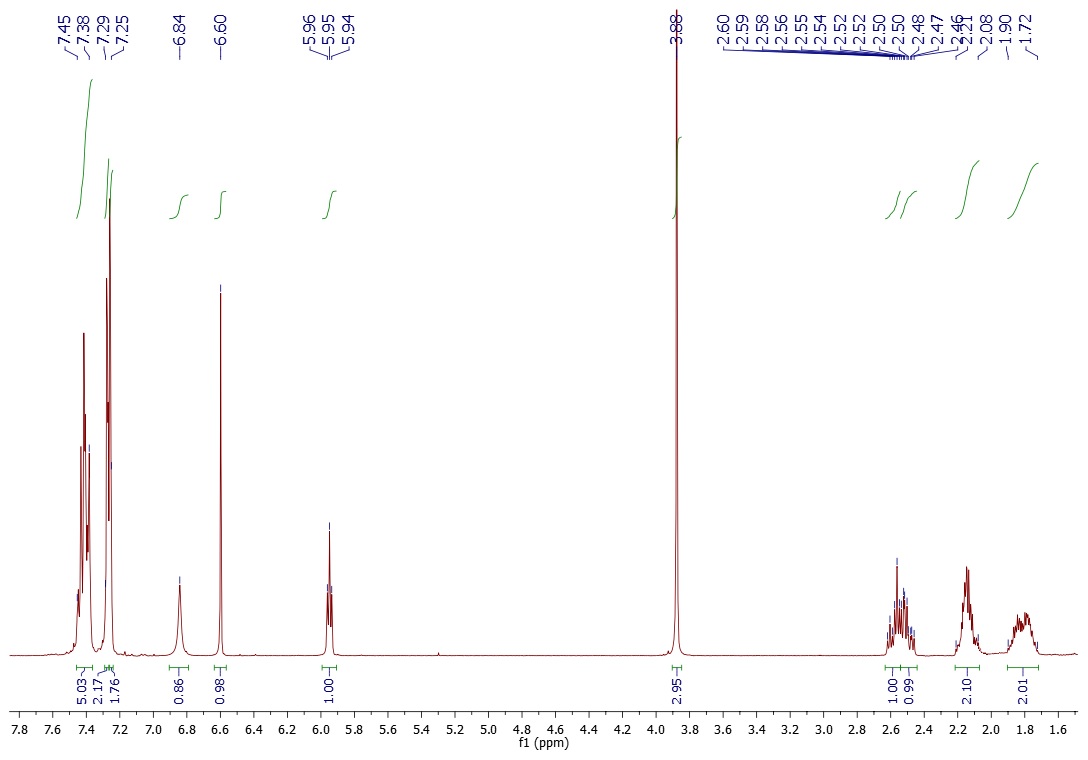
**

**Figure 35. ^1^H NMR of (2-methoxy-4-phenyl-5,6,7,8-tetrahydroquinolin-8-yl) N-(4-chlorophenyl)carbamate**

**
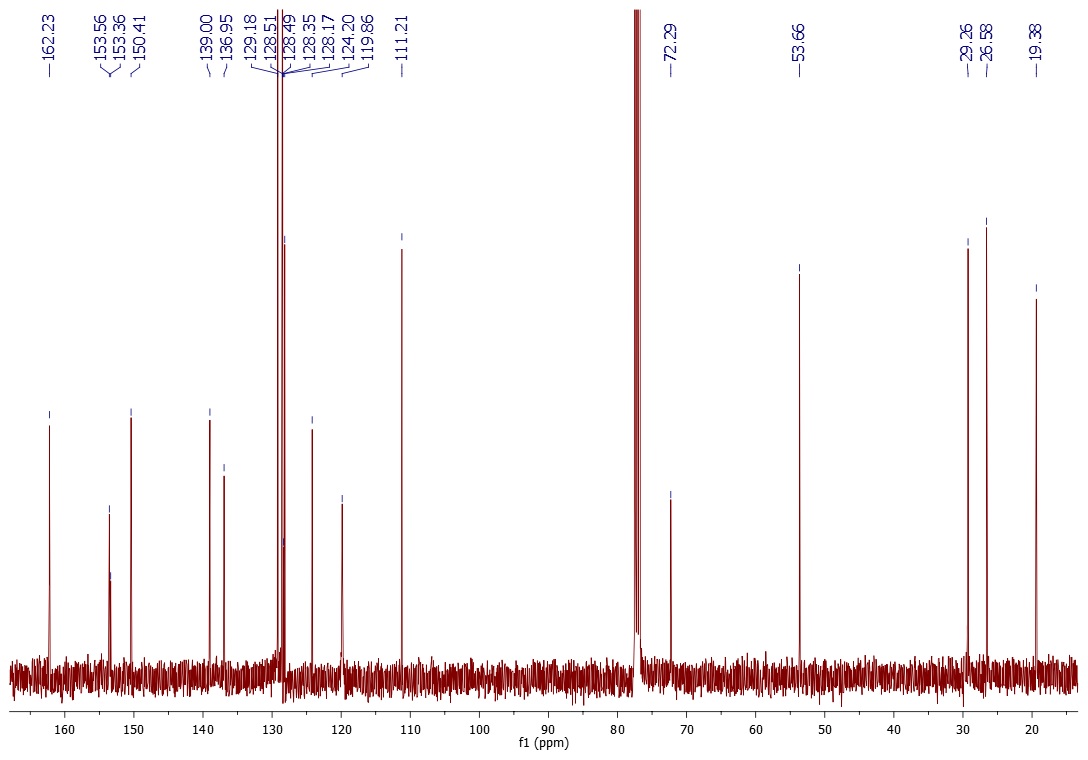
**

**Figure S36. ^13^C NMR of (2-methoxy-4-phenyl-5,6,7,8-tetrahydroquinolin-8-yl) N-(4-chlorophenyl)carbamate**

**19. (2-oxo-4-phenyl-5,6,7,8-tetrahydroquinolin-8-yl) N-phenylcarbamate (20a)**


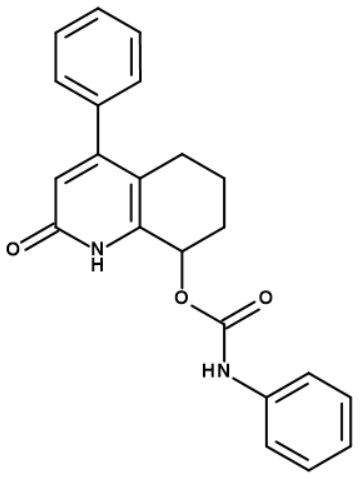


White cristals, yield: 80%, mp: 144 – 146^o^C

^1^H NMR (CDCl_3_, 400 MHz): δ= 7.45 – 7.40 (m, 5 H), 7.33 – 7.27 (m, 4 H), 7.06 (t, *J* = 7.4, 1 H), 6.88 (s, 1 H), 6.61 (s, 1 H), 5.96 (t, J = 5.3, 1 H), 3.90 (s, 3 H), 2.59 (dt, *J^2^* = 17.0 Hz, *J^3^* = 5.7 Hz, 1 H), 2.51 (ddd, *J^2^* = 16.5 Hz, *J^3^* = 8.0 Hz, *J^3^* = 5.6 Hz, 1 H), 2.22 – 2.11 (m, 2 H), 1.91 – 1.74 (m, 2 H)

^13^C NMR (CDCl_3_, 100 MHz): δ= 162.10, 153.93, 153.43, 150.43, 138.93, 138.31, 129.19, 128.54, 128.49, 128.25, 124.33, 123.40, 118.68, 111.13, 71.93, 53.93, 29.24, 26.59, 19.37

HRMS (ESI+): m/z

[M + H3O]+ calcd for C22H23N2O4: 379.1658 ; found: 379.1243

**
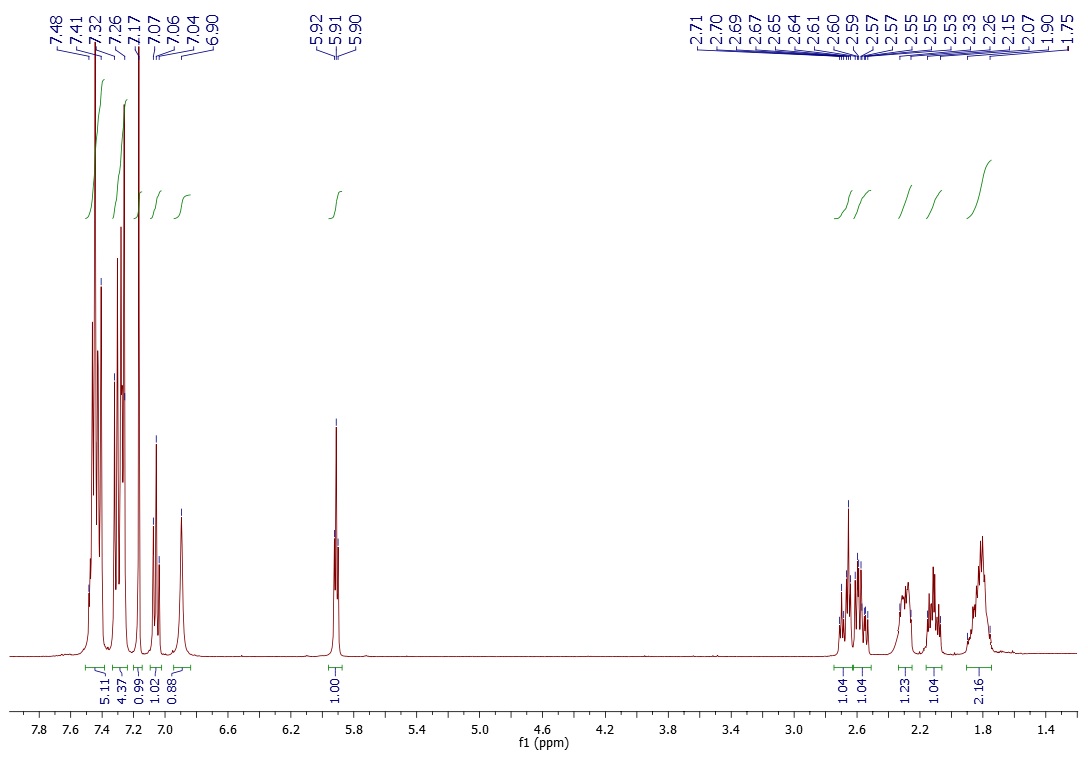
**

**Figure S37. ^1^H NMR of (2-oxo-4-phenyl-5,6,7,8-tetrahydroquinolin-8-yl) N-phenylcarbamate**

**
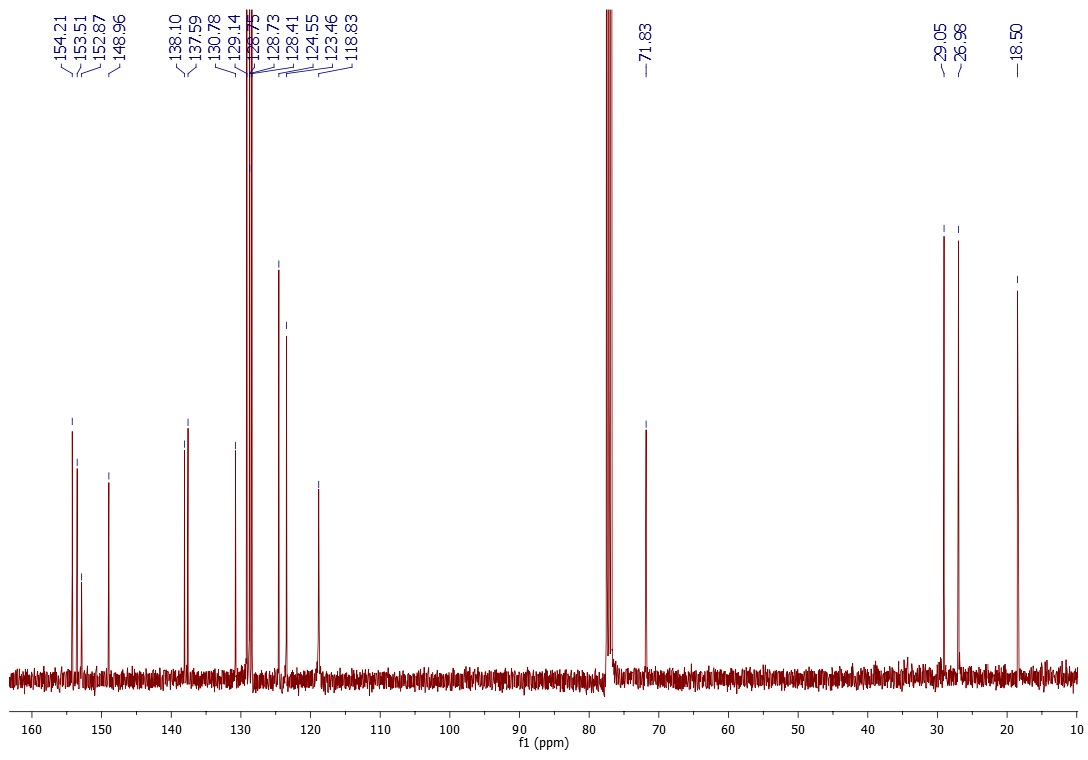
**

**Figure S38. ^13^C NMR of (2-oxo-4-phenyl-5,6,7,8-tetrahydroquinolin-8-yl) N-phenylcarbamate**

**20. (2-oxo-4-phenyl-5,6,7,8-tetrahydroquinolin-8-yl) N-(4-nitrophenyl)carbamate (20b)**


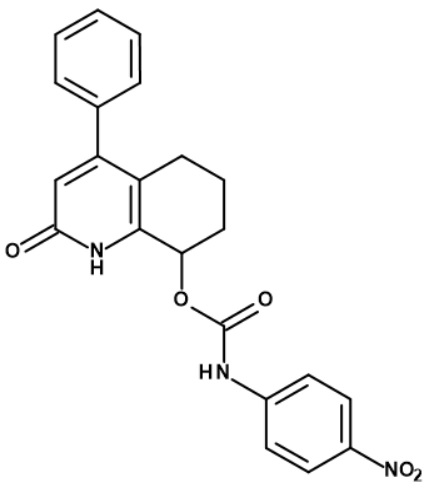


Bright yellow cristals, yield: 70%, mp: 225 – 227^o^C

^1^H NMR (CDCl_3_, 400 MHz): δ= 8.19 (d, *J* = 9.1 Hz, 2 H), 7.58 (d, *J* = 9.1 Hz, 2 H), 7.46 – 7.43 (m, 3 H), 7.25 – 7.24 (m, 3 H), 7.18 (s, 1 H), 5.92 (t, *J* = 4.6, 1 H), 2.67 (dt, *J^2^* = 17.4 Hz, *J^3^* = 5.2 Hz, 1 H), 2.63 – 2.55 (m, 1 H), 2.29 – 2.22 (m, 1 H), 2.17 – 2.09 (m, 1 H), 1.83 – 1.78 (m, 2 H)

^13^C NMR (CDCl_3_, 100 MHz): δ= 153.75, 153.71, 152.38, 149.02, 144.40, 143.07, 137.33, 130.91, 128.91, 128.85, 128.39, 125.34, 124.70, 117.90, 72.24, 29.08, 26.98, 18.50

HRMS (ESI+): m/z

[M + H3O]+ calcd for C22H22N3O6: 424.1509 ; found: 424.1559

**
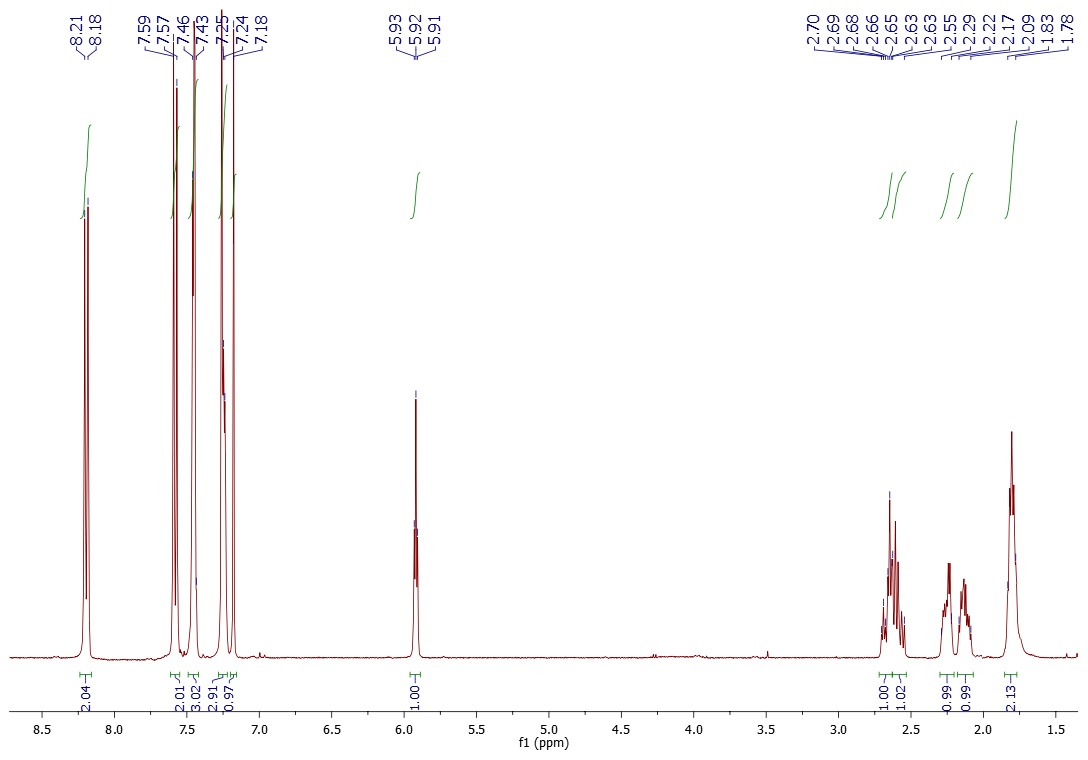
**

**
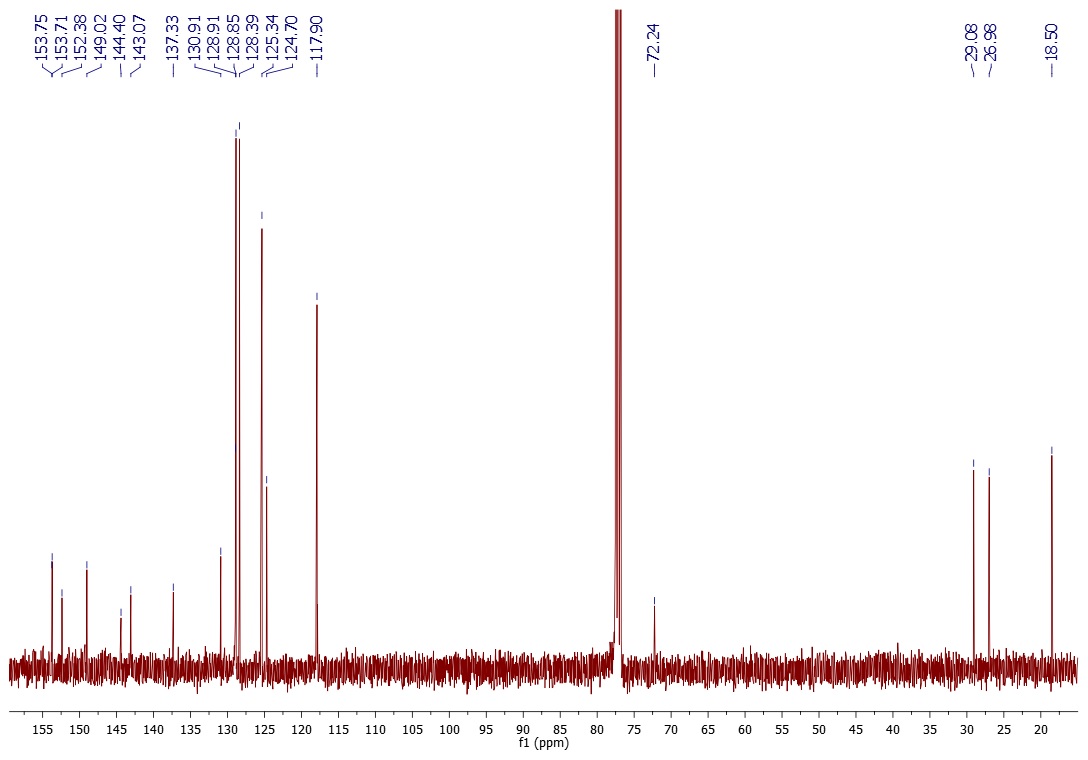
Figure S39. ^1^H NMR of (2-oxo-4-phenyl-5,6,7,8-tetrahydroquinolin-8-yl) N-(4-nitrophenyl)carbamate**

**Figure S40. ^13^C NMR of (2-oxo-4-phenyl-5,6,7,8-tetrahydroquinolin-8-yl) N-(4-nitrophenyl)carbamate**

**21. (2-oxo-4-phenyl-5,6,7,8-tetrahydroquinolin-8-yl) N-(4-fluorophenyl)carbamate (20c)**


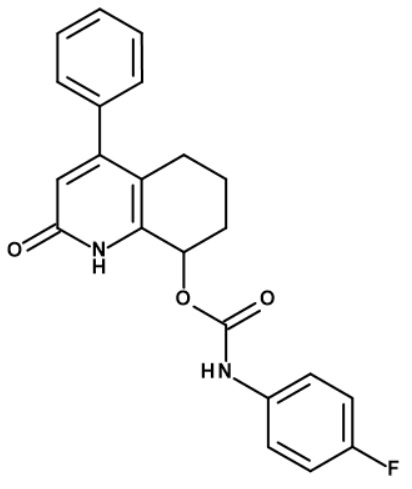


White amorphous powder, yield: 64%,

^1^H NMR (CDCl_3_, 400 MHz): δ= 7.48 – 7.43 (m, 3 H), 7.39 – 7.36 (m, 2 H), 7.27 – 7.24 (m, 2 H), 7.16 (s, 1 H), 7.03 – 6.97 (m, 3 H), 5.89 (t, *J* = 4.7, 1 H), 2.67 (dt, *J^2^* = 17.4 Hz, *J^3^* = 5.2 Hz, 1 H), 2.57 (ddd, *J^2^* = 16.8 Hz, *J^3^* = 8.4 Hz, *J^3^* = 6.2 Hz, 1 H), 2.30 – 2.23 (m, 1 H), 2.14 – 2.06 (m, 1 H), 1.84 – 1.77 (m, 2 H)

^13^C NMR (CDCl_3_, 100 MHz): δ= 159.05 (d, *J^1^* = 240,5 Hz), 154.20, 153.51, 153.12, 148.97, 137.55, 134.15 (d, *J^4^* = 2.6 Hz), 130.75, 128.77, 128.40, 124.53, 120.61 (d, *J^3^* = 3.1 Hz), 115.72 (d, *J^2^* = 22.4 Hz), 71.86, 29.08, 26.99, 18.52

HRMS (ESI+): m/z

[M + H3O]+ calcd for C22H22FN2O4: 397.1564 ; found: 397.1134

**
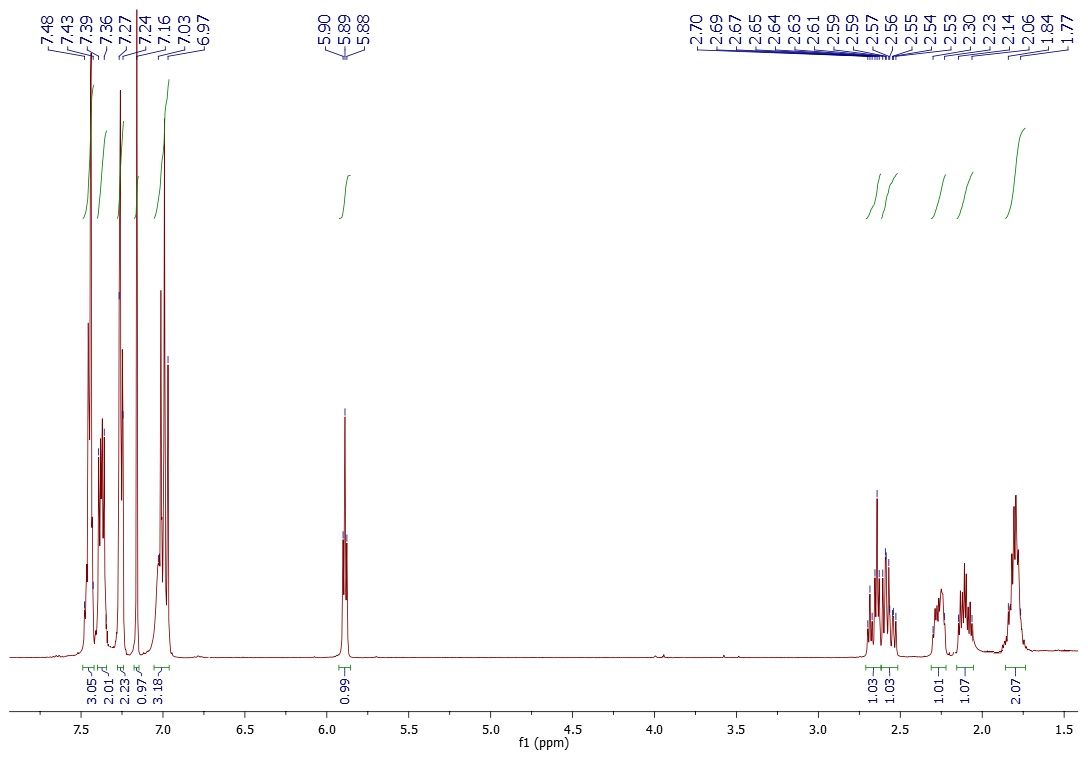
**

**Figure S41. ^1^H NMR of (2-oxo-4-phenyl-5,6,7,8-tetrahydroquinolin-8-yl) N-(4-fluorophenyl)carbamate**

**
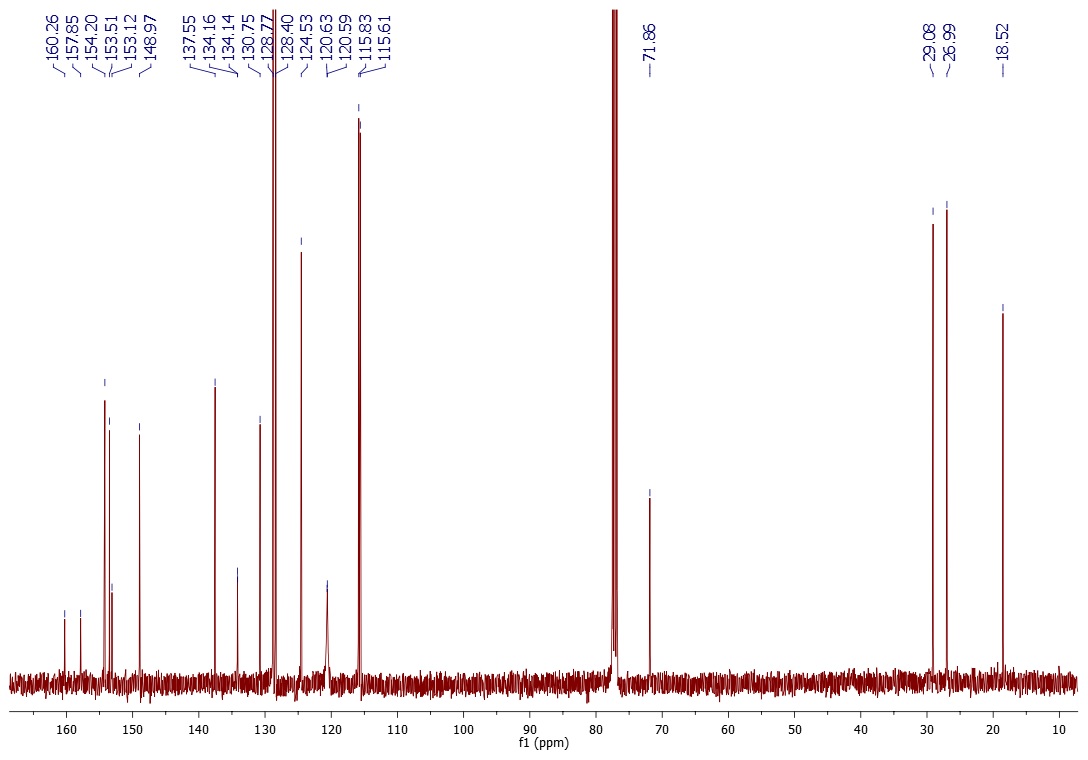
**

**Figure S42. ^13^C NMR of (2-oxo-4-phenyl-5,6,7,8-tetrahydroquinolin-8-yl) N-(4-fluorophenyl)carbamate**

**22. (2-oxo-4-phenyl-5,6,7,8-tetrahydroquinolin-8-yl) N-(3-fluorophenyl)carbamate (20d)**


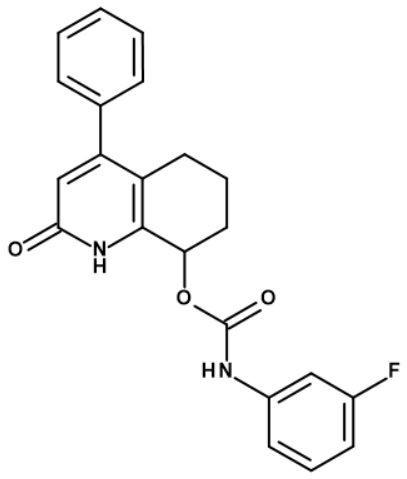


White amorphous powder, yield: 63%

^1^H NMR (CDCl_3_, 400 MHz): δ= 7.47 – 7.43 (m, 3 H), 7.35 (d, *J* = 11.0 Hz, 1 H), 7.27 – 7.20 (m, 3 H), 7.17 (s, 1 H), 7.16 (s, 1 H), 7.06 (dd, *J^5^* = 8.0 Hz, *J^5^* = 1.2 Hz, 1 H), 6.74 (td, *J^3^* = 8.3 Hz, *J* = 2.3 Hz, 1 H), 5.90 (t, *J* = 4.7 Hz, 1 H), 2.67 (dt, *J^2^* = 17.4 Hz, *J^3^* = 5.2 Hz, 1 H), 2.57 (ddd, *J^2^* = 17.3 Hz, *J^3^* = 8.0 Hz, *J^3^* = 6.6 Hz, 1 H), 2.30 – 2.23 (m, 1 H), 2.15 – 2.07 (m, 1 H), 1.84 – 1.79 (m, 2 H)

^13^C NMR (CDCl_3_, 100 MHz): δ= 163.31 (d, *J^1^* = 242.7 Hz), 154.03, 153.61, 152.65, 148.95, 139.83 (d, *J^3^* = 11.0 Hz), 137.50, 130.83, 130.18 (d, *J^3^* = 9.5 Hz), 128.79, 128.41, 124.61, 114.04 (d, *J^4^* = 1.4 Hz), 110.07 (d, *J^2^* = 21.2 Hz), 106.17 (d, *J^2^* = 26.3 Hz), 71.93, 29.05, 26.98, 18.50

HRMS (ESI+): m/z

[M + H3O]+ calcd for C22H22FN2O4: 397.1564 ; found: 397.1126

**
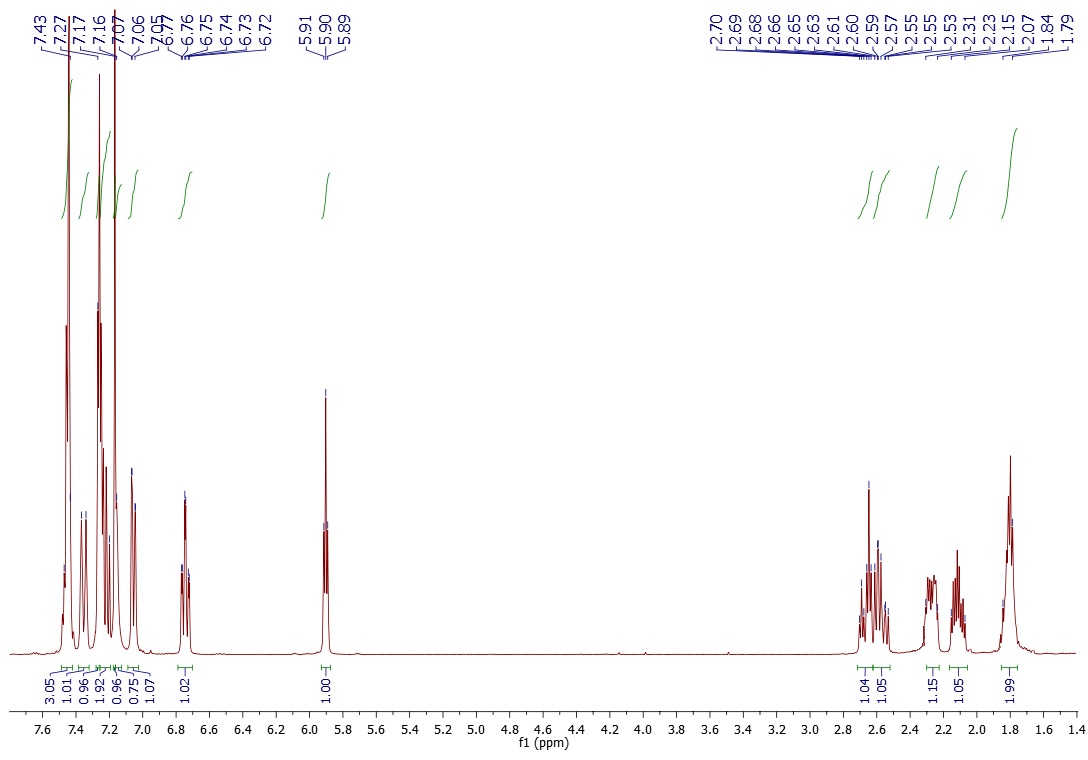
**

**Figure S43. ^1^H NMR of (2-oxo-4-phenyl-5,6,7,8-tetrahydroquinolin-8-yl) N-(3-fluorophenyl)carbamate**

**
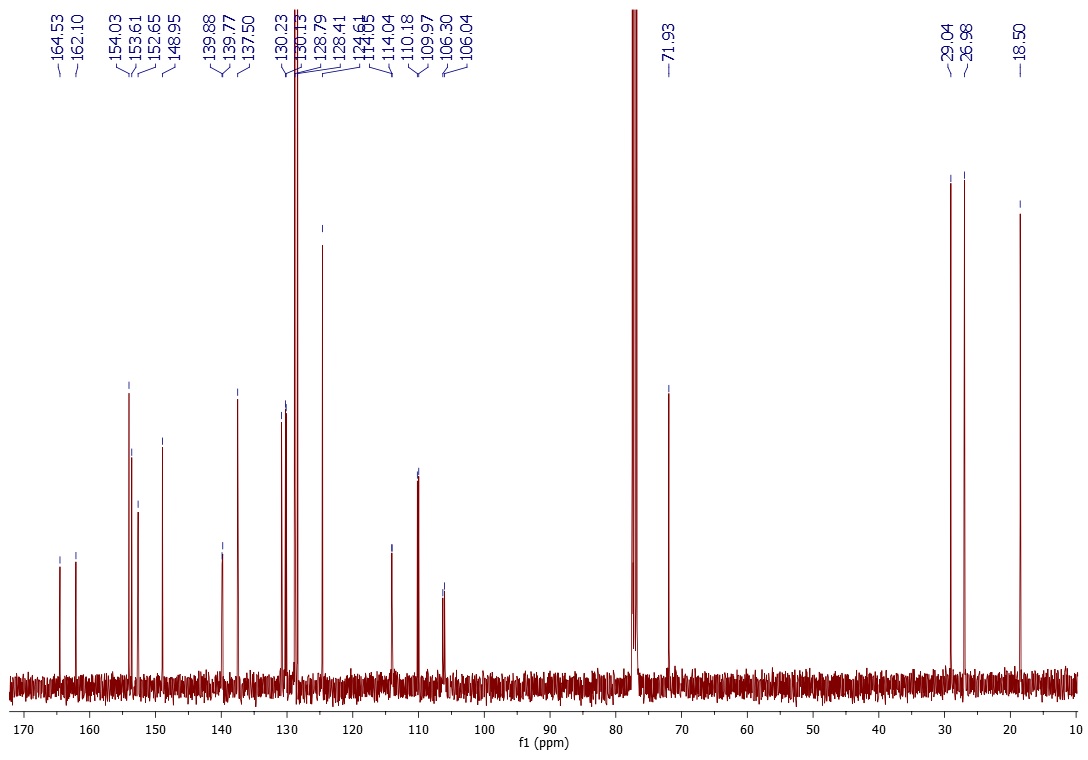
**

**Figure S44. ^13^C NMR of (2-oxo-4-phenyl-5,6,7,8-tetrahydroquinolin-8-yl) N-(3-fluorophenyl)carbamate**

**23. (2-oxo-4-phenyl-5,6,7,8-tetrahydroquinolin-8-yl) N-(4-chlorophenyl)carbamate (20e)**


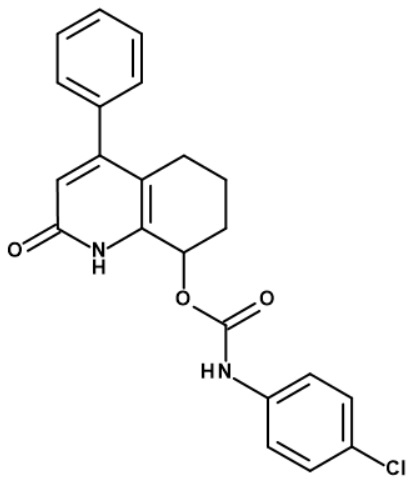


White amorphous powder, yield: 69%

^1^H NMR (CDCl_3_, 400 MHz): δ= 7.47 – 7.43 (m, 3 H), 7.37 (d, *J* = 8.8 Hz, 2 H), 7.25 – 7.24 (m, 3 H), 7.18 (s, 1 H), 7.16 (s, 1 H), 5.89 (t, *J* = 4.7, 1 H), 2.66 (dt, *J^2^* = 17.4 Hz, *J^3^* = 5.2 Hz, 1 H), 2.60 – 2.52 (m, 1 H), 2.29 – 2.22 (m, 1 H), 2.14 – 2.08 (m, 1 H), 1.84 – 1.76 (m, 2 H)

^13^C NMR (CDCl_3_, 100 MHz): δ= 154.11, 153.53, 152.84, 148.97, 137.50, 136.85, 130.77, 129.10, 128.77, 128.40, 128.35, 124.54, 120.04, 71.87, 29.09, 26.99, 18.51

HRMS (ESI+): m/z

[M + H3O]+ calcd for C22H22ClN2O4: 413.1268 ; found: 413.0818

**
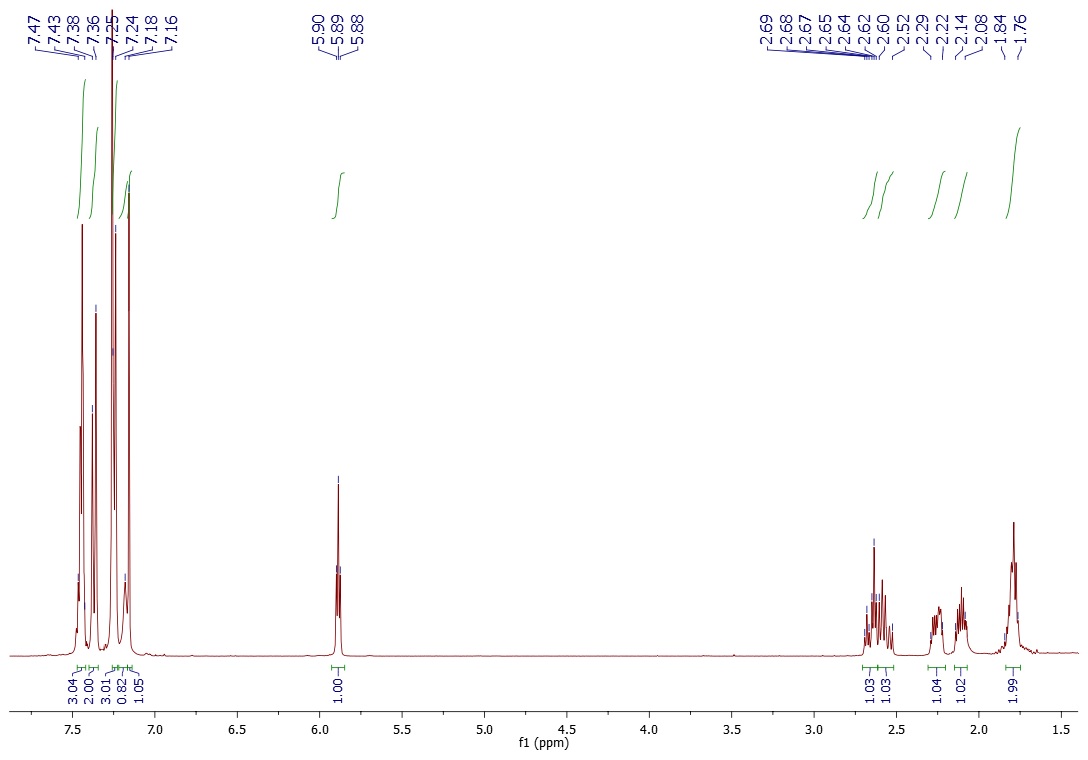
**

**Figure S45. ^1^H NMR of (2-oxo-4-phenyl-5,6,7,8-tetrahydroquinolin-8-yl) N-(4-chlorophenyl)carbamate**

**
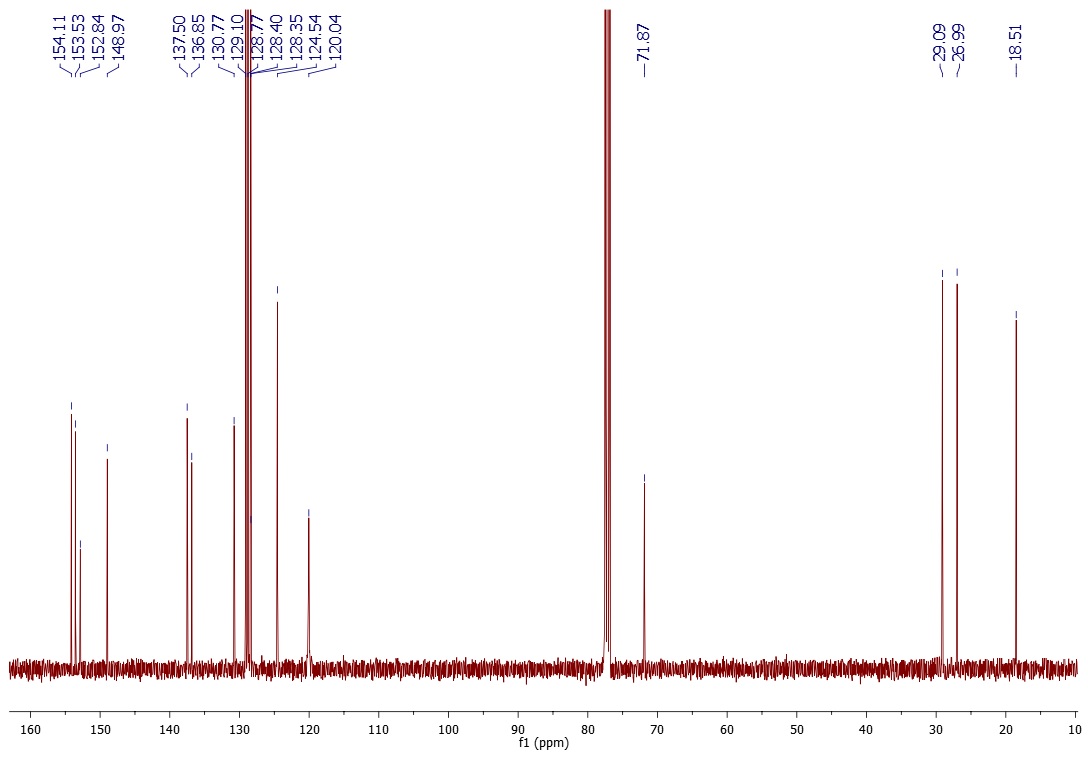
**

**Figure S46. ^13^C NMR of (2-oxo-4-phenyl-5,6,7,8-tetrahydroquinolin-8-yl) N-(4-chlorophenyl)carbamate**

**24. 2-chloro-4-phenyl-6,7-dihydro-5H-quinolin-8-one (21)**

A solution of oxalyl chloride (0.36 ml, 4,23 mmol) in 30 ml DCM was placed in three neck round-bottomed flask filled with Ar. The solution was cooled to -78^o^C, DMSO (0.6 ml, 8,4 mmol) was added dropwise and mixture was left for 20 min. In the next step a solution of **16** (0.785 g, 3 mmol) in 50 ml DCM was added carefully to not increase the temperature above -65^o^C and left for another 15 min. In last step NEt_3_ (2.5 ml, 18 mmol) was added dropwise and reaction mixture was left overnight to warm up to RT. Solvent was evaporated, residue was dissolved in AcOEt and washed with 1M HCl, 5% NaHCO_3_, H_2_O i brine. Organic layer was dried with anhydrous MgSO_4_. Crude product was isolated by flash column chromatography (ethyl acetate : hexane 1:3). Title compound (0.66 g, 2.55 mmol, 85%) was obtained as white cristals.


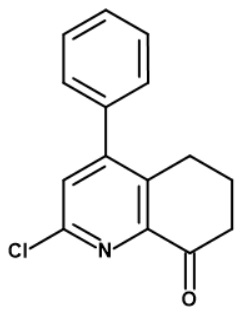
White cristals, yield: 85%, mp: 135 – 137^o^C

^1^H NMR (CDCl_3_, 400 MHz): δ= 7.52 – 7.46 (m, 3 H), 7.38 (s, 1 H), 7.32 – 7.3 (m, 2 H), 2.88 (t, *J* = 5.9 Hz, 2H), 2.80 (t, *J* = 6.3, 2 H), 2.08 (qu, *J* = 6.4 Hz, 2 H)

^13^C NMR (CDCl_3_, 100 MHz): δ= 195.28, 154.04, 150.8, 148.61, 137.65, 136.64, 129.21, 128.94, 128.53, 128.25, 39.39, 27.33, 22.66

HRMS (ESI+): m/z [M + H]+ calcd for C15H13ClNO: 258.0686 ; found: 258.0705

**
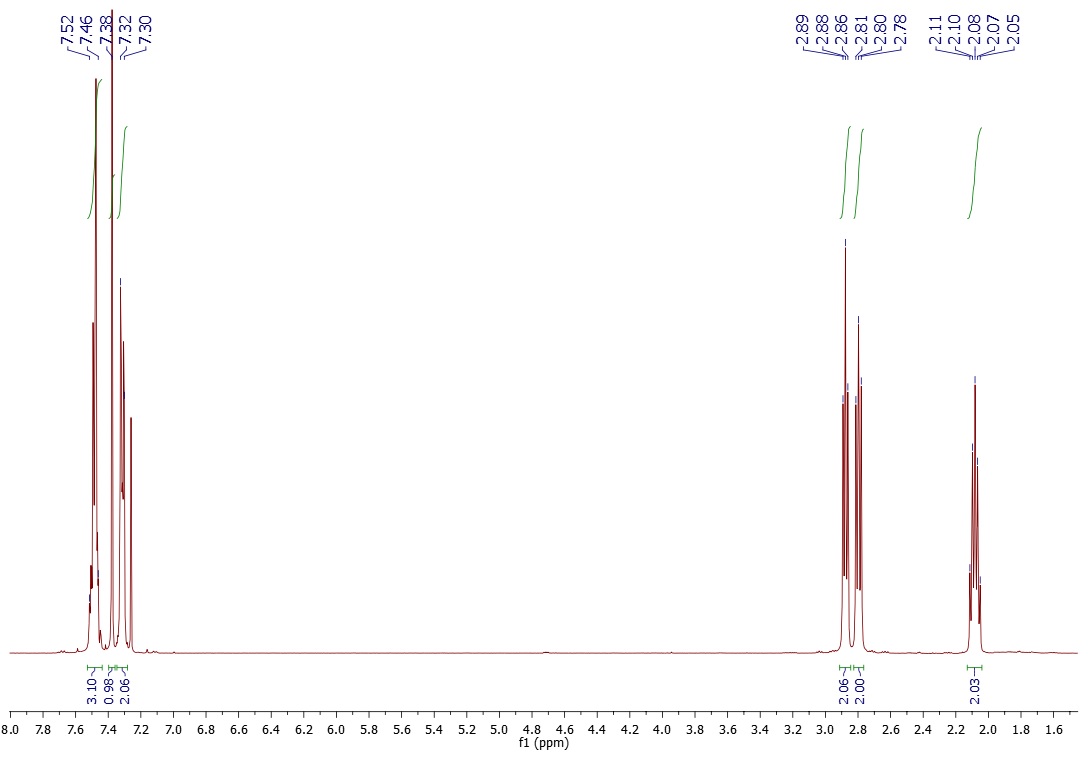
**

**Figure S47. ^1^H NMR of 2-chloro-4-phenyl-6,7-dihydro-5H-quinolin-8-one**


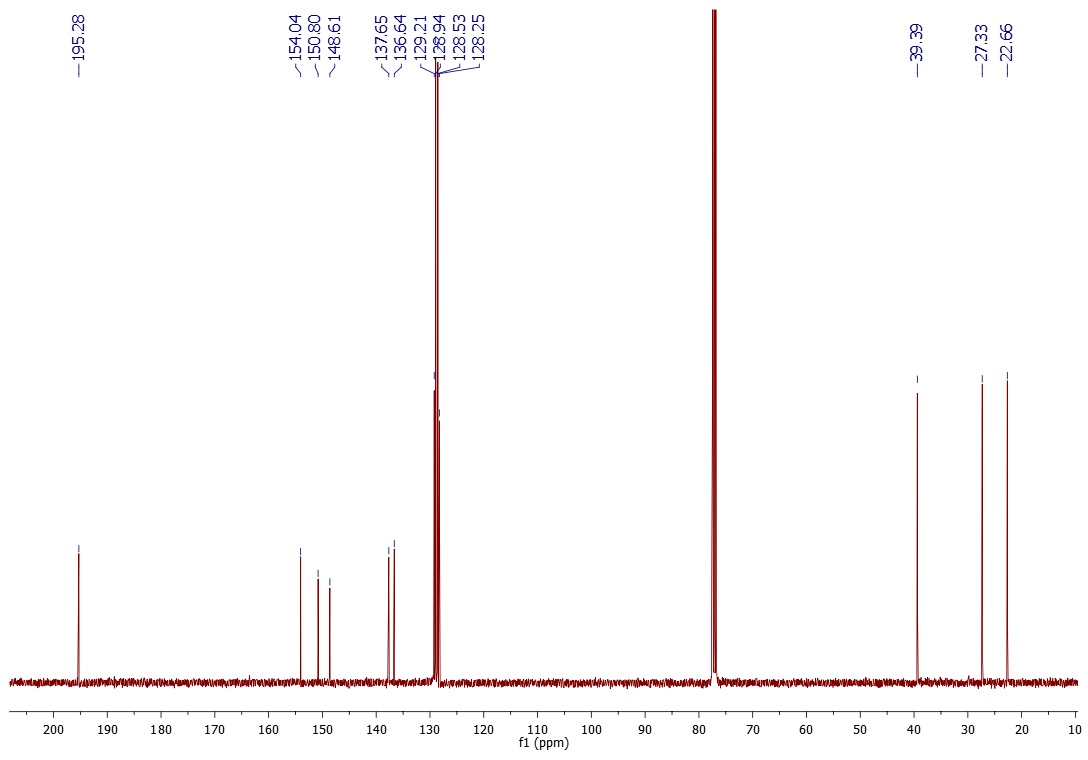


**Figure S48. ^13^C NMR of 2-chloro-4-phenyl-6,7-dihydro-5H-quinolin-8-one**

**25. 2-methoxy-4-phenyl-6,7-dihydro-5H-quinolin-8-one (22)**

Title compounds were synthesised according to procedure of **11** by using compound **23** as substrate. Crude product was purified by flash column chromatography (methylene chloride : methanol 600:1). Title compound (0.019 g, 0.08 mmol, 73%) was obtained as white cristals.


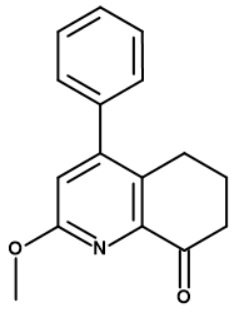


White cristals, yield: 73%, mp: 127 – 129^o^C

^1^H NMR (CDCl_3_, 400 MHz): δ= 7.49 – 7.43 (m, 3 H), 7.32 -7.30 (m, 2 H), 6.85 (s, 1 H), 4.06 (s, 3 H), 2.81 – 2.74 (m, 4 H), 2.05 (qu, *J* = 6.3 Hz, 2 H)

^13^C NMR (CDCl_3_, 100 MHz): δ= 195.28, 154.04, 150.85, 148.63, 137.64, 136.66, 128.95, 128.55, 128.27, 116.46, 53.83, 39.41, 27.34, 22.67

HRMS (ESI+): m/z

[M + H]+ calcd for C16H16NO2: 254.1181 ; found: 254.1190

**
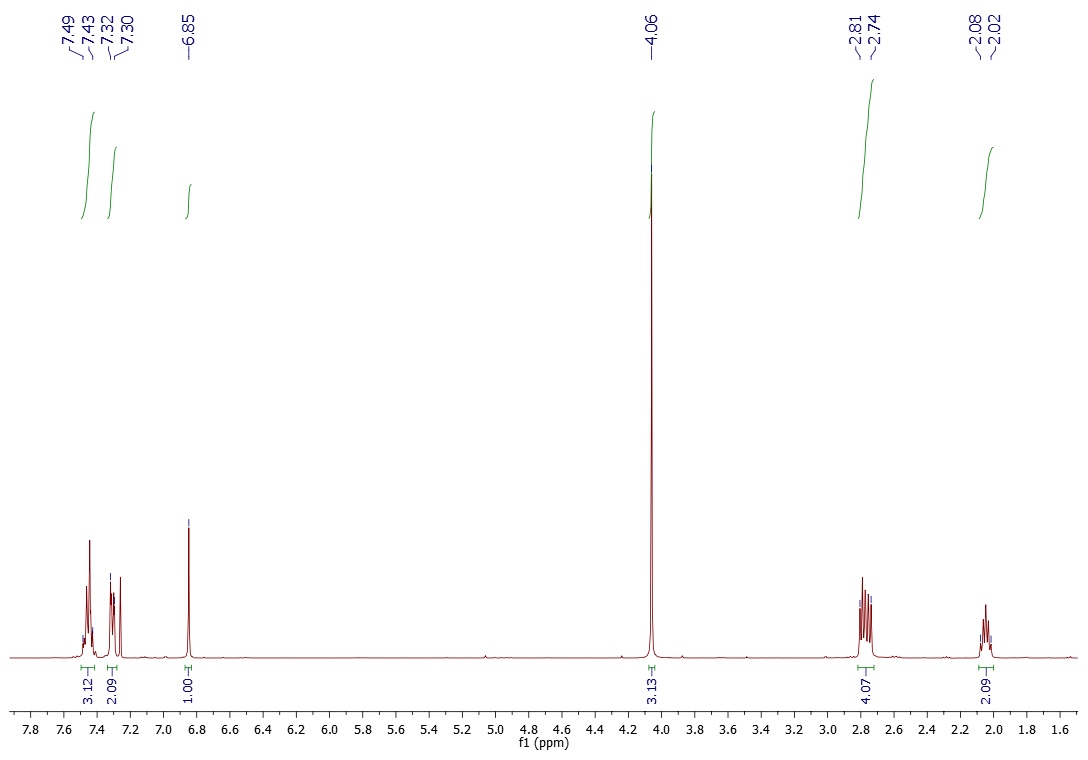
**

**Figure S49. ^1^H NMR of 2-methoxy-4-phenyl-6,7-dihydro-5H-quinolin-8-one**

**
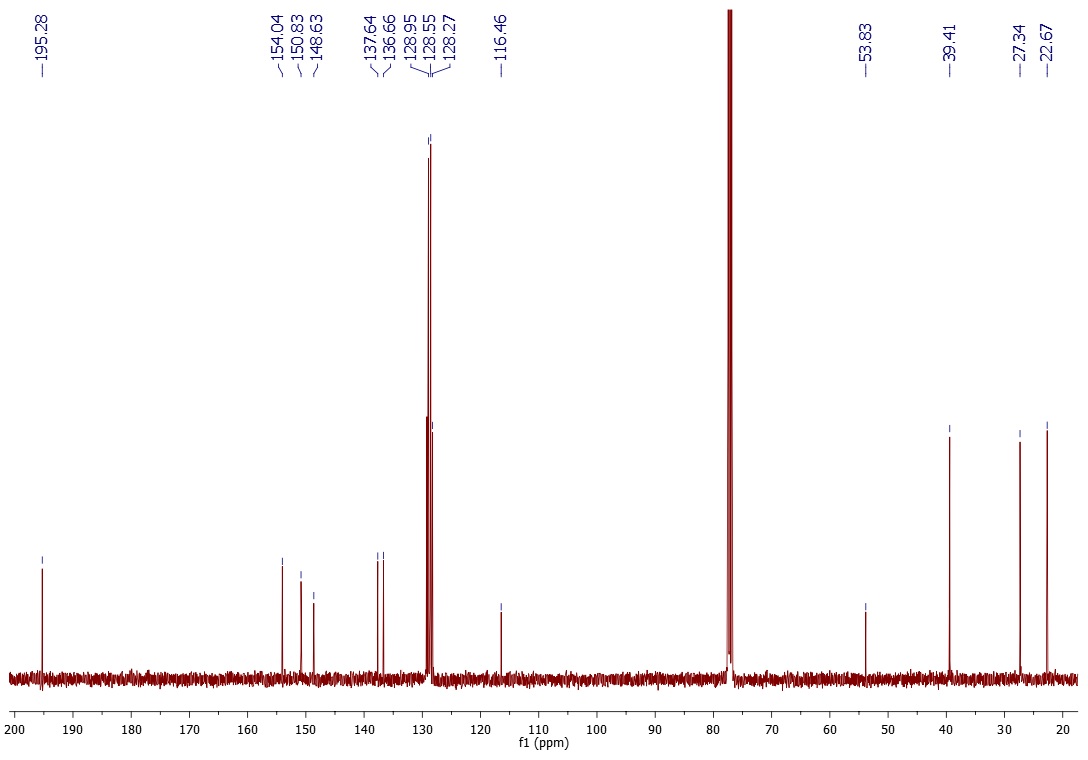
**

**Figure S50. ^13^C NMR of 2-methoxy-4-phenyl-6,7-dihydro-5H-quinolin-8-one**

**26. 4-phenyl-1,5,6,7-tetrahydroquinoline-2,8-dione (23)**

A solution of **17** (0.1 g, 0.4mmol) in 2 ml of 6M HCl was heated to reflux and left overnight. Reaction mixture was then diluted with 50 ml of water and washed with AcOEt. Organic layer was washed with 1M NaOH, brine and dried with anhydrous MgSO_4_. Crude product was purified by flash kolumn chromatography. Title compound (0.073 g, 0.3 mmol, 76%) was obtained as white cristals.


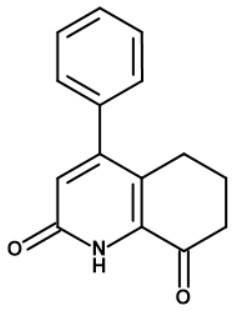
White cristals, yield: 76%, mp: 182 – 184^o^C

^1^H NMR (CDCl_3_, 400 MHz): δ= 7.48 – 7.43 (m, 3 H), 7.30 -7.28 (m, 2 H), 6.75 (s, 1 H), 2.67 (t, *J* = 6.6Hz, 2 H), 2.61 (t, *J* = 5.9 Hz, 2 H), 2.06 (qu, *J* = 6.3 Hz, 2 H)

^13^C NMR (CDCl_3_, 100 MHz): δ= 191.40, 160.87, 155.62, 136.68, 133.76, 129.21, 128.86, 128.12, 127.07, 126.21, 36.92, 25.70, 22.79

HRMS (ESI+): m/z

[M + H]+ calcd for C15H13NO2: 240.1025 ; found: 240.1024

**
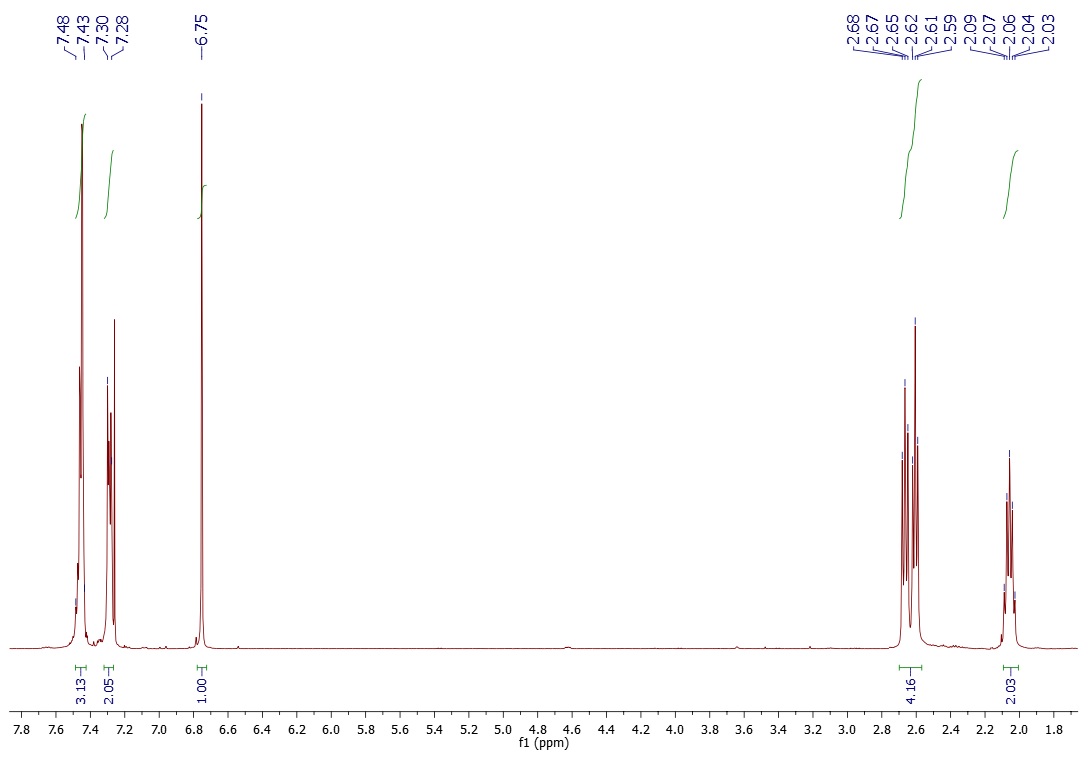
**

**Figure S51. ^1^H NMR of 4-phenyl-1,5,6,7-tetrahydroquinoline-2,8-dione**

**
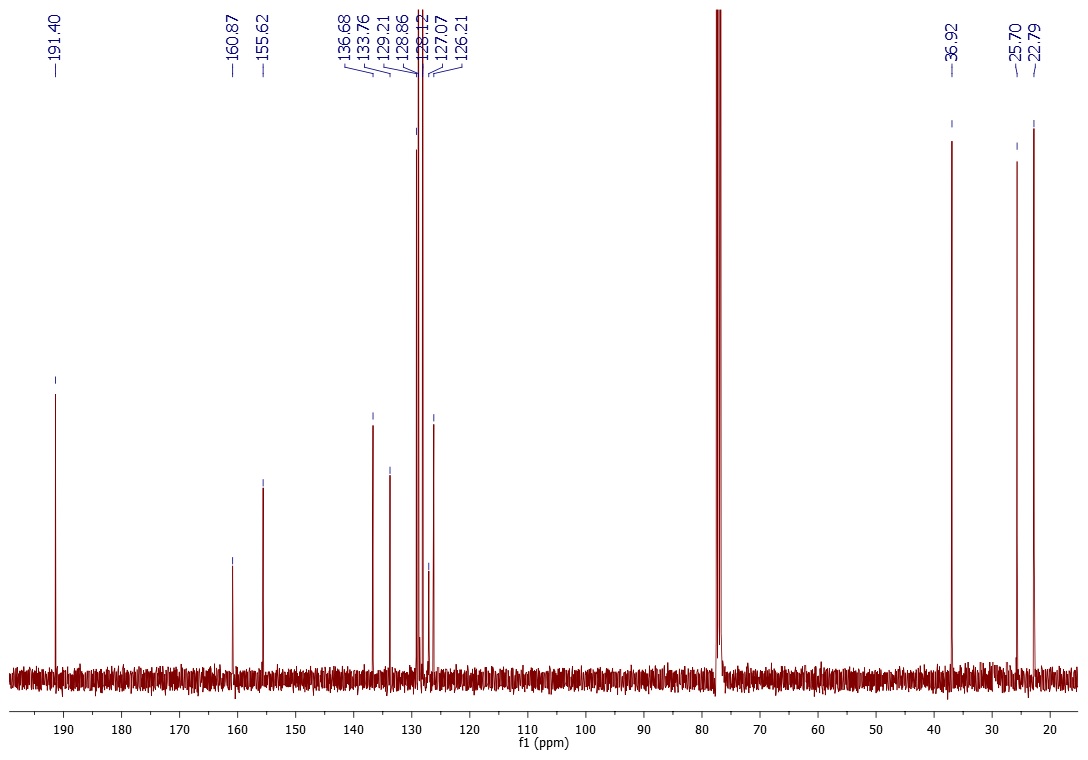
**

**Figure S52. ^13^C NMR of 4-phenyl-1,5,6,7-tetrahydroquinoline-2,8-dione**

**27. 8-hydroxy-4-phenyl-5,6,7,8-tetrahydro-1H-quinolin-2-one (24)**

A solution of **17** (0.05 g, 0.2 mmol) and NaI (0.05 g, 0.34 mmol) in 3 ml of glacial acetic acid was heated to reflux and left overnight. The solvent was evaporated. Residue was dilluted with AcOEt, washed with 1M NaOH, brine and dried with anhydrous MgSO_4_. Crude product was purified by flash column chromatography (ethyl acetate : hexane 1:5). Title compound (0.025 g, 0.1 mmol, 52%) was obtained as white cristals.

White cristals, yield: 50%, mp: 104-106^o^C

^1^H NMR (CDCl_3_, 400 MHz): δ= 7.48 – 7.42 (m, 3 H), 7.28 -7.25 (m, 2 H), 7.15 (s, 1 H), 5.93 (t, *J* = 4.6 Hz, 1 H), 2.67 (dt, *J^2^* = 17.4 Hz, *J^3^* = 5.1 Hz, 1 H), 2.55 (ddd, *J^2^* = 17.1 Hz, *J^3^* = 9.1 Hz, *J^3^* = 5.9 Hz, 1 H), 2.21 – 2.15 (m, 2 H), 2.07 – 1.99 (m, 2H), 1.87 – 1.71 (m, 2 H)

^13^C NMR (CDCl_3_, 100 MHz): δ= 170.33, 154.13, 148.92, 137.65, 130.78, 128.74, 128.69, 128.44, 124.57, 71.08, 28.79, 26.97, 18.48

HRMS (ESI+): m/z

[M + H]+ calcd for C15H16NO2: 242.1181; found: 242.0734

**
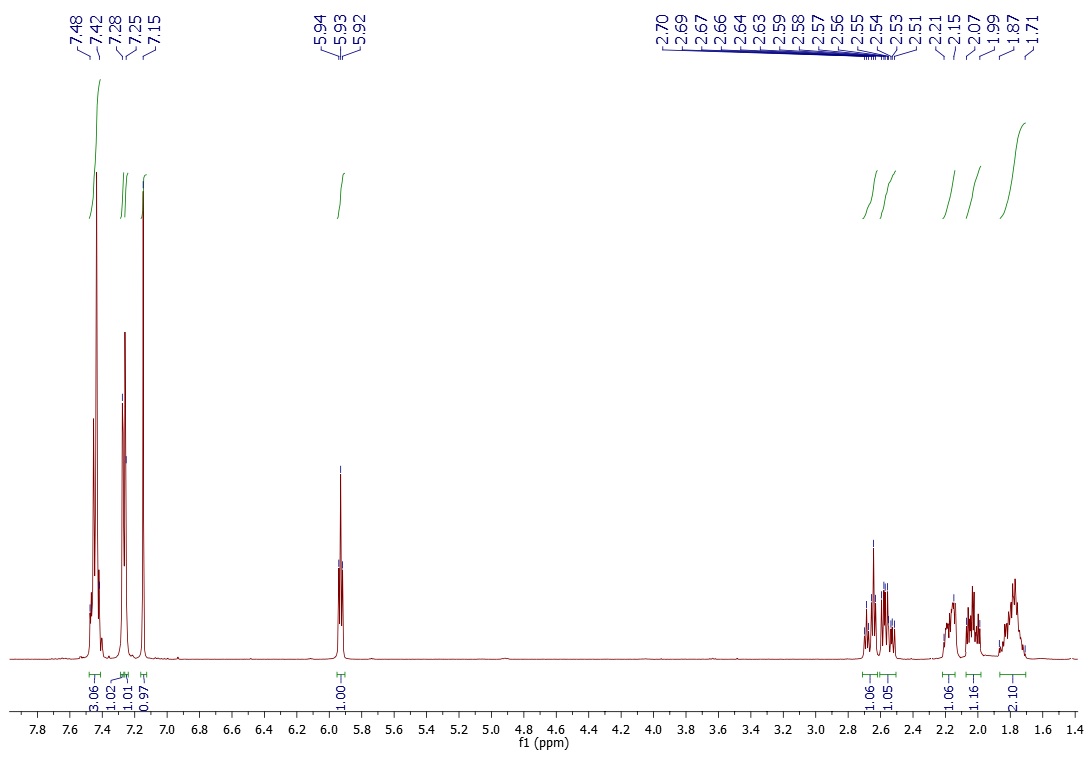
**

**Figure S53. ^1^H NMR of 8-hydroxy-4-phenyl-5,6,7,8-tetrahydro-1H-quinolin-2-one**

**
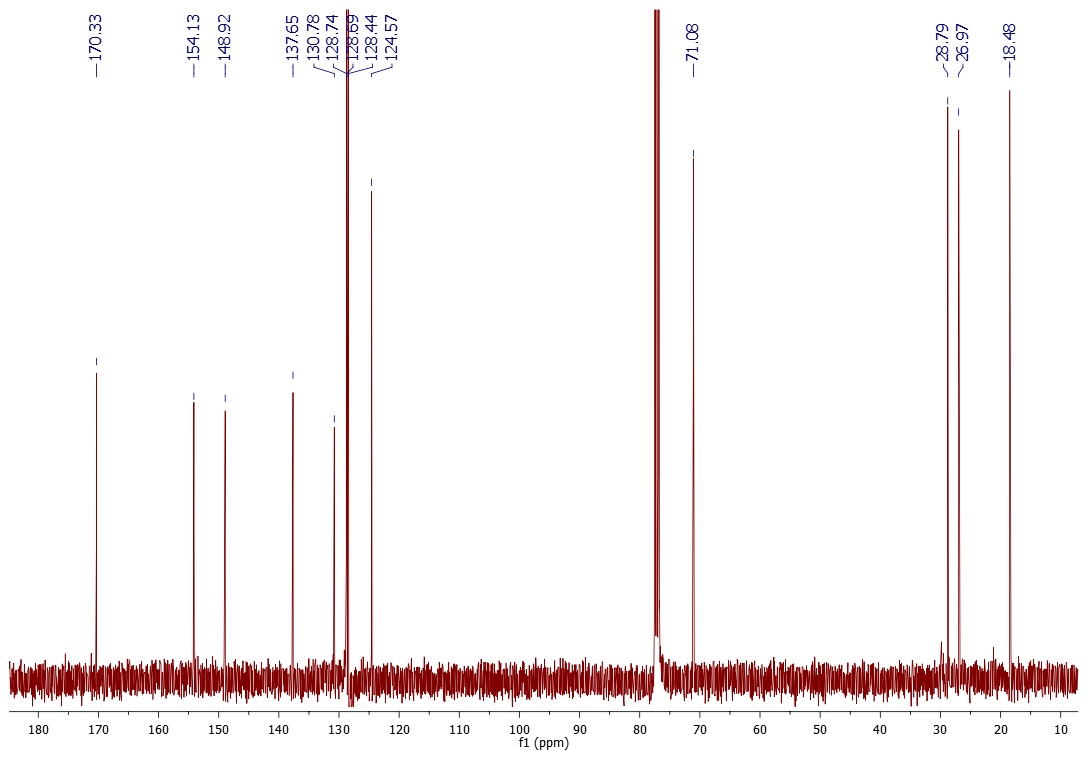
**

**Figure S54. ^13^C NMR of 8-hydroxy-4-phenyl-5,6,7,8-tetrahydro-1H-quinolin-2-one**

**BIOLOGICAL EVALUATION**


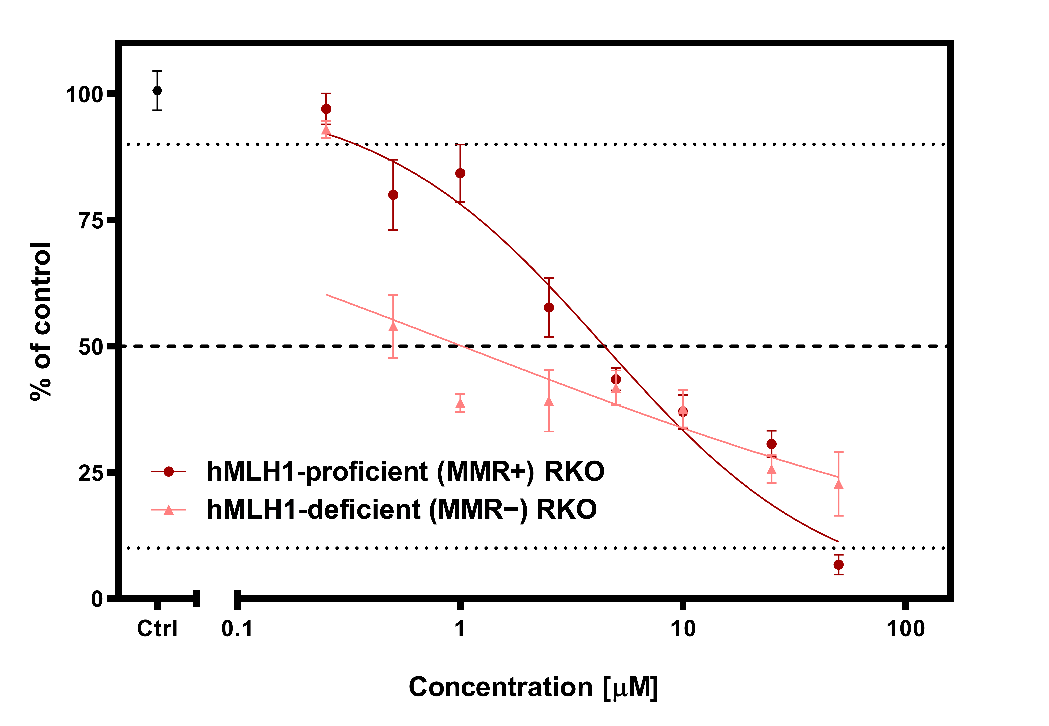


**Figure S55.** Dose–response curves of **20d** and **19b** in hMLH1-proficient and hMLH1-deficient RKO cell lines determined by the MTT assay. All data are presented as mean±SEM of three independent experiments in triplicates.


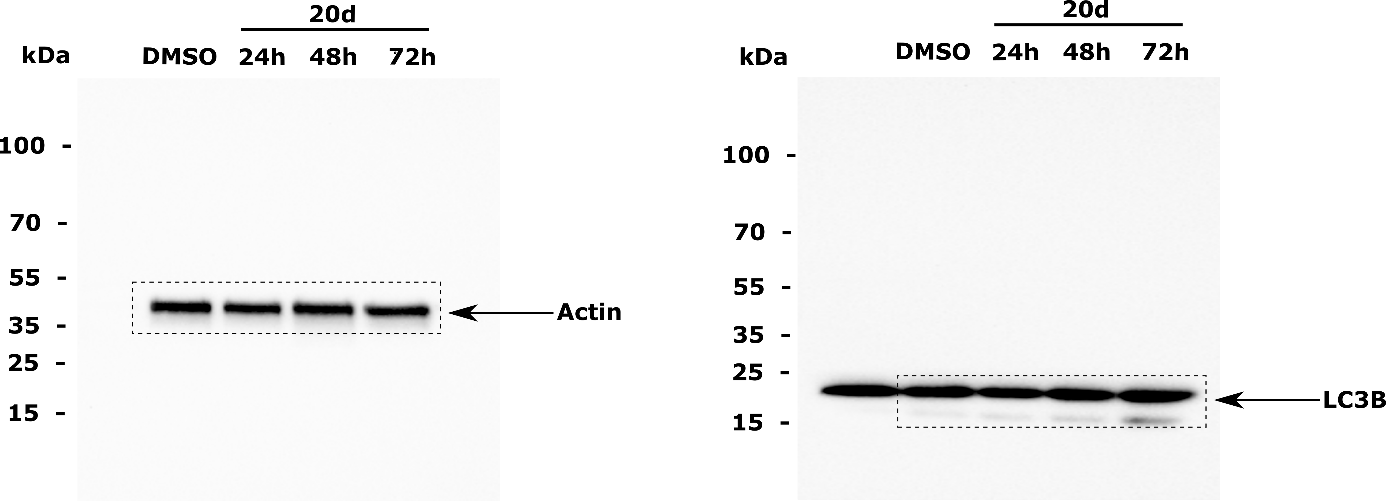


**Figure S56.** Full length western blot presented in **Figure 8** of the main article. Dotted boxes denote the cropped regions of the blots.


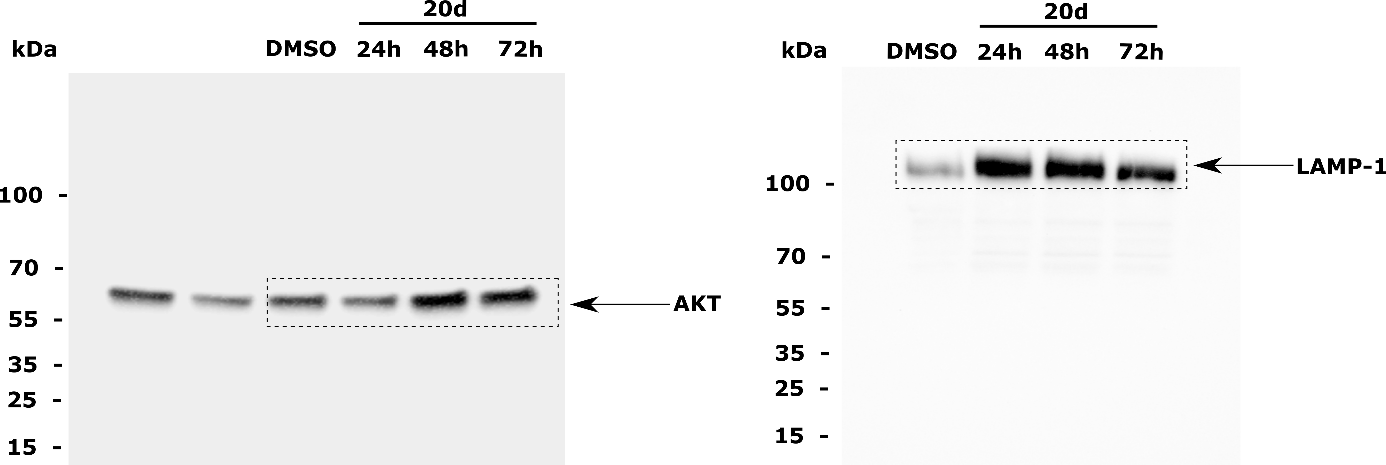


**Figure S57.** Full length western blot presented in **Figure 8** of the main article. Dotted boxes denote the cropped regions of the blots.


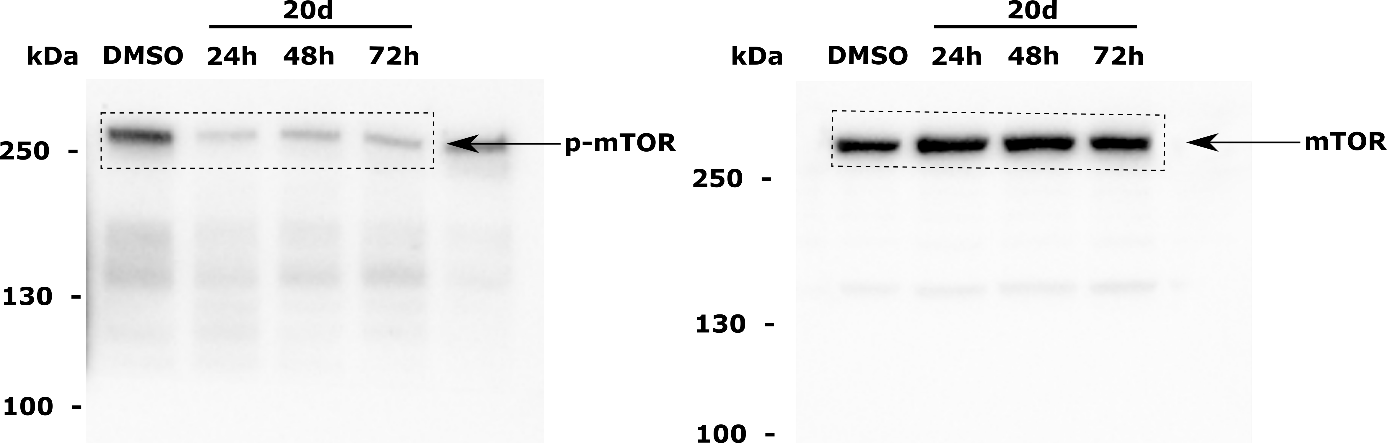


**Figure S58.** Full length western blot presented in **Figure 8** of the main article. Dotted boxes denote the cropped regions of the blots.


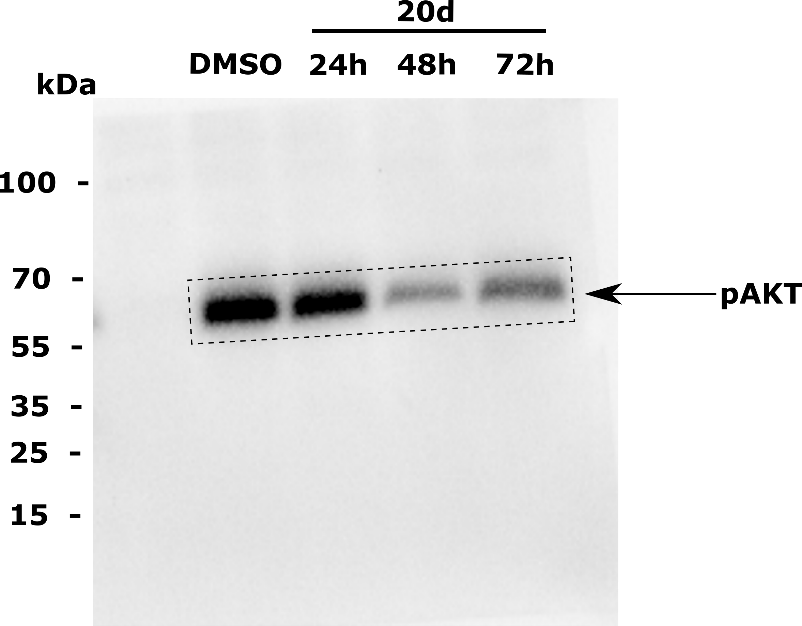


**Figure S59.** Full length western blot presented in **Figure 8** of the main article. Dotted boxes denote the cropped regions of the blots.


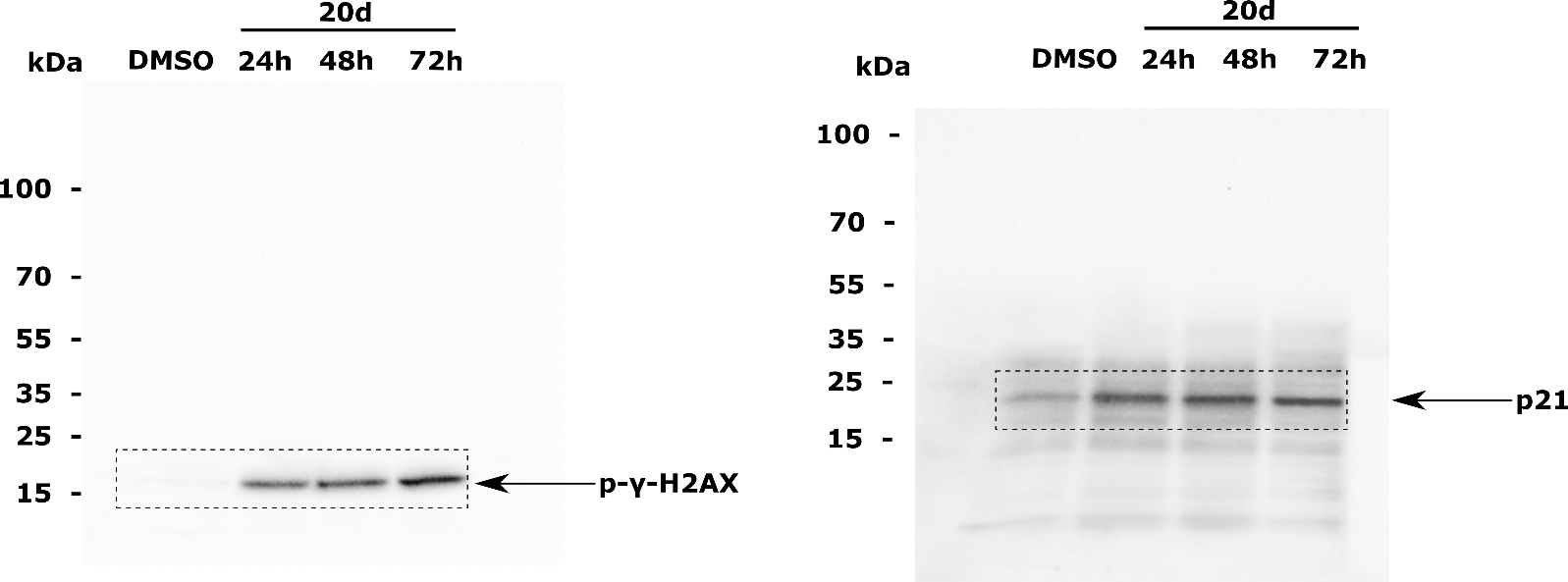


**Figure S60.** Full length western blot presented in **Figure 9** of the main article. Dotted boxes denote the cropped regions of the blots.


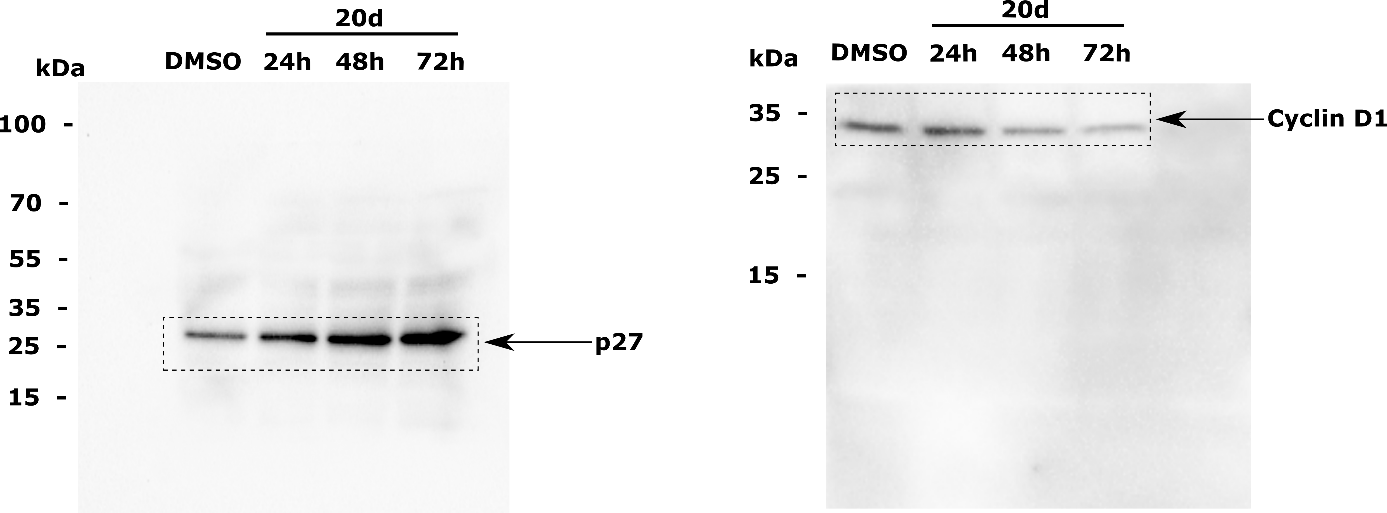


**Figure S61.** Full length western blot presented in **Figure 9** of the main article. Dotted boxes denote the cropped regions of the blots.


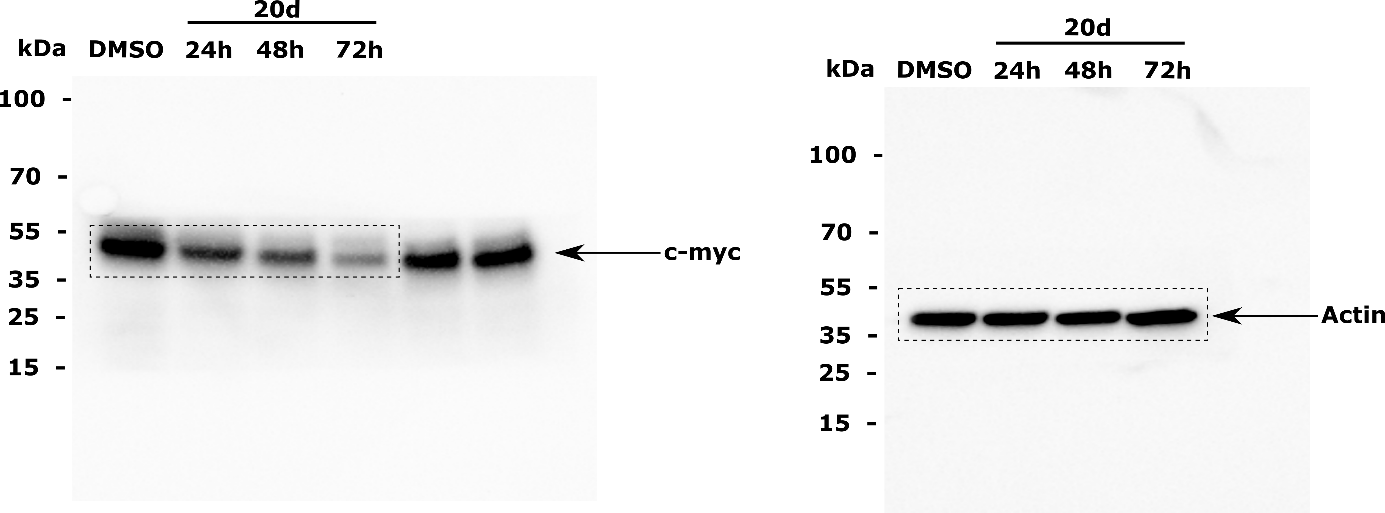


**Figure S62.** Full length western blot presented in **Figure 9** of the main article. Dotted boxes denote the cropped regions of the blots.


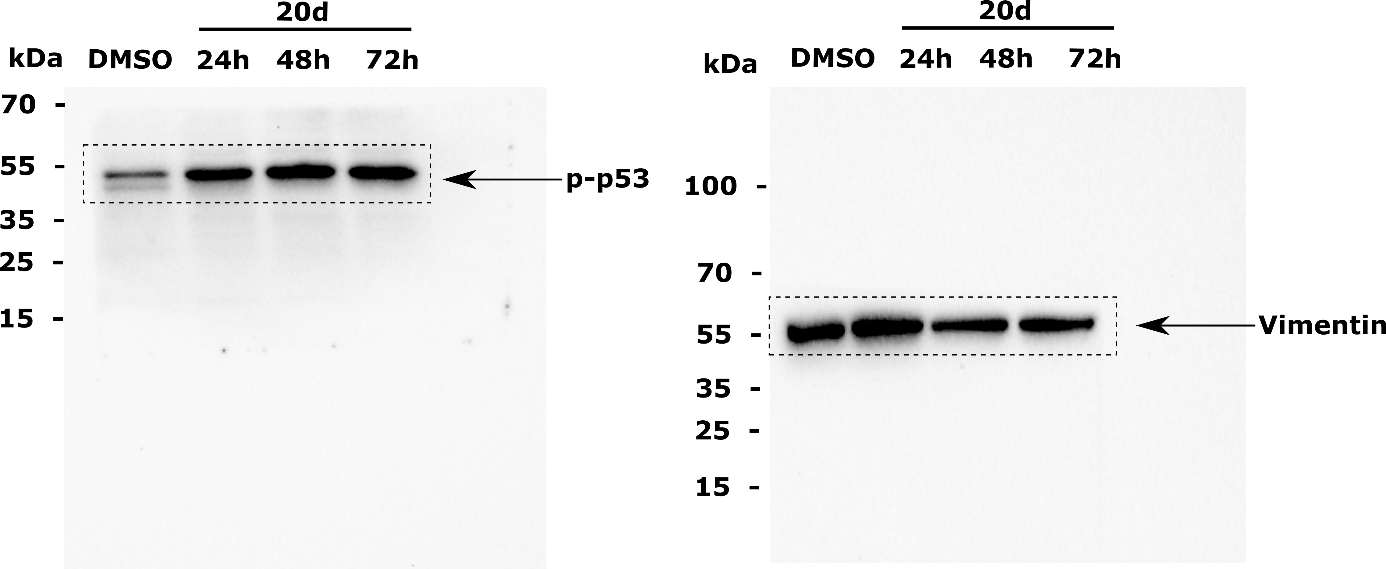


**Figure S63.** Full length western blot presented in **Figure 9** of the main article. Dotted boxes denote the cropped regions of the blots.


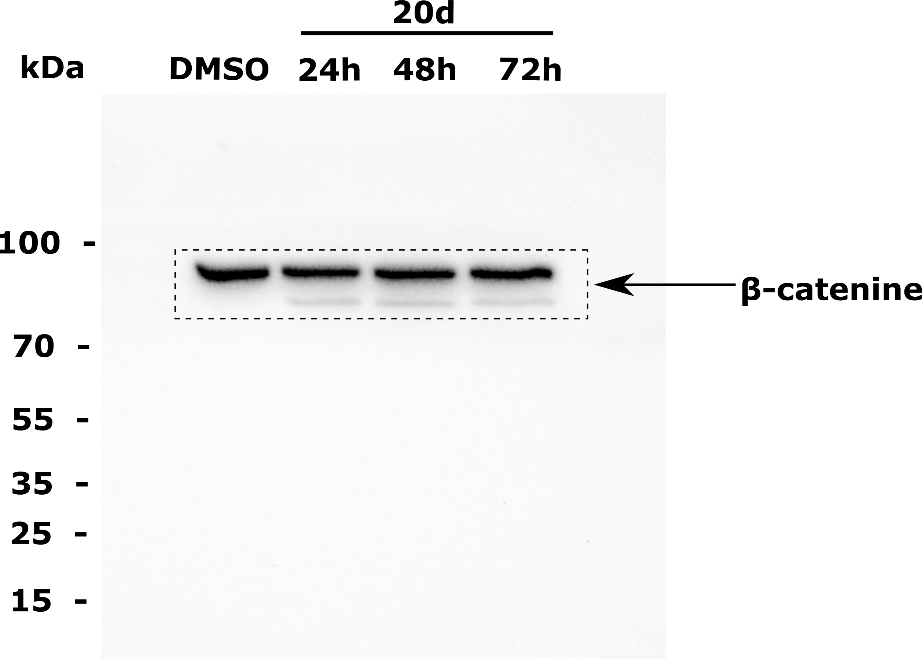


**Figure S64.** Full length western blot presented in **Figure 9** of the main article. Dotted boxes denote the cropped regions of the blots.

**
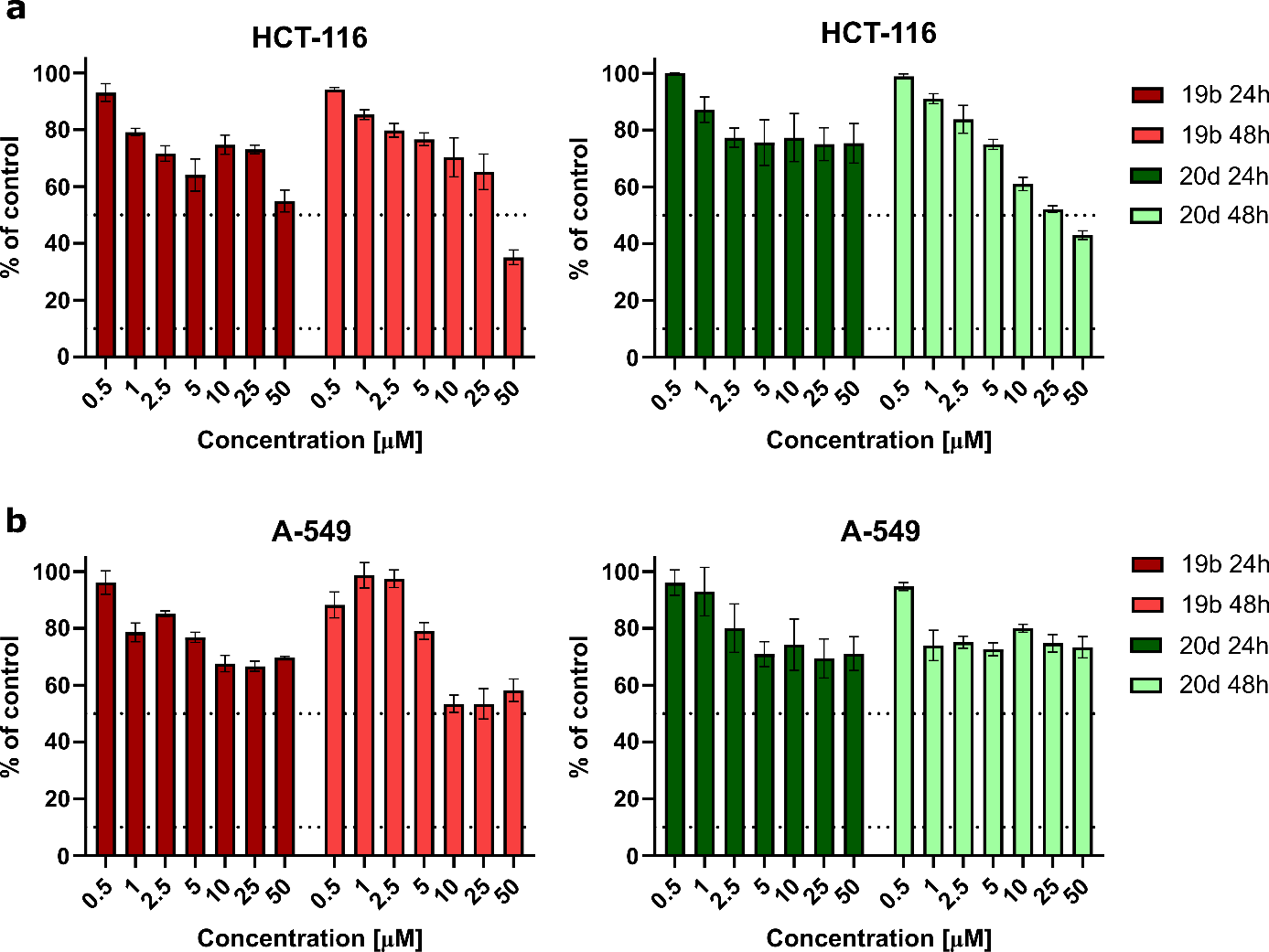
**

**Figure S65.** Effect of 19d and 20d on HCT-116 (**a**) and A-549 (**b**) cell viability after 24 and 48h of treatment. Data are presented as a mean ± SD.

**REFERENCES**

1. Huilai Y., Jie M., Xuexi S., A method of preparing of blonanserin, CN 104447551 (2015).
2. Lyle M. P. A., Wilson P. T., Synthesis of a new chiral nonracemic C2-symmetric 2,2‘-bipyridyl ligand and its application in copper(I)-catalyzed enantioselective cyclopropanation reactions *Org. Lett*, **6**, 855-857 (2004).
3. Morel A. F., Larghi L. E., Selvero M. M., Mild, efficient and selective silver carbonate mediated O-alkylation of 4-hydroxy-2-quinolones: Synthesis of 2,4-dialkoxyquinolines, *Synlett,* **18**, 2755-2758 (2005).
